# Effectiveness of economic support, comprehensive sexuality education and community dialogue on early childbearing and sitting for grade nine exams among adolescent girls in rural Zambia: a cluster randomised trial

**DOI:** 10.1016/j.eclinm.2024.102934

**Published:** 2024-11-15

**Authors:** Ingvild Fossgard Sandøy, Mweetwa Mudenda, Hanne Keyser Hegdahl, Joseph Mumba Zulu, Taran Grønvik, Ecloss Munsaka, Choolwe Jacobs, Joar Svanemyr, Bertil Tungodden, Astrid Blystad, Linda Kampata Olowski, Mpundu Chikoya Makasa, Karen Marie Moland, Ottar Mæstad, Amani Thomas Mori, Knut Martin Fylkesnes, Patrick Musonda

**Affiliations:** a Centre for Intervention Science in Maternal and Child Health (CISMAC), Norway; b Centre for International Health, Department of Global Public Health and Primary Care, University of Bergen, Postbox 7804, N-5020, Bergen, Norway; c Department of Public Health, Lusaka Apex Medical University, Zambia; d School of Public Health, University of Zambia, PO Box 50110, Lusaka, Zambia; e School of Education, University of Zambia, Great East Road Campus, PO Box 32379, Lusaka, Zambia; f Chr. Michelsen Institute, P.O.Box 6033, N-5892, Bergen, Norway; g Norwegian School of Economics, Helleveien 30, N-5045, Bergen, Norway

**Keywords:** Adolescent childbearing, Education, Cash transfers, Comprehensive sexuality education, Community dialogue

## Abstract

**Background:**

Adolescent pregnancies pose a risk to young mothers and their babies. In Zambia, one third of 18-year-old girls have given birth. Poverty, low secondary school enrolment, misinformation, and community norms contribute to early childbearing. We assessed the effectiveness of economic support alone and combined with comprehensive sexuality education (CSE) and community dialogue on childbearing and sitting for grade nine exams among girls.

**Methods:**

This cluster randomised trial had one control and two intervention arms. In 2016, 157 rural schools (units of randomisation) were included, and girls in grade seven were eligible. After recruitment, schools were stratified by district and randomised (ratio 31:63:63). In one arm, girls and their guardians were offered economic support. Another arm combined CSE and community dialogues with the economic support. The interventions were implemented for 27 months, and the participants were followed for another 2 years. The primary outcomes were *birth within8 monthsof the end of the intervention period, birth before the 18thbirthday,* and *sitting for grade nine exams*. The trial is registered with ISRCTN (ISRCTN12727868).

**Findings:**

Between 3rd March and 15th July 2016, 4922 girls were recruited, with 999 randomised to the control, 2004 to the economic and 1919 to the combined arm. The combined support gave a moderate reduction in the incidence of birth within eight months of the intervention period's end (risk difference (RD) −0.042 (95% CI −0.084, −0.0003), possibly a minimal reduction in birth before the 18th birthday (RD −0.010 (95% CI −0.053, 0.032), and a substantial increase in sitting for grade nine exams (RD 0.17 (95% CI 0.11, 0.22). The economic support alone tended to give slightly smaller effects.

**Interpretation:**

Our findings suggest that a combination of economic support, CSE and community dialogue may give small reductions in adolescent childbearing. The same interventions can increase completion of basic education.

**Funding:**

The 10.13039/501100005416Research Council of Norway, the 10.13039/501100005036University of Bergen and the 10.13039/100004441Swedish International Development Cooperation Agency (SIDA).

**Research in context**

**Evidence before this study**

We conducted a systematic search in six databases (Medline, Embase, Web of Science, PsycINFO, ERIC, and Cochrane) for randomised controlled trials (RCTs) conducted in low- and middle-income countries (LMICs) that measured the effectiveness of school or community-based interventions on adolescent childbearing. The search query included “Adolescent or teen or youth or girls”, “pregnan∗ or birth∗”, “intervention or program or trial or experiment” and “random∗” (Table S1). The last search was done on 10th November 2023, and 15,998 publications were found after removal of duplicates. First, we identified ten systematic reviews, covering publications up to January 2022 (Table S2). From these reviews we identified relevant original papers. In addition, we screened titles and abstracts of articles published after the latest systematic review which did not have restrictions on intervention types (Table S1). In total we identified 27 relevant RCTs (Table S3).

Several RCTs have examined the effects of poverty-alleviating interventions. Provision of free uniforms, payment of school expenses, and cash transfers have been found to reduce childbearing, but the effect sizes appear to depend on the context and on whether payment requires fulfilment of certain conditions.

Comprehensive sexuality education (CSE) has been found to reduce childbearing in a couple of trials when CSE was combined with vocational training or youth-friendly health services and community meetings. However, most trials in LMICs among young and mid-adolescents have not found effects, whereas a few evaluations among older adolescents (mean age >17) have found increased pregnancy rates. Several of the trials should be interpreted with caution due to methodological weaknesses or low intervention coverage, but overall, they indicate that the effectiveness of CSE may depend on the age of the target group and the context.

**Added value of this study**

To our knowledge, this is the first trial to examine the effects of economic support alone and in combination with CSE and community dialogue on adolescent childbearing and participation in junior secondary school exams in a LMIC context with economic, social and school capacity barriers to secondary schooling. Two years after the support ended the data was most compatible with the combined support causing a small reduction in early childbearing and a moderate increase in sitting for grade nine exams.

**Implications of all the available evidence**

Economic support combined with CSE and community dialogue may possibly have small effects on adolescent childbearing and moderately increase completion of basic education.

## Introduction

Recent estimates indicate that 14% of girls globally and 26% in sub-Saharan Africa have given birth before their 18th birthday.^1^ This represents a 30–40% reduction since 2000,^2^ but is still of great concern since pregnant girls and their children have a higher risk of complications and death.^3^ Early childbearing is further associated with negative social consequences, including early marriage and school dropout.^4^^,^^5^

In many societies girls who quit school early are more likely to become pregnant than those who are still enrolled,^4^ partly because they are regarded as ready for marriage and childbearing.^5^^,^^6^ However, not completing secondary education reduces the future health and socio-economic prospects of girls and their children.^7^

Poverty contributes to early school dropout,^8^ girls engaging in sexual relationships to receive gifts,^9^ becoming pregnant and getting married early in sub-Saharan Africa. Sociocultural factors also contribute to early childbearing. Parents and teachers are typically uncomfortable to communicate about sexuality, and this contributes to the spread of misinformation regarding fertility and modern contraceptives.^10^ Barriers to accessing modern contraceptives, and limited agency to negotiate sex and condom use, also increase the risk of unintended pregnancy. There is thus a need for interventions which target both economic and sociocultural factors that expose girls to the risk of unintended pregnancy and school drop-out and which simultaneously strengthen girls’ agency to make decisions. Randomised controlled trials (RCTs) in low- and middle-income countries (LMICs) on poverty-alleviating interventions such as cash transfers or payment of school expenses have found reduced childbearing and increased schooling among adolescent girls.^11^, ^12^, ^13^ However, the effectiveness of cash transfers appears to depend on the context and whether certain conditions must be fullfilled.^14^^,^^15^ There is also evidence that the effects of cash support on childbearing may diminish when the support ends.^15^

Randomised evaluations of comprehensive sexuality education (CSE) in LMICs have found effects on sexual debut,^13^ while the effects on childbearing among young and mid-adolescents have been mixed.^12^^,^^13^^,^^16^, ^17^, ^18^ Since several of the trials should be interpreted with caution due to methodological weaknesses,^13^ there is a need for more quality evaluations of CSE with high coverage. There is also a lack of evidence on what interventions have the potential to change restrictive social norms around sexuality and contraceptive use which prevent youth from utilising knowledge and skills acquired from CSE.^19^

In Zambia, where this study was implemented, one third of adolescents have given birth by the age of 18, and the incidence is particularly high among rural girls out-of-school.^20^ Net primary school enrolment was approximately 90% in 2015, but the transition to secondary school was <65% due to restricted number of school places.^21^ In 2015, the Zambian government increased the number of junior secondary schools (grades 8–9), and from 2018, everyone sitting for grade seven exams (end of primary school) could enrol in grade eight. However, for the 60% living below the poverty line, fees and other costs remained an important barrier. The government has implemented various cash transfer programmes to alleviate extreme poverty. RCTs indicate that this support increased school enrolment among older children,^22^ but evidence of effects on childbearing and marriage among adolescents has not been found.^23^

Our formative research indicated how sociocultural factors impact sexuality-related communication and pregnancy-related decisions in Zambia.^8^ A UNESCO review in 2012 identified major gaps in the official Zambian sexuality education, with limited focus on sexuality, communication, decision-making and gender.^24^ In 2014, the Ministry of General Education (MoGE) developed a CSE curriculum for grades 5–12. However, due to resource constraints, the training offered to most teachers was of low quality, and it took several years before teaching materials were produced.

This paper describes the findings from a trial which studied the effects on adolescent childbearing and sitting for grade nine exams of empowering girls through 1) economic support alone, and 2) economic support in combination with CSE and community dialogue meetings. We examined two hypotheses: First, economic support would reduce early childbearing due to economic empowerment giving increased schooling, fewer early marriages, and less need to engage in sexual relationships for economic benefit; second, combining economic support with CSE and community dialogue would further decrease adolescent childbearing by weakening social norms accepting early childbearing outside marriage, strengthening girls’ agency and negotiation skills, and correcting misinformation about fertility and contraceptives.

## Methods

### Trial design

The Research Initiative to Support the Empowerment of girls (RISE) was a cluster randomised controlled trial conducted in Zambia with one control and two intervention arms and rural basic schools (offering grades 1–9) as randomisation units. It was implemented according to the protocol,^6^ with the few minor exceptions described below or in the Appendix.

### Study setting and participants

The included schools were from twelve districts in Southern and Central provinces. The participants were girls in grade seven.

### Inclusion/exclusion criteria

All registered female pupils in grade seven in the selected schools in 2016 were eligible if their parents consented and they assented or consented. Schools were only included if >80% of the eligible participants gave informed assent and their guardians consented.

### Interventions

The interventions were implemented for 27 months from September 2016 (term three of grade seven) until November 2018 (when participants were expected to complete grade nine). In all three study arms, girls were offered writing materials as an incentive to participate. No other interventions were offered in the control arm.

#### ‘Economic support’

In both intervention arms, girls were offered monthly cash transfers of ZMW 30 (≈3 USD in 2016–18), their guardians were offered annual cash grants of ZMW 350/year, and school fees were paid for girls who enrolled in grade eight and nine (up to ZMW 1500/year). The average fee paid per girl was $72/year, and the total of fees and cash constituted approximately 6.7% of total household expenditures. The cash transfers were unconditional for girls below 18 and conditional on school enrolment after the participants’ 18th birthday.

#### CSE and community dialogue

The second intervention arm combined economic support with (1) an after-school youth club offering CSE; and (2) community dialogue meetings. The trial participants and boys in the same class were invited to the youth club, even if they quit school. Meetings were held every fortnight during school terms. A detailed manual prescribed activities for 36 meetings lasting 1–1.5 h. Two fictional films about early pregnancy, early marriage, and various educational trajectories were developed and shown during meetings.

Community dialogue meetings with parents and the community at large were organized every second month. The topics included early childbearing and the value of education. The facilitators were instructed to use a dialogue approach to trigger reflection, and to follow a detailed manual with specified activities for 14–16 meetings. The two films were shown also in these meetings.

For each school a teacher and a community health assistant or a community health worker were trained to jointly run the youth club and community meetings. More than a third of the youth club meetings and all the community meetings were monitored and supported by project staff. As part of the monitoring, a form was filled in with quality indicators related to the organisation of the meeting, the quality of facilitation and level of participation (with 20 and 17 indicators for youth club and community meetings, respectively). Each indicator could receive a score from 0 to 2.

### Recruitment

Formal and informal community leaders were asked to support the trial before recruitment took place from March to July 2016. Guardians of girls in grade seven were asked to consent to their daughters’ participation. After this, the girls were informed and asked to assent (or consent for those ≥18 years).

### Data collection

All the tools were translated to Tonga, Nyanja, Bemba and Lenje (the major local languages in the study districts), back-translated and piloted to ensure that the content was comprehensible. Interviews were conducted by young female research assistants in one of these local languages or English, depending on the participant's preference. A baseline face-to-face interview conducted immediately after recruitment included questions on previous childbearing, household assets, and marriage.

The participants were interviewed every six months about school attendance, marital status, childbearing, and related topics. Nine follow-up rounds were conducted up to December 2020 (see timings in Table S4). From the fourth follow-up onwards, face-to-face interviews were combined with Audio Computer Assisted Self-Interviews (ACASI) to give the participants more privacy when responding to sensitive questions.

The guardians were interviewed at baseline about their educational attainment and in 2018 regarding household expenditures, communication, and attendance of RISE meetings.

### Outcomes

The study had three primary outcomes: *birth within eight months of the end of the intervention period*, *birth before the* 18th *birthday*, and *sitting for grade nine exams.* In this paper we also report on three of the secondary outcomes: *pregnancy before the* 18th *birthday*, *perceived community norms regarding adolescent pregnancy*, and *perceived community norms regarding education*.^6^ All the outcomes used self-reported information. We measured *birth within eight months of the end of the intervention period* (which implies conception during the intervention period) in two different follow-up rounds: 8–14 and 20–25 months after the interventions ended (40–45 and 52–57 months after the recruitment). Using data collected 8–14 months after the interventions ended we aimed to minimise recall bias regarding the date of delivery, while we expected the last interview round to minimise attrition as more resources were available. We focus on the estimates from the latter since it included a higher proportion of the participants and report the findings from the earlier round in the Appendix. See Table S5 for measurement times of other outcomes. Birth and pregnancy outcomes were measured as incidence rates with events being the first birth or conception within the period of interest. *Sitting for grade nine exams* was measured with self-reported data, complemented with a sensitivity analysis using information from the District Educational Board Secretary offices. Perceived community norms regarding education and adolescent pregnancy outside marriage were measured with statements about the participant's perceptions of her parents’ attitudes (see Table S6).

To explore potential causal mechanisms, we examined intervention effects on the following intermediate outcomes: communication with parents about romantic relationships or sex (fourth follow-up), being in school, educational attainment, total grade nine exam scores, and marital status. We also examined the association between being in school and recent sexual activity in the fifth follow-up.

### Sample size

We assumed 10% loss-to-follow-up, cluster sizes of 31, and an intraclass correlation (ICC) of 0.00737 for the birth outcomes and ≤0.02 for *sitting for grade nine exams*.^6^ With 31 clusters in the control, 63 clusters in each intervention arm and 95% confidence level, we expected to have 90% power to detect a ≥23% reduction in the economic vs control arm in births before the 18th birthday, and a further ≥23% reduction in the combined vs economic arm if there was a 25% reduction in the economic compared to the control arm.

### Randomisation and masking

After the recruitment was completed, the randomisation of schools was stratified by district and organized as six public ceremonies in mid-July 2016.

There was no blinding of the participants, but the interview team was independent from the intervention delivery team. We planned to keep interviewers in the final follow-up round unaware of which study arm schools belonged to, but it became difficult as the participants often referred to the support they had received. Thus, we retained research assistants with whom the participants were familiar to make them feel comfortable.^25^ The researchers were blinded during the primary outcome analysis, and the interpretation of the results was recorded before the randomisation code was broken.

### Statistical analysis

The data was analysed with Stata 17.0. We compared outcomes pairwise for the three arms. The primary analyses were by intention-to-treat and followed a predetermined analysis plan. All analyses took account of the design effect and stratified randomisation.

We had predetermined to adjust the regression models for imbalances of baseline confounders if they were associated with the outcome (see Appendix for details).

For the birth and pregnancy outcomes, we intended to use Cox regression. However, since the proportional hazards assumption did not hold, a parametric Weibull model was employed instead. Cox-model estimates are presented in the Appendix. For binary variables, we used generalized estimating equations (GEE) with a log link. Absolute effects (risk differences, RD) for the primary outcomes were calculated using generalized linear models (GLM) with an identity link.

To assess the potential impact of loss-to-follow-up, we conducted three sensitivity analyses: 1) assuming those who were married when lost to follow-up had given birth within the period of interest; 2) using proxy information on births from teachers, family, and friends; 3) using inverse probability weighting.

In the pre-analysis plan we had not considered induction time (i.e. time before interventions can affect the outcome). We did a post-hoc analysis to check whether 6- or 12-months induction time would affect the results. To explore whether the effects on the birth outcomes differed *during* vs *after* the intervention period, we estimated the effects on time to the first birth in each of the two periods. We also explored differential age-effects by stratifying by age at randomisation.

See Appendix for further details about the methodology.

### Ethics

The protocol was approved by the University of Zambia Biomedical Research Ethics Committee (ref no 021- 06-15) and the Regional Ethics Committee of Western Norway (ref no 2015/895).

### Role of funding source

The funders did not play any role in study design, management, data interpretation or paper writing.

## Results

**Fig. 1:**
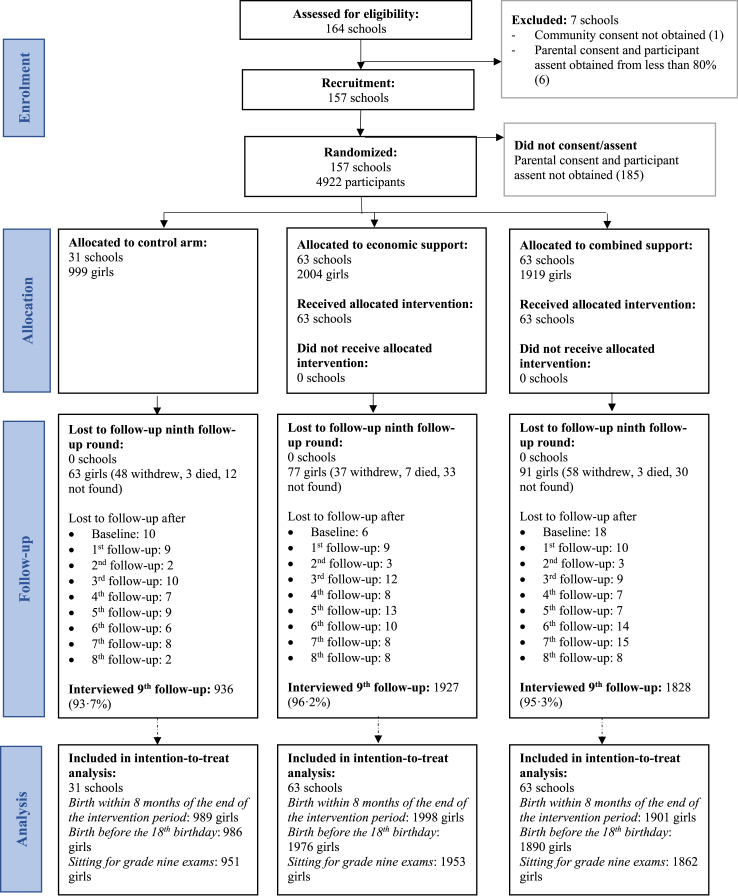
Flow diagram.

Between 3rd March and 14th July 2016 informed assent and consent were obtained for 4922 (96%) of the 5107 eligible participants and their guardians in the 157 included schools (Fig. 1). No clusters were lost to follow-up, and in the final (ninth) follow-up round, 95.3% of the participants were interviewed, with minimal differences between the arms (Table S7).

Most of the baseline characteristics were similar across the arms (Table 1). The rest were not associated with the outcomes and thus not judged to be confounders. The mean age was approximately 14 years (range 10–25 years), the majority had guardians with primary education or less, and poverty levels appeared high with more than half usually not having enough food in the household. The majority thought it was important to complete grade nine and perceived their parents to share this view. Less than 0.5% had given birth.

**Table 1:** Baseline characteristics of the participants by study arm.

	Control	Economic	Combined
**Number of clusters**	31	63	63
**Number of participants**	999	2004	1919
**Mean cluster size (range)**	32.2 (17, 68)	31.8 (13, 63)	30.5 (13, 69)
**Age (mean (SD))**	14.1 (1.29)	14.1 (1.38)	14.0 (1.32)
**Highest level of school of parent/guardian (n (%))** (missing values: 10)			
Lower primary or less	210 (21%)	372 (19%)	406 (21%)
Upper primary	410 (41%)	742 (37%)	683 (36%)
Junior secondary	198 (20%)	455 (23%)	468 (24%)
Senior secondary or more	176 (18%)	432 (22%)	360 (19%)
**Living with biological parents (n (%))** (missing values: 2)			
Yes	777 (78%)	1433 (72%)	1434 (75%)
**Biological mother alive (n (%))** (missing values: 96)			
Yes	929 (93%)	1831 (91%)	1757 (92%)
**Biological father alive (n (%))** (missing values: 107)			
Yes	841 (84%)	1655 (83%)	1561 (81%)
**Household has enough food (n (%))** (missing values: 5)			
Seldom or never	196 (20%)	396 (20%)	289 (15%)
Sometimes	354 (35%)	696 (35%)	720 (38%)
Usually	447 (45%)	910 (45%)	909 (47%)
**Repeated any grade (n (%))** (missing values: 1)			
No	548 (55%)	1186 (59%)	1111 (58%)
**My parents think it is important for me to complete grade nine (n (%))** (missing values: 0)			
Agree	800 (85%)	1648 (88%)	1549 (86%)
**If I complete grade nine, I will significantly increase my future income (n (%))** (missing values: 0)			
Agree	659 (66%)	1369 (68%)	1254 (65%)
**If I complete grade nine, I will benefit even if it doesn't increase my future income (n (%))** (missing values: 0)			
Agree	610 (61%)	1281 (64%)	1170 (61%)
**Girls <18 years are treated with more respect if they have a child (n (%))** (missing values: 0)			
Disagree	516 (52%)	1085 (54%)	1051 (55%)
**If I become a mother before I am 18, adults will treat me with more respect (n (%))** (missing values: 0)			
Disagree	869 (87%)	1745 (87%)	1658 (86%)
**If I become a mother before I am 18, I will be a significant economic burden to my family (n (%))** (missing values: 0)			
Agree	859 (86%)	1732 (86%)	1688 (88%)
**Better for me if I have a child before I am 18 than to wait (n (%))** (missing values: 0)			
Disagree	958 (96%)	1923 (96%)	1837 (96%)
**Ever had boyfriend (n (%))** (missing values: 8)			
No	893 (89%)	1814 (91%)	1768 (92%)
**Ever given birth (n (%))** (missing values: 1)			
No	996 (99.7%)	1994 (99.5%)	1911 (99.6%)

The coverage of the economic support was high with girls in the intervention arms signing for cash on average 24.5 of 27 intervention months. School fees were paid for 90% of the participants in 2017 and ≥76% in 2018. In the combined arm, 50% of girls indicated attending at least half of the 36 youth club meetings, and guardians appeared to attend half the community meetings (Table S8 and description of intervention coverage in Appendix). The monitoring reports indicated that meetings were implemented with high quality (average score 32.1/40 for youth club meetings and 29.0/34 for community meetings).

**Supplementary Figure S1:**
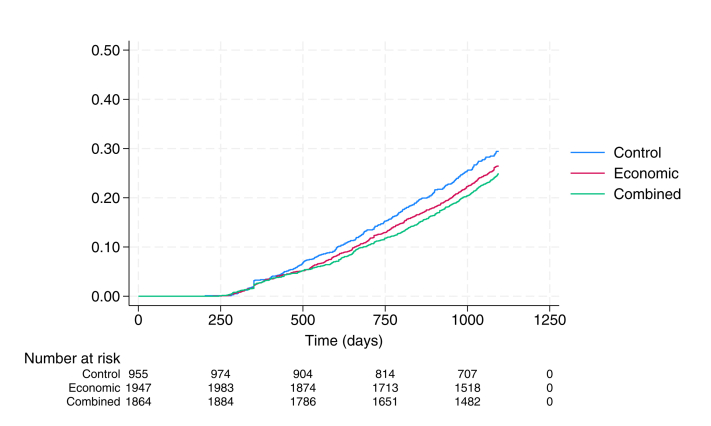
Cumulative incidence of giving birth within eight months of end of intervention period.

Compared to the control arm, the combined support moderately reduced the incidence of giving birth within eight months of the interventions ending with 18%, precisely estimated (HR 0.82; 95% CI 0.69–0.97) (Tables 2 and Table S9 and Figure S1). For the incidence of birth before the 18th birthday, our data was compatible with a similarly narrow range of intervention effects, including none; the most likely being small (HR 0.89; 95% CI 0.73–1.08). The reduction in pregnancy incidence before the 18th birthday appeared stronger (HR 0.81; 95% CI 0.68–0.97) (Table 3). There was a moderately large increase in the educational outcome sitting for grade nine exams (RR 1.26; 95% CI 1.16–1.37) (Table 2), and a reduction in the proportion of girls perceiving community norms to accept adolescent childbearing outside marriage (RR 0.83; 95% CI 0.71–0.96) (Table 3).

**Table 2:** Effects of the intervention packages on the primary outcomes.

	Control	Economic	Combined		Economic vs control	Combined vs control	Combined vs economic	ICC (95% CI)
*Birth within* 8 months *of the end of the intervention period*^a^								
n (%)	283/989 (28.6%)	520/1998 (26.0%)	466/1901 (24.5%)	RD (95% CI)	−0.025 (−0.068, 0.019)	−0.042 (−0.084, −0.0003)	−0.018 (−0.052, 0.016)	0.031 (0.018, 0.054)
				NNTB (95% CI)	40.7 (NNTH 51.9 to ∞ to NNTB 14.6)	23.6 (11.9–3384.1)	56.4 (NNTH 61.4 to ∞ to NNTB 19.3)	
Rate per person *year*	0.11	0.097	0.090	Age adj. HR (95% CI)	0.89 (0.75, 1.06)	0.82 (0.69, 0.97)	0.91 (0.79, 1.06)	
				p-value	0.200	0.022	0.239	
*Birth before the* 18th *birthday*^b^								
n (%)	283/986 (28.7%)	549/1976 (27.8%)	528/1890 (27.9%)	RD (95% CI)	−0.010 (−0.051, 0.032)	−0.010 (−0.053, 0.032)	−0.0004 (−0.037, 0.037)	0.033 (0.018, 0.059)
				NNTB (95% CI)	102.9 (NNTH 31.7 to ∞ to NNTB 19.6)	99.1 (NNTH 30.9 to ∞ to NNTB 19.0)	2695.4 (NNTH 27.2 to ∞ to NNTB 26.7)	
Rate per person *year*	0.097	0.091	0.091	HR (95% CI)	0.92 (0.76, 1.11)	0.89 (0.73, 1.08)	0.96 (0.82, 1.14)	
				p-value	0.396	0.225	0.653	
*Sat for grade nine exams*^c^								
n (%)	617/951 (64.9%)	1481/1953 (75.8%)	1513/1862 (81.3%)	RD (95% CI)	0.11 (0.049, 0.17)	0.17 (0.11, 0.22)	0.057 (0.016, 0.098)	0.080 (0.054, 0.12)
				NNTB (95% CI)	9.2 (5.9–20.5)	6.0 (4.5–9.2)	17.5 (10.2–61.5)	
				RR (95% CI)	1.17 (1.07, 1.28)	1.26 (1.16, 1.37)	1.08 (1.02, 1.14)	
				p-value	0.001	<0.001	0.005	

HR, hazard ratio; ICC, intracluster correlation; NNTB, number needed to benefit; NNTH, number needed to harm; RD, risk difference.

a Including data up to 52–57 months after recruitment/20–25 months after the end of the intervention period. 34 participants were lost-to follow-up after randomisation and not included in the effectiveness analysis.

b 36 participants were already aged 18 at the time of randomisation and were excluded, whereas 46.5% of the participants were below 18 at the time of the last interview and were censored. 34 participants were lost-to follow-up after randomisation and not included in the effectiveness analysis.

c Information available from follow-up rounds six to nine.

**Table 3:** Effects of the intervention packages on the secondary outcomes.

	Control	Economic	Combined		Economic vs control	Combined vs control	Combined vs economic	ICC (95% CI)
*Pregnancy before the* 18th *birthday*^a^								
n (%)	398/986 (40%)	740/1976 (37%)	687/1890 (36%)	RD (95% CI)	−0.029 (−0.076, 0.019)	−0.045 (−0.093, 0.003)	−0.016 (−0.057, 0.025)	0.035 (0.021, 0.057)
				NNTB (95% CI)	34.9 (NNTH 52.4 to ∞ to NNTB 13.1)	22.3 (NNTH 333.7 to ∞ to NNTB 10.8)	62.2 (NNTH 40.3 to ∞ to NNTB 17.5)	
Rate per person *year*	0.15	0.14	0.13	HR (95% CI)	0.88 (0.74, 1.05)	0.81 (0.68, 0.97)	0.92 (0.79, 1.07)	
				p-value	0.150	0.021	0.290	
*Perceived community norms to accept early pregnancy outside marriage*^b^								
n (%)	351/852 (41%)	656/1795 (37%)	595/1737 (34%)	RR (95% CI)	0.89 (0.79, 1.02)	0.83 (0.71, 0.96)	0.93 (0.78, 1.09)	0.12 (0.080, 0.16)
				p-value	0.087	0.015	0.359	
*Perceived community norms to support girls completing grade nine*^c^								
n (%)	85/777 (11%)	266/1637 (16%)	220/1559 (14%)	RR (95% CI)	1.05 (1.02, 1.09)	1.06 (1.03, 1.10)	1.01 (0.98, 1.04)	0.087 (0.054, 0.14)
				p-value	0.006	<0.001	0.527	

HR, hazard ratio; ICC, intracluster correlation; NNTB, number needed to benefit; NNTH, number needed to harm; RR, risk ratio.

a 36 participants were already aged 18 at the time of randomisation and were excluded, whereas 46.5% of the participants were below 18 at the time of the last interview and were censored.

b Information available from fifth follow-up round. Of the 4397 interviewed in this round, 13 refused to answer one of the two questions on pregnancy norms.

c Information available from fifth follow-up round. Of the 4397 interviewed in this round, 22 refused to answer one of the three questions on norms regarding girls completing grade nine and 402 did not have a mother or father (see Appendix).

The data was most compatible with economic support leading to 8–12% relative reductions in the incidence of all three childbearing outcomes but could also be consistent with no effect (Table 2, Table 3). The economic support increased sitting for grade nine exams (RR 1.17; 95% CI 1.07–1.28), tended to reduce the perception that community norms accepted adolescent childbearing (RR 0.89; 95% CI 0.79–1.02), and increased the perception that norms supported girls completing grade nine (RR 1.05; 95% CI 1.02–1.09).

For the birth and pregnancy outcomes there were negligible to small differences between the two intervention arms (Tables 2, 3 and Table S9). The percentage sitting for grade nine exams was slightly higher in the combined vs the economic arm (RR 1.08; 95% CI 1.02–1.14).

The Cox model (Table S10) and the sensitivity analyses considering induction time (Tables S11a and S11b) and loss to follow-up (Table S12a, S12b, and S12c) gave almost the same effect estimates as the main analysis (Table 2, Table 3). We found a low proportion being inconsistent in the reporting of births (see Appendix). Data from the exam registers indicated similar relative effect sizes for sitting for grade nine exams as the self-reported information (Table S13). The ICC was approximately 0.03 for the childbearing outcomes and 0.08 for grade nine exams (Table 2).

The posthoc analyses indicated that the effect *during* the intervention period of the combined support on the birth incidence was moderate (HR 0.75; 95% CI 0.60–0.94 for birth before the 18th birthday) and the effect of the economic support was smaller, whereas the birth rates increased and the data was most compatible with no protective effect *after* the cessation of the two types of support (Table S14).

In subgroup analyses, the combined and economic support had greater effects on giving birth during the intervention period, sitting for grade nine exams, and perceived pregnancy norms, among those who were aged 16–17 at randomisation (aged 18–19 at the end of the intervention period) than the younger participants (Table S15). The exploratory mediation analyses indicated that being in school was strongly associated with a lower probability of reporting recent sexual activity at the end of the intervention period in all three arms (Table S16a). In all follow-up rounds, the economic support increased the probability of being in school, resulting in increased educational attainment (Table S16b and Figure S3). The economic support also increased the self-reported grade nine exam score (Table S17). The addition of CSE and community dialogue had minimal effects on these school outcomes. The combined support increased communication about romantic relationships or sex with guardians, possibly reduced the proportion who was married or currently had a boyfriend, and increased the agency among those who were in a relationship to initiate conversations with their partner about contraceptives (Table S18). Only 13.5% of those who reported becoming pregnant were married at the time of conception, and 2/3 of those who married were already pregnant (Table S19).

## Discussion

Empowering girls through a combination of economic support, CSE and community dialogue possibly resulted in moderate reductions in births conceived during the intervention period, but only small reductions in childbearing before age 18. The economic component reduced financial and social barriers to continued schooling, resulting in fewer girls reporting sexual activity.^26^ The combined support also affected sociocultural factors that typically shape fertility and educational decisions such as girls’ agency and perceived social norms pertaining to early pregnancy.

The posthoc analysis suggested that the effects might have been larger if measured immediately after the interventions ended rather than 1.5–2 years later, and it is plausible that stronger reductions in early childbearing would have occurred if the support had continued until the participants turned 18. The process evaluation indicated that the economic support was highly valued by the recipients. They felt motivated to focus on school and did not need boyfriends to get cash.^27^ Correspondingly we expect that the end of the economic support had noticeable effects for them. In fact, the effects on childbearing appeared to diminish already in the last months of the intervention period. This was possibly because many participants knew they were unlikely to continue schooling the following year since the support would end, and this may have made them less worried about engaging in sexual relationships and getting pregnant. After the support ended, the participants may also have been motivated to have a boyfriend for economic reasons.

In addition to the direct short-term effect of the economic support on the ability to pay school expenses, the findings that the relative effects on schooling persisted beyond the intervention period may reflect indirect effects via academic performance and social support. The perceived normative change regarding girls’ education may have occurred because the girls and their guardians highly valued the economic support, and during the information meetings guardians encouraged each other to back their daughters’ schooling to make good use of the unique opportunity the support provided.

We found that the addition of CSE and community dialogue to economic support did not have large effects on childbearing, perceived norms, or school outcomes. This was despite the CSE component being in line with UNESCO's recommendations regarding content, duration, intensity, interactive teaching, facilitator compentency,^28^ implementation fidelity (according to reports from the frequent monitoring visits), and the parallel community dialogues addressing restrictive norms. During community meetings, the members were given opportunities to deliberate and make public promises of change, processes that may facilitate the establishment of new collective normative beliefs.^29^ However, fundamental normative changes commonly take time and may depend on more permanent structural changes such as reduced social inequality.^8^ Substantial contamination due to the MoGE curriculum seems unlikely as just a handful of teachers in the included schools had received CSE training. Although we did not evaluate CSE on its own, our findings strengthen the impression that CSE may not be enough to reduce early childbearing,^12^^,^^13^^,^^18^ indicating the complex interplay of sociocultural influences. Combining CSE with better contraceptive access might have given larger effects.^28^^,^^30^

The effects of economic support on childbearing were not mediated through early marriage. Rather, pregnancy appeared to be a driver of the latter, in line with previous studies,^5^ probably to have a father to support the child and avoid the stigma of single motherhood.

We found indications that the effects of the combined and economic support on childbearing, sitting for grade nine exams and perceived community norms were stronger among the older than the younger participants. This may reflect that they were delayed in their school progression (the expected age in grade seven is 13–14 years) and at higher risk of school dropout and pregnancy.^4^ Combined with the conditional nature of the cash transfers for those aged ≥18, this may have contributed to the older participants being particularly motivated to exploit the support.

Previous trials on CSE have been affected by methodological weaknesses or low intervention coverage. The percentage that assented and consented in this trial was very high, randomisation gave balanced distributions of baseline characteristics, and attrition was very low in all three arms. Since the intervention delivery and outcome assessment teams were independent, we believe that the lack of blinding is unlikely to have substantially biased the effect estimates. In combination with the high intervention coverage, we believe that the trial provides valid estimates of the effectiveness of the studied interventions. Thus, we argue that this trial makes an important contribution to the evidence base on CSE, community dialogue and economic support in preventing adolescent childbearing.

Although the trial included a high number of clusters, the estimated effect sizes were considerably smaller and the ICCs were larger than we had assumed, resulting in some uncertainty regarding the effects. Another potential challenge was bias in the self-reporting of outcomes, but since there were few inconsistencies in the reporting of births and girls appeared proud to become mothers,^8^ we do not think this substantially affected our findings.

The trial findings are likely to be relevant to other contexts where early childbearing is common and there are substantial hurdles to secondary school enrolment and sociocultural barriers to discussing SRH with adolescents. It is important to note that since 2022, the Zambian government has made education up to grade 12 free of charge, and in line with our findings, this has led to substantial increases in secondary school enrolment.

Our findings and those of similar studies clearly indicate that removing economic barriers to schooling is important to allow more children to complete basic education. However, the effects of economic support on adolescent childbearing appear to differ between contexts. Empowering girls through better negotiation and communication skills, more knowledge on SRH and a more supportive environment, appear to be helpful additions to achieve notable reductions in adolescent birth rates during the support period. The minimal to modest overall effects may reflect that the duration of the support was too short, given the many financial and structural challenges in achieving change within the fundamental life spheres of sexuality and reproduction. Additional approaches, such as better access to youth friendly health services and contraceptives, are probably needed to achieve larger reductions in adolescent childbearing.^28^^,^^30^

## Contributors

IFS was the principal investigator for the trial, PM was the local principal investigator, and MM was the project coordinator. IFS, BT, OM, JMZ, AB, PM, KMM, EM, MM, KMF, MCM, CJ and LKO conceived and designed the trial. JMZ, EM, JS, IFS, MM, PM, AB, KMM, MCM and CJ developed the intervention manuals. IFS, HKH, BT, OM, ATM, AB, PM, TG, KMM, EM, JMZ, MM, KMF, CJ, LKO, and MCM developed the tools. IFS, HKH, TG and MM cleaned the data. IFS conducted the statistical analysis with support from PM, HKH and TG. HKH and TG accessed and verified the underlying data. All authors contributed to the creation of study instruments or the data collection. IFS drafted the original manuscript, and all authors interpreted the findings, critically reviewed the manuscript, and approved the final version. All authors had permission to access the underlying data.

## Data sharing statement

Deidentified data is available on request for health research from the date of publication of this article and for the following 5 years. Requests for data should be made to Ingvild Fossgard Sandøy (ingvild.sandoy@uib.no) and Patrick Musonda (pmuzho@hotmail.com) with a research proposal approved by an independent review board.

## Declaration of interests

We declare no competing interests.

## Figures and Tables

**Supplementary Figure S2:**
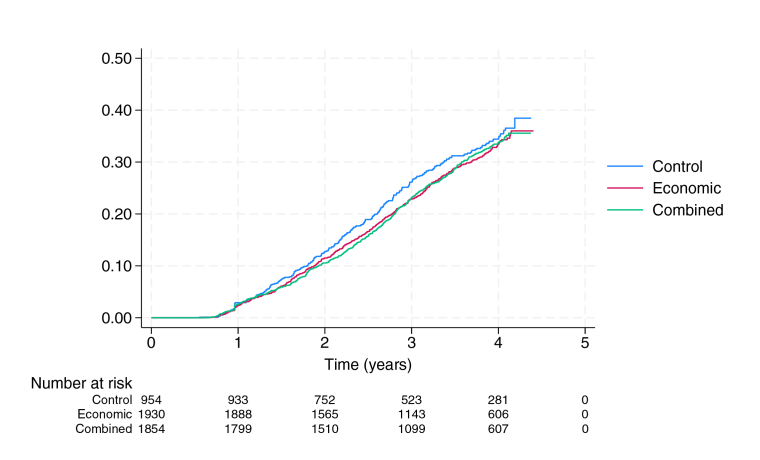
Cumulative incidence of giving birth before 18th birthday.

## Appendix A Supplementary data

Changes to the protocol

Supplementary Material

Trial protocol
