# Supplementary material for: Effectiveness of economic support, comprehensive sexuality education and community dialogue on early childbearing and sitting for grade nine exams among adolescent girls in rural Zambia: a cluster randomised trial
Source: eClinicalMedicine. 2024 Nov 15;78:102934. doi: 10.1016/j.eclinm.2024.102934 (PMC11609475; doi:10.1016/j.eclinm.2024.102934)
Supplement: Changes to the protocol [file mmc1.docx]

**Changes to the protocol**

The following changes were made to the protocol after the start of trial recruitment in March 2016:

**Study setting and size**

The average cluster size turned out to be smaller than anticipated (31 instead of 37) and in some of the schools we had invited, we did not obtain assent/consent from a sufficient proportion of the eligible participants (see Figure 1). We compensated by including 157 (instead of 153) schools. The number of study districts was increased from 8 to 12 to find enough schools that were at least 8 km apart. (This change was made in May 2016, i.e. before randomization).

**Outcomes**

- Changed birth measures from proportions to incidence rates (in July 2016)
- Added the secondary outcome “Socioeconomic inequality in incidence of marriage/ cohabitation before girls’ 18th birthday” (in November 2016).
- Changed marriage measures from proportions to incidence rates (in October 2020) as we realized it should be measured in a similar way to the birth outcomes.

**Interventions**

- The number of annual community meetings in the combined arm was increased from four to six (change made in July 2016, i.e. before the start of the intervention period)

**Follow-up interviews**

- The main interview mode changed from being via telephone to being face-to-face because it turned out to be more difficult than anticipated to reach the participants via phone since network problems were common, most of them did not have their own phones, and they were often not together with their guardians (who had phones) during the day.
- Audio Computer Assisted Self-Interviewing was employed for sensitive questions from the fourth follow-up round.
- The transport allowance/compensation provided for participating in the follow-up rounds was increased from ZMW 20 to ZMW 50 in 2018, ZMW 75 in 2019 and ZMW 100 in 2020 because an increasing proportion of the participants moved away.
- We obtained additional funding to interview guardians about household expenditures in 2018.
- From 2019 the research assistants were instructed to take selfies of themselves with the participants to document that they had interviewed the correct participants (and had not been tempted to fabricate interviews when they were asked to visit the participants´ homes without a supervisor accompanying them).
- From 2019 we recorded information from teachers, neighbours and family members on marital status and childbearing of participants who were lost to follow-up to use this information in sensitivity analyses.

All the changes were approved by the ethics committees.
