# Supplementary material for: Effectiveness of economic support, comprehensive sexuality education and community dialogue on early childbearing and sitting for grade nine exams among adolescent girls in rural Zambia: a cluster randomised trial
Source: eClinicalMedicine. 2024 Nov 15;78:102934. doi: 10.1016/j.eclinm.2024.102934 (PMC11609475; doi:10.1016/j.eclinm.2024.102934)
Supplement: Supplementary Material [file mmc2.docx]

Supplementary material

Contents

[Systematic literature search 4](#_Toc182216283)

[**Table S1: Search queries in each database** 4](#_Toc182216284)

[**Table S2: Identified systematic reviews on the effectiveness of interventions on adolescent childbearing** 5](#_Toc182216285)

[**Table S3: Identified relevant randomised controlled trials evaluating the effectiveness of interventions on adolescent childbearing in LMICs** 6](#_Toc182216286)

[Protocol deviations 8](#_Toc182216287)

[Trial design 8](#_Toc182216288)

[Recruitment and randomisation 8](#_Toc182216289)

[Intervention details 9](#_Toc182216290)

[**Table S4: Timing of interview rounds** 10](#_Toc182216291)

[Detailed information about the definition of outcomes 10](#_Toc182216292)

[**Table S5: Outcomes included in this paper** 10](#_Toc182216293)

[**Table S6: Questions used to measure each of the perceived community norm outcomes and description of how the responses were scored** 11](#_Toc182216294)

[Data management 12](#_Toc182216295)

[Details about the statistical analysis 12](#_Toc182216296)

[Ethical aspects 13](#_Toc182216297)

[Role of the funding source 13](#_Toc182216298)

[Supplementary findings 14](#_Toc182216299)

[**Table S7: Number and percentages of participants interviewed in each of the follow-up rounds** 14](#_Toc182216300)

[**Intervention coverage** 14](#_Toc182216301)

[**Table S8: Guardians’ attendance of RISE meetings in previous six months according to parent interviews in 2018** 14](#_Toc182216302)

[**Figure S1: Cumulative incidence of giving birth within eight months of end of intervention period** 15](#_Toc182216303)

[**Figure S2: Cumulative incidence of giving birth before 18th birthday** 16](#_Toc182216304)

[**Table S9: Effects of the intervention packages on giving birth within eight months of the end of the intervention period (using data collected up to 40-45 months after the randomisation/8-14 months after the end of the intervention period)** 17](#_Toc182216305)

[**Inconsistencies in reporting of births** 18](#_Toc182216306)

[**Table S10: Effects of the intervention packages on birth and pregnancy outcomes using Cox regression (as mentioned in the data analysis plan)** 18](#_Toc182216307)

[**Table S11a. Sensitivity analysis of the effects of the intervention packages taking six months induction time into account** 19](#_Toc182216308)

[**Table S11b: Sensitivity analysis of the effects of the intervention packages taking 12* months induction time into account** 20](#_Toc182216309)

[**Table S12a: Sensitivity analyses of the effects of the intervention packages on composite birth and marriage outcomes** 21](#_Toc182216310)

[**Table S12b: Sensitivity analyses of the proportions who had given birth by the end of the trial with and without proxy information on births** 21](#_Toc182216311)

[**Table S12c: Effects of the intervention packages on outcomes using inverse probability weighting to adjust for loss-to-follow-up** 22](#_Toc182216312)

[**Table S13: Effects of the intervention packages on the probability of sitting for grade nine exams according to exam registers from the DEBS offices** 23](#_Toc182216313)

[**Table S14: Posthoc analysis of effects of the intervention packages on giving birth during and after the intervention period** 23](#_Toc182216314)

[**Table S15: Age-specific effects of the intervention packages on norms, sitting for grade nine exams and birth during the intervention period at randomisation** 24](#_Toc182216315)

[**Table S16a: Association between reporting sexual activity previous 4 weeks and being in school in the fifth follow-up** 25](#_Toc182216316)

[**Table S16b: Effects of the intervention packages on reporting being in school in the various follow-up rounds** 26](#_Toc182216317)

[**Figure S3: Percentage in school in each arm in follow-up rounds two to nine** 27](#_Toc182216318)

[**Table S17: Self-reported total exam score in best six subjects from grade nine exams** 27](#_Toc182216319)

[**Table S18: Effects of the intervention packages on reported ability to express own opinions and make own decisions** 28](#_Toc182216320)

[**Table S19: Pregnancy, marital status and timing of marriage and conception** 29](#_Toc182216321)

[Harms 29](#_Toc182216322)

[References 30](#_Toc182216323)

## Systematic literature search

### **Table S1: Search queries in each database**

| **Database** | **Search queries** | **Latest systematic review of database** |
| --- | --- | --- |
| Medline | (exp Pregnancy in Adolescence/ or ((Adolescent or teen or youth or youths or girls).mp. and (exp Pregnancy/ or pregnan*.mp. or birth*.mp. or childbirth.mp.))) and (((intervention or program or programme or trial or experiment or experimental) and random*).mp. or (exp Controlled Clinical Trial/ or exp Clinical Trial/ or exp Clinical Trial, Phase III/ or exp Randomized Controlled Trial/)) | *SSA:* Jan 26, 2022^1^  *LMICs & humanitarian settings:* December 31, 2018^2^ |
| EMBASE | (exp adolescent pregnancy/ or ((Adolescent or teen or youth or youths or girls).mp. and (exp pregnancy/ or pregnan*.mp. or birth*.mp. or childbirth.mp.))) and (((intervention or program or programme or trial or experiment or experimental) and random*).mp. or (exp controlled clinical trial/ or exp clinical trial/ or exp phase 3 clinical trial/ or exp randomized controlled trial/)) | *SSA:* Jan 26, 2022^1^  *LMICs & humanitarian settings:* December 31, 2018^2^ |
| Web of Science | ((Adolescent or teen* or youth or youths or girls) AND (pregnan* or birth* or childbirth)) AND (intervention or program or programme or trial or experiment or experimental) AND (random*) | *SSA:* Jan 26, 2022^1^  *LMICs & HICs:* July, 2017^3^ |
| Cochrane library | ((Adolescent or teen* or youth or youths or girls) AND (pregnan* or birth* or childbirth)) AND (intervention or program or programme or trial or experiment or experimental) AND (random*) | *SSA:* Jan 26, 2022^1^  *LMICs & HICs:* November 2015^4^ |
| ERIC | ((Adolescent or teen* or youth or youths or girls OR DE "Adolescents") AND (pregnan* or birth* or childbirth OR DE "Pregnancy")) AND ((intervention or program or programme or trial or experiment or experimental) AND (random*) OR DE "Randomized Controlled Trials" ) | *LMICs & HICs:* July, 2017^3^ |
| PsycInfo | (exp Adolescent Pregnancy/ or ((Adolescent or teen or youth or youths or girls).mp. and (exp Pregnancy/ or pregnan*.mp. or birth*.mp. or childbirth.mp.))) and (((intervention or program or programme or trial or experiment or experimental) and random*).mp. or (exp Clinical Trials/ or exp Randomized Controlled Trials/)) | *LMICs & humanitarian settings:* December 31, 2018^2^ |

HICs= high-income countries; LMICs= low- and middle-income countries; SSA= Sub-Saharan Africa

### **Table S2: Identified systematic reviews on the effectiveness of interventions on adolescent childbearing**

| **Author** | **LMICs** | **Population** | **Interventions** | **Venues** | **Databases** | **Period covered** |
| --- | --- | --- | --- | --- | --- | --- |
| Oringanje et al^4^ | LMICs and HICs | Male and female adolescents aged 10-19 years | Any interventions that aimed to increase knowledge and attitudes relating to risk of unintended pregnancies, promote delay in the initiation of sexual intercourse and encourage consistent use of birth control methods to reduce unintended pregnancies | School-based, community/home-based, clinic-based, and faith-based | The Cochrane Database of Systematic Reviews (CENTRAL), MEDLINE, EMBASE, LILACS, Social Science Citation Index and Science Citation Index, Dissertations Abstracts Online, the Gray Literature Network, HealthStar, PsycINFO, CINAHL, and POPLINE | Up to November 2015 |
| Hindin et al^5^ | LMICs | Young people 10-24 years | Prevention of early pregnancy or repeat pregnancy | Not restricted | PubMed, Embase, PsycInfo, Cinahl Plus, Popline, and the Cochrane Databases | 2000- November 2015 |
| Mason-Jones et al^6^ | LMICs and HICs | Adolescents 10-19 years attending school | Sexual and reproductive health programmes | School | MEDLINE, Embase, the Cochrane Databases, ClinicalTrials.gov, WHO International Clinical Trials Registry | 1990 to 7 April 2016 |
| Lopez et al^7^ | LMICs and HICs | Adolescents (majority 19 years or younger) | Interventions to improve contraceptive use | Middle or high school | PubMed, CENTRAL, ERIC, Web of Science, POPLINE, ClinicalTrials.gov, and ICTRP | Up to 6 June 2016 |
| Peterson et al^3^ | LMICs and HICs | Adolescents aged 10–19 years | Interventions addressing school environments or educational assets on adolescent sexual health | School | BiblioMap, CINAHL Plus, ERIC, IBSS, OpenGrey, ProQuest Dissertations & Theses, PsycINFO, Medline, and Web of Science Core Collection | 1990-July 2017 |
| Desrosiers et al^2^ | LMIC and humanitarian settings | Young people | SRH interventions (educational, psychosocial, prevention, community-based, psychoeducational, empowerment, mental health, psychological, counseling, family-based, and training programs) | Not restricted | Pubmed, Psycinfo, Medline, and Embase | 2000-2018 |
| Estrada et al^8^ | LMICs and HICs | Adolescents aged 10-19 years | Prevention of adolescent pregnancy | Not restricted | PubMed, and 3ie database | 2005-March 2019 |
| Ivanova et al^9^ | LMICs | Adolescents and youth aged 10–24 years old | Sexuality education | School or community | PubMed, and Web of Science | Up to Aug 2019 |
| Shangase et al^10^ | SSA | Adolescents aged 9–19 years | Interventions promoting positive SRH to reduce sexual risk behaviors and risk of curable STIs and unintended pregnancies | School | PubMed, PubMed Central, the Cochrane Databases, and ClinicalTrials.gov | Up to December 2019 |
| Feyissa et al^1^ | SSA | Adolescent girls | Interventions to reduce teen pregnancy and child marriage | Not restricted | PubMed, EMBASE, CINAHL, Web of Science, Science Direct, CENTRAL, and 3ie database | Up to 26 January 2022 |

HICs= high-income countries; LMICs= low- and middle-income countries; SSA= Sub-Saharan Africa; SRH= sexual and reproductive health; STIs=sexually transmitted infections

### **Table S3: Identified relevant randomised controlled trials evaluating the effectiveness of interventions on adolescent childbearing in LMICs**

| **Author** | **Setting** | **Population** | **Interventions** | **Intervention duration** |
| --- | --- | --- | --- | --- |
| Austrian et al^11^ | Zambia | Never married girls aged 10-19 years | Life skills and financial education | 2 years |
|  |  |  | Life skills, financial education, and health vouchers |  |
| Austrian et al^12^ | Kenya | Girls aged 11-14 years | CCT (to household and school, conditional on enrollment and attendance) | 2 years |
|  |  |  | CCT and health/life skills |  |
|  |  |  | CCT, health/life skills, financial literacy, savings curriculum, and annual savings incentive |  |
| Baird et al^13,14^ | Malawi | Never-married females aged 13-22 years | CCT (to girls and household, conditional on school attendance) | 2 years |
|  |  |  | UCT (to girls and household) |  |
| Bandiera et al^15^ | Uganda | Girls aged 14-20 years | Life skills (including SRHE), vocational skills, financial literacy, and in-kind support | 2 years |
| Berge et al^16^ | Tanzania | Girls at the end of secondary education | CSE and booklet | 8 weeks |
|  |  |  | Economic empowerment (how to start business, building self-confidence) |  |
|  |  |  | CSE and economic empowerment |  |
| Buchmann et al^17^ | Bangladesh | Girls aged 15-17 years | Empowerment (safe spaces, education support, life skills, SRHE) | 6 months |
|  |  |  | Conditional cooking oil (until age 18, conditional on remaining unmarried) | 2 years |
|  |  |  | Empowerment and conditional cooking oil | 2 years |
| Buehren et al^18^ | Tanzania | Girls (no age restrictions, median age <16 years) | Life skills (including SRHE), vocational skills, and financial literacy | 2 years |
|  |  |  | Life skills, vocational skills, financial literacy and in-kind support |  |
| Burke et al^19^ | South Africa | Orphans and vulnerable children aged 14-17 years | HIV prevention/CSE | 4 months |
|  |  |  | Economic strengthening (budgeting, saving, earning) | 4 months |
|  |  |  | HIV prevention/CSE and economic strengthening | 8 months |
| Cabezon et al^20^ | Chile | Pupils first year high school | Abstinence focused SRHE | one school year |
| Cho et al^21^ | Kenya | Orphaned boys and girls in grades 7-8 | School uniform, payment tuition, nurse visits | 36 months |
| Cowan et al^22,23^ | Zimbabwe | 18-22 years old (boys and girls) | In-school CSE, training in YFHS, community meetings | 4 years |
| Dake et al^24^ | Zambia (Z) and Malawi (M) | Girls aged 14-21 years (M: ultra-poor and labour-constrained households; Z: female of elderly-headed households keeping orphans and households with disabled members) | UCT (to household) | 3 years (Z) and 2·5 years (M) |
| Duflo et al^25^ | Kenya | Pupils grade 6 | Abstinence-only SRHE | 3 years |
|  |  |  | School uniform |  |
|  |  |  | School uniform and abstinence-only SRHE |  |
| Dunbar et al^26^ | Zimbabwe | Female orphans aged 16-19 years, out of school, not currently pregnant, HIV negative | Vocational training, financial literacy, microgrants, social support | 6 months |
| Dupas et al^27^ /Duflo et al^28^ | Ghana | Youths who had taken junior high school exit exam and had not enrolled in senior high school due to financial constraints | Scholarship for fees | 4 years |
| Hallfors et al^29,30^ | Zimbabwe | Orphan girls in grade 6 | Payment school fees, uniforms, school-based helper | 5 years |
| Handa et al^31^ | Kenya | Households with orphans and vulnerable children under 18 | UCTs (to household) | 4 years |
| Jewkes et al^32^ | South Africa | Girls and boys aged 15-26 years | CSE | 6-8 weeks |
| Kangwana et al^33^ | Kenya | Girls aged 11-14 years | CCT (to household and school, conditional on school enrollment and attendance) | 2 years |
|  |  |  | CCT and health/life skills | 2 years |
|  |  |  | CCT, health/life skills, financial literacy and savings curriculum, and annual savings incentive | 2 years |
| Mbizvo et al^34^ | Zambia | Pupils (age and grade not specified) | Monthly school health fairs with SRH services, and referral slips to health facilities | Not specified |
|  |  |  | Encouraged to access SRH services at health facilities where staff trained in YFHS |  |
| Özler et al^35^ | Liberia | Girls aged 13-14 years | GE: Life skills, caregiver discussion groups, individual savings start-up for girls, capacity building for health providers | 8 months |
|  |  |  | GE+: Life skills, caregiver discussion groups, individual savings, capacity building for health providers, and CCTs (for parents, conditional on girls´ attendance of life skills training) |  |
| Pettifor et al^36^ | South Africa | Girls aged 13-20 years in grades 8-11, not married or pregnant | Monthly cash (to girls and parents, conditional on school attendance) | 3 years |
| Pike et al^37^ | South Africa | Non-pregnant girls in grades 8-10, aged 13-17 years | CSE | 6 months |
| Ross et al^38,39^ | Tanzania | Pupils aged 14 years in grades 5-7 | Teacher led in-school CSE, annual health weeks, condom promotion and distribution | 1-3 years |
| Taylor et al^40^ | South Africa | Pupils in grade 8 | CSE (weekly sessions) | 12 weeks |
| Waidler et al^41^ | Tanzania | Girls aged 14-19 years | Livelihood and life skills training, grant (conditional on making education or business plan), adolescent friendly health service strengthening | 17 months |
| Zulaika et al^42,43^ | Kenya | Girls in forms 2 and 3 (aged 16-18 years) | CCT (to girls, conditional on school attendance) | Until completion of secondary school (median 2·5 years) |

CSE= Comprehensive sexuality education; CCT= conditional cash transfer; UCT= unconditional cash transfer; GE= Gender empowerment; SRHE= sexual and reproductive health education; YFHS= youth friendly health services

## Protocol deviations

We had initially planned that individual schools in which <85% of the girls assented and their guardians consented would not be included in the trial. However, to achieve the desired sample size of 157 clusters, we ended up including one school where 18% of the eligible participants did not assent.

In the protocol we had stated that the interviewers in the final follow-up round would be unaware of the intervention status of the respondents.^44^ Although we avoided specifying to the interviewers which arm each of the schools belonged to, many of the interviewers became aware of which arm a school belonged to because the participants often talked about the support they received/previously had received. Because of the sensitive nature of many of the questions in the follow-up interviews, we concluded that it was better to use interviewers the participants were familiar with and had developed trust in than to recruit new interviewers in the final interview round. To keep the research assistants as neutral as possible, we avoided mentioning the main study hypotheses to them and rather emphasised the importance of capturing all participants´ experiences and perspectives in each follow-up round.

We indicated that we would use Cox regression in the analysis of birth and pregnancy outcomes. Since the assumption of proportional hazards did not hold for the first year after randomisation, a parametric model was employed instead of Cox regression (we selected the Weibull model based on the distribution of the overall incidence rate according to a Kaplan-Meier curve).

To minimize recall bias, we had described in the protocol that we would measure *“Birth within eight months of the end of the intervention period”* using information collected 44 months after recruitment (corresponding to the seventh follow-up round), i.e. soon after the measurement point, and information collected up to 56 months after recruitment (including final follow-up rounds) would be used for validation.^44^ However, since we succeeded in interviewing a higher proportion of the participants in the final follow-up than in the seventh round, the latter version of the outcome was less affected by loss-to-follow-up. We thus used this version as the primary outcome.

## Trial design

The Research Initiative to Support the Empowerment of girls (RISE) was a parallel superiority cluster randomised controlled trial.

## Recruitment and randomisation

The trial was conducted in the following districts: Kalomo, Choma, Pemba, Monze, Mazabuka, Chikankata, Chisamba, Chibombo, Kabwe, Kapiri Mposhi, Luano and Mkushi. The districts were selected because they could be reached within five hours from Lusaka and because they (except for Kabwe) in the 2010 census had slightly higher percentages (23.8-27.9%) of 17–18-year-olds who had given birth than the national average (20.7%). Some rural schools in Kabwe (which is a predominantly urban district, located between Kapiri Mposhi and Chisamba districts) were invited to participate since a few more schools were needed and we assumed that the catchment areas of the rural schools would have higher adolescent childbearing rates than the overall estimates for the district indicated (the overall percentage of girls aged 17-18 years who had given birth in Kabwe was 13.8%, i.e. lower than the national average). All the selected schools were at least eight kilometres apart, had some mobile phone coverage and were accessible by car throughout the year.

Chiefs, headmen, religious and informal leaders, headmasters and parent-teacher association (PTA) members were oriented by trial staff and asked to support the trial activities before recruitment was initiated. Local radio was also used to sensitise communities about the trial. The information provided emphasised that the aim was to test different ways of enhancing the opportunities of girls and their communities. They were told that all the participants would benefit, but the components of the support packages would vary between schools. Some schools would receive material support only, some would receive material and economic support, and for some schools the support package would consist of material and economic support and community dialogue. They were also informed that a lottery would determine which schools would receive which package. In addition, information about follow-up interviews was provided.

In total 164 schools with surrounding communities were invited to participate in the trial. All registered female pupils in grade seven were eligible. In one school, community leaders did not give permission for their school to take part. In another six schools, less than 80% of the grade seven girls and their guardians assented/consented during recruitment, and these schools were not randomised. The recruitment of participants was done by research assistants under the guidance of a supervisor.

Before each randomisation ceremony, 1000 numbered allocations, stratified by district, were computer-generated by an independent scientist. Headteachers, PTA chairpersons, chiefs and representatives from the District Educational Board Secretary offices were invited (and received transport reimbursements) to attend the randomisation ceremonies. The ceremonies were also open to the general public, and announcements about the ceremonies were made via the local radio channels. At each ceremony, three chiefs drew numbered tickets from a box, creating a three-digit number corresponding to a specific allocation of schools.

## Intervention details

The interventions were developed based on a systematic literature review, formative research,^45^ and consultations with the Ministries of Health and General Education. A short pilot study was also conducted.^46^ A cluster design was chosen because the interventions aimed to change community norms, and it was more practical to offer comprehensive sexuality education to all the pupils in a class than to limit it to individuals.

The writing materials offered to the participants in all three study arms comprised exercise books, pens and pencils worth approximately 16 USD in total. These were distributed in September 2016, January 2017 and January 2018.

#### Economic support

Secondary school fees in the trial schools varied between ZMW 210 and ZMW 1950 in 2017, and some participants enrolled in other secondary schools with fees up to ZMW 9900. As part of the economic support we paid the tuition part of the fees (on average 88% of the total), up to a maximum of ZMW 1500 per year. Payments of school fees were made directly to the school bank accounts.

The cash transfers were disbursed by committees consisting of a teacher and two parents from the Parent-Teacher Association. At least two of the cash transfer committee members should be present during disbursements to witness that the right persons received the cash and signed for it. The annual cash amount (ZMW 710 in total for girls and guardians) was similar to the amounts provided to households in the government´s cash transfer programmes.^24,47^

#### CSE and Community dialogue

The youth club manual was based on the “Tuko Pamoja. Adolescent Reproductive Health and Life Skills Curriculum” developed by the Kenya Adolescent Reproductive Health Program, Program for Appropriate Technology in Health (PATH), and the Population Council.^48^ This curriculum includes the following topics: gender roles and stereotypes, peer pressure, prevention of unwanted pregnancy (including modern contraceptives), and sexually transmitted infections. We added sessions on the value of education, school re-entry, early marriage, and the right to access sexual and reproductive health (SRH) information and services. The most common activities were role plays, group discussions, and plenary discussions, and the aim was to build life skills such as resisting peer pressure, assertiveness, setting goals, making rational decisions, solving conflicts, and communicating about feelings and relationships. The manual was reviewed and approved by the Ministry of General Education (MoGE), and can be found [here](https://drive.google.com/drive/folders/1dMLFUMehvP0ZSlF-t-dG2jy8GsJkg_OI?usp=sharing). The meetings were held in a classroom after school hours.

The community dialogue meetings manual included a guide on how to facilitate a participatory workshop and described in detail activities for each session. The topics included early childbearing, the value of education, communication with adolescents about SRH, children’s rights and responsibilities, sexual abuse, and gender-based violence. Most of the meetings included a role play to start discussions. These meetings were also held in classrooms or near the school, depending on whether a classroom was available.

Before the intervention period started, one teacher and one community health worker (CHW; with six weeks basic training) or community health assistant (CHA; with one year training programme) from each cluster in the combined intervention arm were trained for 5 days on the use of the manuals, facilitation techniques, and community mobilization. A 3-day refresher training was held after one year where emphasis was given to sharing experiences and value clarification. The teacher and CHA/CHW facilitated the youth club and community meetings together. At the end of each monitoring visit by project staff, the facilitators were given feedback on how well they facilitated the session and how they could more actively engage the pupils in the topics covered.

For each youth club, two young (<20 years) unmarried women from the local community were selected as peer mobilisers with the task of encouraging girls and boys to come to youth club meetings. They were trained for 3 days in communication skills, community mobilization, and sexual and reproductive rights.

A half day orientation meeting was held once (in November 2016) by Ministry of Health (MoH) staff for health care workers in the catchment area of the combined intervention schools about the importance of providing youth friendly health services. There was no monitoring of the health services provided.

### **Table S4: Timing of interview rounds**

| **Interview round** | **Timing** |
| --- | --- |
| Baseline | March-July 2016 |
| First follow-up | October 2016-March 2017 |
| Second follow-up | May-November 2017 |
| Third follow-up | September 2017-March 2018 |
| Fourth follow-up | January-September 2018 |
| Fifth follow-up | August 2018-February 2019 |
| Sixth follow-up | January -August 2019 |
| Seventh follow-up | August 2019-January 2020 |
| Eighth follow-up | January-July 2020 |
| Ninth follow-up | July-December 2020 |

## Detailed information about the definition of outcomes

### **Table S5: Outcomes included in this paper**

|  | **Measurement tools** | **Months from recruitment to the last measurement** |
| --- | --- | --- |
| **Primary outcomes** |  |  |
| Birth within eight months of the end of the intervention period | Follow-up questionnaires | 40-45 (seventh follow-up round)  52-57 months (ninth follow-up round) |
| Birth before the 18th birthday | Follow-up questionnaires | 52-57 (ninth follow-up round) |
| Sitting for grade nine exams | Follow-up questionnaires  For validation: Exam registers for 2018-2020 | 52-57 (ninth follow-up round) |
| **Secondary outcomes** |  |  |
| Pregnancy before the 18th birthday | Follow-up questionnaires | 52-57 (ninth follow-up round) |
| Perceived community norms regarding adolescent pregnancy among girls | Follow-up questionnaires | 29-35 (fifth follow-up round) |
| Perceived community norms regarding education among girls | Follow-up questionnaires | 29-35 (fifth follow-up round) |

Considering that information on adolescent childbearing is sensitive, we anticipated that some participants would not always be completely truthful when responding to questions about this. For example, having to respond to questions from a new interviewer they had not met before could make them hold back information on such issues. We also anticipated that the participants would feel more embarrassed when responding to sensitive questions in the first interview rounds than in later rounds when they became more accustomed to such questions. In the last two years of the trial, the interviewer team was very stable, and 80-90% of the participants were interviewed in the last rounds by someone they had met several times before. We expected that as the participants became increasingly familiar with the interviewers and the questions and as they became older – implying they had reached an age when it was more acceptable to have a child - they would be more likely to be truthful in their responses to sensitive questions. Whether a participant had been *pregnant or given birth* were thus defined based on information from the *last* available interview or interaction taking place before the defined measurement point. When the research team was informed that a participant who died had been pregnant or had a child, this information was utilized.

In the first interview rounds the interviewers experienced that it was not possible to rely only on the question on date of birth to verify that the right respondent had been identified because participants were not consistent in which dates they gave. Thus we anticipated that recall problems would cause inconsistencies between follow-up rounds in the dates reported for the timing of deliveries and the end of pregnancies. Therefore, we used information on *timing* of birth/end of pregnancy and *duration* of a pregnancy from the *first* interview it was reported to reduce the risk of recall bias.

Giving birth was defined as responding “yes” to at least one of the following questions: “Have you ever given birth?”, “Do you have your own child?”, “Have you ever had a live birth?” or “Have you ever had a stillbirth?”, or responding “live birth” or “stillbirth” to “How did your last pregnancy end?”. We did not count reported births if the pregnancy duration was given as five months or less, but included pregnancies with reported duration of six months or more as births.

A pregnancy was defined as reporting a birth (see above) or responding “yes” to either “When you skipped your period was it because you were pregnant?”, “Have you ever had a miscarriage/spontaneous abortion?”, “Have you ever been pregnant?”, or “Are you currently pregnant?”.

Pregnancies conceived before the randomisation (15th July 2016) were excluded from the analysis of the incidence of births and pregnancies. The duration of the pregnancy was subtracted from the reported time of birth/end of pregnancy to arrive at the estimated conception date. For those who responded “no” to “Was the baby born more than one month before the due date?”, we assumed that the pregnancy duration was nine months. Since follow-up rounds 1-5 did not include questions about the duration of previous pregnancies before the last birth, we assumed that the duration of such previous pregnancies was 9.

In the ACASI part of the interview, participants who responded “yes” to “Have you ever given birth?” or “Have you ever had a stillbirth?”, were only asked to give the year when their last pregnancy ended (not the exact date). In cases where only the year of the end of the previous pregnancy was available, the date of birth was set at the midpoint of the year, i.e. 1^st^ July, except when a pregnancy was reported to have ended within the same year that the interview was done. In the latter case, the date was assumed to be the midpoint between 1^st^ January and the date of the interview. For participants who did not have information on the date or year a birth/stillbirth occurred in any of the follow-up rounds, we assumed the birth/stillbirth took place on the day when the girl first reported it.

For the outcome *“Birth within eight months of the end of the intervention period”,* 15^th^ July 2019 was set as the end point since the intervention period ended on the 15^th^ November 2018. (The underlying assumption here was that pregnancies that ended within eight months had passed since the intervention ended, were conceived during the intervention period.) For the outcomes *“birth before the 18^th^ birthday*” and *“pregnancy before the 18^th^ birthday*”, we used the first birth and conception, respectively, reported before the 18^th^ birthday. Participants who had already reached their 18th birthday at baseline, were excluded from the analyses of these two outcomes.

The outcome *“sat for grade nine exams”* was measured as responding “yes” to the question “Did you sit for any grade nine exams last year?” in the 2019 or 2020 interview rounds or “Have you sat for any grade nine exam this year?” (in the final interview in 2020). We had planned to use information from the exam registries of the District Educational Board Secretary (DEBS) offices to validate self-reported information on grade nine exams. Unfortunately, we only obtained permission to access exam information from schools in the study districts, and as a result we did not have official exam information on those who wrote exams in other districts. In addition, we discovered that some participants were registered under different surnames or first names in the exam register than they had used when they were recruited into the trial, and thus some may have been missed when extracting information from the registries. As a result, we were only able to confirm that 75% of those who reported sitting for a grade nine exam, did so. For the validation analysis, those who were not listed in the exam registries were assumed not to have written any grade nine exam. We calculated the proportion of all participants who had attended at least one exam, including both those who passed and failed.

We calculated the mean self-reported total number of marks from the best six subjects, including English Language, in the grade nine examinations, using two different denominators: those who reported a total score, and all participants (to take into account that the proportions that sat for the exams also differed between the arms). For those who reported sitting for the grade nine exams more than once, we used the first score reported.

Perceived community norms regarding adolescent pregnancy and education were measured with two and three questions, respectively (see Table S6). The responses were recoded such that higher values indicated accepting pregnancy and being supportive of girls completing basic education, and then the responses were summed up in composite variables and dichotomised - with the median value as the cut-off - for the two types of norms.

### **Table S6: Questions used to measure each of the perceived community norm outcomes and description of how the responses were scored**

| **Outcome** | **Questions** | **Scoring of responses** | **Missing responses** |
| --- | --- | --- | --- |
| Perceived community norms regarding adolescent pregnancy outside marriage | “Imagine that there are two young girls of the same age and both of them are unmarried; one has a child and the other does not. How do you believe your parent would treat these two girls?” | *“They would treat the girl with a child with more respect”* = 2; “*They would treat the girl with no child with more respect”* = 0; “*They would treat them the same*“ = 1 | Refused to respond: 6 |
|  | “What do your parents or guardians think if a young girl who has reached puberty, has completed grade nine and is out-of-school, gets pregnant?” | *“OK”* = 1; “*not OK”* = 0” | Refused to respond: 7 |
| Perceived community norms regarding education for girls | “How important does your father/male guardian think it is that you complete grade nine?” | *“Very important”* = 2; “*Not important”* = 0; “*A little bit important”* = 1; | Refused to respond: 4; No father or male guardian: 357 |
|  | “How important does your mother/female guardian think it is that you complete grade nine?” | *“Very important”* = 2; “*Not important”* = 0; “*A little bit important”* = 1 | No mother or female guardian: 61 |
|  | “Do your parents/guardians think that it is more important for boys or girls to complete grade nine?” | *“They believe it is more important for girls”* = 2; *“They believe it is more important for boys”* = 0; *“They believe it is equally important for boys and girls”* = 1 | Refused to respond: 18 |

A number of other secondary outcomes were also measured (see list in the protocol paper),^44^ but these will be reported in other forthcoming publications.

## Data management

Data from interviews were captured electronically using tablets. The forms had inbuilt check- and skip-rules to minimize data entry errors. Each participant was given a unique identifier, and only the Data manager(s) and Principal Investigator had access to personal identifiers. Names and telephone numbers were stored separately from sensitive data. All data was saved on password protected computers and tablets and a safe server owned by the University of Bergen.

## Details about the statistical analysis

Girls were the units of analysis. Clustered robust standard errors were used to take account of the design effect.

We calculated frequencies and percentages for categorical variables and means and standard deviations for continuous variables.

Time-on-study was used as the time scale for births within eight months of the end of the intervention period, and adjustment was made for the age at entry. Age was the time scale, accounting for age-truncation, for births and conceptions before the 18th birthday. For the incidence estimations, participants who withdrew, died or were lost to follow-up for other reasons before experiencing the outcome, were right-censored at the time when we last received information about their status. For those who were pregnant at randomisation, we counted their time at risk of a new conception/birth from the day the pregnancy ended/day of delivery.

The number needed to benefit (NNTB) and the number needed to harm (NNTH) were calculated as 1/risk difference.

Baseline confounders were defined as variables where there was a relative difference of ≥5% and an absolute difference of ≥2·5% points in the proportions with the most common predefined response if they were associated with the outcome (i.e. if the inclusion of the variable changed the effect estimate with ≥5% or substantially increased its statistical precision).

*Sensitivity analyses*

1. To explore the impact of ***loss to follow-up*** we conducted three types of sensitivity analyses*:*
2. *Using information on marital status:* Considering that several of the participants withdrew from the trial after getting married and that the probability was high that girls who married became pregnant within the first year after entering marriage, we made a composite outcome where we combined information on marriage and childbearing. We assumed that those who were lost to follow-up soon after or around the time they got married, gave birth nine months after entering marriage. If they were interviewed after becoming married but did not report being pregnant and then later were lost to follow-up, we assumed they gave birth six months after the last interview. Unmarried individuals who were lost to follow-up were assumed not to have given birth within the period of interest.
3. *Using proxy information*: Information collected from family members, teachers, friends, and neighbours, about births of girls who were lost to follow-up for other reasons than withdrawal or death was combined with the interview data. We compared the proportion of girls who had given birth with and without using this proxy source of information.
4. *Using inverse probability weighting*: This analysis was not included in the analysis plan but added posthoc. Information from those who did not participate in the last follow-up round was excluded and cases with complete follow-up information were weighted by the inverse probability of being a complete case. The following baseline variables were assumed to predict the probability of being censored (i.e. not interviewed in the final follow-up round): study arm, age at randomisation (categorized as 10-12·99 years, 13-15·99 years. 16-17·99 years and 18 and above), highest completed educational level of guardian or head of household (categorized as primary or lower and secondary or higher), and household wealth (categorized into tertiles).
5. *Sensitivity analyses considering* ***induction time****:* In the analysis plan we had not specified that we would take induction time into account in the analyses of births. To check whether this led to an underestimation of the effects, we did a posthoc sensitivity analysis where we assumed six months (H_ind6_) or 12 months (H_ind12_) induction time after the start of the period at risk of a pregnancy (i.e. after the date of randomisation or the end of a pregnancy conceived before the randomisation). The rationale for H_ind6_ was that we considered six months to be the minimum duration of a pregnancy for a fetus to be viable, and the rationale for H_ind12_ was that the intervention started two to three months after the randomisation, and a pregnancy duration is typically nine months. For H_ind12_, those who were pregnant at the time of randomisaton and gave birth more than three months after the randomisation (i.e. after the start of the intervention period), were assumed to have an induction time of nine months after the previous pregnancy ended.

## Ethical aspects

Permissions were obtained from the Zambia MoGE, MoH and the DEBS offices in the study districts to conduct the trial in schools and to engage teachers and CHAs/CHWs in the implementation of the interventions. All interviews were conducted in privacy, and strict confidentiality was kept. A Data Monitoring Committee reviewed the reported deaths and hospitalizations related to pregnancy at least once per year to ensure that the trial did not lead to unintended effects on unsafe abortions.

## Role of the funding source

The Research Council of Norway, the University of Bergen and the Swedish International Development Cooperation Agency (SIDA) had no role in the study design, data collection, data analysis, data interpretation or writing of the paper.

## Supplementary findings

### **Table S7: Number and percentages of participants interviewed in each of the follow-up rounds**

|  | **Control** | **Economic** | **Combined** | **Total** |
| --- | --- | --- | --- | --- |
| Total number of participants at the time of the randomisation | 999 | 2004 | 1919 | 4922 |
| First follow-up | 969 (97·0%) | 1974 (98·5%) | 1876 (97·8%) | 4819 (97·9%) |
| Second follow-up | 926 (92·7%) | 1901 (94·9%) | 1853 (96·6%) | 4680 (95·1%) |
| Third follow-up | 913 (91·4%) | 1863 (93·0%) | 1830 (95·4%) | 4606 (93·6%) |
| Fourth follow-up | 832 (83·3%) | 1794 (89·5%) | 1717 (89·5%) | 4343 (88·2%) |
| Fifth follow-up | 855 (85·6%) | 1801 (89·9%) | 1742 (90·8%) | 4398 (89·4%) |
| Sixth follow-up | 904 (90·5%) | 1872 (93·4%) | 1796 (93·6%) | 4572 (92·9%) |
| Seventh follow-up | 902 (90·3%) | 1866 (93·1%) | 1781 (92·8%) | 4549 (92·4%) |
| Eighth follow-up | 897 (89·8%) | 1855 (92·6%) | 1754 (91·4%) | 4506 (91·5%) |
| Ninth follow-up | 936 (93·7%) | 1927 (96·2%) | 1828 (95·3%) | 4691 (95·3%) |

### **Intervention coverage**

The signed cash disbursement lists were reviewed by project staff every month. To strengthen accountability further, participants in the intervention arms were also asked in every follow-up contact about exposure to the intervention components, including how much money they had received. As many as 1997 of 2004 (99·7%) in the economic arm and 1907 of 1919 (99·4%) in the combined intervention arm signed for the monthly cash at least once. As many as 87% in the two intervention arms signed for the cash more than 20 of the 27 intervention months, and 50% in the economic and 62% in the combined arm signed for the cash in all the 27 intervention months. The mean and median number of months girls signed for cash were 24·4 and 26 in the economic arm and 24·6 and 27 in the combined arm, respectively. School fees were paid for 1793 (89%) and 1755 (91%) in 2017 and 1531 (76%) and 1559 (81%) in 2018 in the economic and combined arms, respectively.

Fifty percent of those in the combined arm who were interviewed in all the follow-up rounds during the intervention period, indicated attending at least half of the 36 meetings in the youth clubs, 6% attended at least 27 (≥75%) meetings and 85% attended at least 9 (≥25%) meetings. Among guardians who were interviewed in 2018, the average reported number of meetings attended in the previous six months was 1.5 in the control, 2 in the economic arm and 3.2 in the combined arm. As many as 19% in the control, 30% in the economic and 53% in the combined arms reported attending three or more RISE meetings in the six-month period (Table S8). The meetings they referred to probably included the annual parent meetings in all three arms and community dialogue meetings in the combined arm. The relatively high proportion of guardians in the control and economic arms who reported attending multiple RISE meetings could either reflect contamination – with guardians attending meetings meant for those in the combined arm - or recall bias. Since the number of adults who attended community dialogue meetings was usually less than 50, it seems more likely that many guardians referred to the number of meetings they had attended over a much longer period than six months. Such recall problems may have occurred in all three arms, and the difference of 1.2-1.7 between the averages in the combined versus the economic and control arms may reflect that guardians in the combined arm typically attended approximately half of the three community dialogue meetings organized in the preceding six months.

We are not convinced that the brief orientation about youth friendly health services for local health workers made much of a difference as we never heard any of the health workers refer to the orientation afterwards. Another element that appeared to be superfluous was the involvement of two peer mobilisers per youth club. We found that youth club attendance appeared to be unaffected by the fact that a high number of the peer mobilisers dropped out within the first year.

### **Table S8: Guardians’ attendance of RISE meetings in previous six months according to parent interviews in 2018**

| **Number of RISE meetings attended** | **Control (N=730)**  n (%) | **Economic (N=1535)**  n (%) | **Combined (N=1489)**  n (%) |
| --- | --- | --- | --- |
| 0 | 212 (29·0%) | 274 (17·8%) | 119 (8·0%) |
| 1 | 125 (17·1%) | 230 (15·0%) | 84 (5·6%) |
| 2 | 156 (21·4%) | 356 (23·2%) | 215 (14·4%) |
| 3 | 88 (12·0%) | 279 (18·2%) | 268 (18·0%) |
| 4 | 34 (4·7%) | 111 (7·2%) | 243 (16·3%) |
| 5 | 9 (1·2%) | 41 (2·7%) | 139 (9·3%) |
| 6 | 9 (1·2%) | 22 (1·4%) | 130 (8·7%) |
| 7 | 0 (0·0%) | 2 (0·2%) | 16 (1·1%) |
| Dont’ know | 97 (13·3%) | 219 (14·3%) | 275 (18·5%) |

The denominator is the number of guardians interviewed in each arm.

### **Figure S1: Cumulative incidence of giving birth within eight months of end of intervention period**

### **Figure S2: Cumulative incidence of giving birth before 18th birthday**

### **Table S9: Effects of the intervention packages on giving birth within eight months of the end of the intervention period (using data collected up to 40-45 months after the randomisation/8-14 months after the end of the intervention period)**

|  | **Control** | **Economic** | **Combined** |  |  | **Economic vs control** | **Combined vs control** | **Combined vs economic** | **ICC (95% CI)** |
| --- | --- | --- | --- | --- | --- | --- | --- | --- | --- |
| n/N (%) | 257/988 (26%) | 468/1997 (23%) | 452/1901 (24%) |  | Age adj. HR (95% CI) | 0·90 (0·75, 1·07) | 0·89 (0·75, 1·05) | 0·99 (0·85, 1·15) | 0·028 (0·015, 0·051) |
|  |  |  |  |  | p-value | 0·224 | 0·170 | 0·888 |  |
| Rate per person year | 0·099 | 0·087 | 0·087 |  | RD | -0·025(-0·065, 0·016) | -0·023 (-0·062, 0·0152) | 0·0014 (-0·031, 0·034) |  |
|  |  |  |  |  | NNTB | 40.4 (NNTH 64.3 to ∞ to NNTB 15.4) | 42.8 (NNTH 65.0 to ∞ to NNTB 16.1) | NNTH 717.1 (NNTH 29.6 to ∞ to NNTB 32.2) |  |

HR= hazard ratio; ICC=intracluster correlation; NNTB= number needed to benefit; NNTH= number needed to harm; RD=risk difference

### **Inconsistencies in reporting of births**

We measured *birth within eight months of the end of the intervention period* at two points in time: 8-14 and 20-25 months after the interventions ended (40-45 and 52-57 months after recruitment). When limiting the outcome definition to information collected up to 8-14 months after the interventions ended, the incidence was somewhat lower and the effect of the combined support appeared smaller (HR 0·89, 95% CI 0·75–1·05, see Table S9) than when using information from the final follow-up (HR 0·82; 95% CI 0·69-0·97, Table 2). There was no important difference in the estimated effects of economic support using data collected up to the two measurement points (HR 0·90 versus 0·89, Table 2 and S8). The discrepancies between the estimates from the two measurement points for the combined support was partly due to inconsistencies in the self-reporting of births (e.g. 1·6 % of those who reported giving birth in the seventh follow-up, denied this in the final follow-up). Nevertheless, the main reason for the differences in the estimates was that a higher percentage was interviewed in the final follow-up round. Several participants who had been difficult to track because they had married and moved elsewhere were captured in the final follow-up, and a very high proportion of them had given birth. Thus, we suspect the estimates from the final follow-up to be the least affected by bias.

We also checked whether any of those who reported not giving birth in the final follow-up had ever reported births conceived after randomisation in earlier interview rounds and found that this was the case for 38 out of 2707 (1·4%). If we assume that these 38 had also given birth, this would not give important changes in the effect estimates (HR 0·88 (95% CI 0·73-1·04) versus 0·89 (95% CI 0·75-1·06) for economic support and HR 0·82 (95% CI 0·69-0·98) versus 0·82 (95% CI 0·69-0·97) for combined support).

### **Table S10: Effects of the intervention packages on birth and pregnancy outcomes using Cox regression (as mentioned in the data analysis plan)**

|  | **Economic vs control** | **Combined vs control** | **Combined vs economic** |
| --- | --- | --- | --- |
| *Birth within eight months of the end of the intervention period (incl, data collected up to 52-57 months after recruitment)* |  |  |  |
| Age-adjusted HR (95% CI) | 0·89 (0·75, 1·06) | 0·82 (0·69, 0·97) | 0·91 (0·79, 1·06) |
| p-value | 0·204 | 0·023 | 0·241 |
|  |  |  |  |
| *Birth within eight months of the end of the intervention period (using data collected up to 40-45 months after recruitment)* |  |  |  |
| Age adjusted HR (95% CI) | 0·90 (0·76, 1·07) | 0·89 (0·75, 1·05) | 0·99 (0·85, 1·15) |
| p-value | 0·229 | 0·175 | 0·892 |
|  |  |  |  |
| *Birth before the 18^th^ birthday* |  |  |  |
| HR (95% CI) | 0·92 (0·77, 1·12) | 0·89 (0·73, 1·08) | 0·96 (0·82, 1·14) |
| p-value | 0·409 | 0·233 | 0·652 |
|  |  |  |  |
| *Pregnancy before the 18^th^ birthday* |  |  |  |
| HR (95% CI) | 0·88 (0·75, 1·05) | 0·82 (0·69, 0·97) | 0·92 (0·80, 1·07) |
| p-value | 0·158 | 0·021 | 0·278 |

**Table S11a. Sensitivity analysis of the effects of the intervention packages taking six months induction time into account**

|  | **Control** | **Economic** | **Combined** |  |  | **Economic vs control** | **Combined vs control** | **Combined vs economic** |
| --- | --- | --- | --- | --- | --- | --- | --- | --- |
| *Birth within eight months of the end of the intervention period (incl. data collected up to 56 months)* |  |  |  |  |  |  |  |  |
| n/N (%) | 282/980 (29%) | 520/1990 (26%) | 466/1892 (25%) |  | Age adjusted HR (95% CI) | 0·90 (0·75, 1·07) | 0·82 (0·69, 0·97) | 0·91 (0·79, 1·06) |
| Rate per person year | 0·13 | 0·12 | 0·11 |  | p-value | 0·217 | 0·024 | 0·235 |
|  |  |  |  |  |  |  |  |  |
| *Birth before the 18th birthday* |  |  |  |  |  |  |  |  |
| n/N (%) | 283/972 (29%) | 549/1955 (28%) | 528/1870 (28%) |  | HR (95% CI) | 0·92 (0·76, 1·11) | 0·89 (0·73, 1·08) | 0·96 (0·82, 1·14) |
| Rate per person year | 0·12 | 0·11 | 0·11 |  | p-value | 0·403 | 0·231 | 0·657 |

**Table S11b: Sensitivity analysis of the effects of the intervention packages taking 12* months induction time into account**

|  | **Control** | **Economic** | **Combined** |  |  | **Economic vs control** | **Combined vs control** | **Combined vs economic** |
| --- | --- | --- | --- | --- | --- | --- | --- | --- |
| *Birth within eight months of the end of the intervention period (incl data collected up to 52-57 months after recruitment)* |  |  |  |  |  |  |  |  |
| n/N (%) | 259/980 (26%) | 483/1987 (24%) | 435/1889 (23%) |  | Age adjusted HR (95% CI) | 0·92 (0·77, 1·09) | 0·84 (0·71, 1·00) | 0·92 (0·79, 1·07) |
| Rate per person year | 0·15 | 0·14 | 0·13 |  | p-value | 0·327 | 0·050 | 0·267 |
|  |  |  |  |  |  |  |  |  |
| *Birth before the 18th birthday* |  |  |  |  |  |  |  |  |
| n/N (%) | 203/959 (21 %) | 368/1931 (19 %) | 355/1846 (19 %) |  | HR (95% CI) | 0·87 (0·71, 1·07) | 0·84 (0·68, 1·03) | 0·96 (0·80, 1·16) |
| Rate per person year | 0·10 | 0·09 | 0·09 |  | p-value | 0·198 | 0·091 | 0·679 |

*Those who were pregnant at the time of randomisation and gave birth more than three months after randomisation, were assumed to have an induction time of nine months after the previous pregnancy ended

**Table S12a: Sensitivity analyses of the effects of the intervention packages on composite birth and marriage outcomes**

|  | **Control** | **Economic** | **Combined** |  |  | **Economic vs control** | **Combined vs control** | **Combined vs economic** |
| --- | --- | --- | --- | --- | --- | --- | --- | --- |
| *Birth within eight months of the end of the intervention period (incl. data collected up to 52-57 months after recruitment)* |  |  |  |  |  |  |  |  |
| n/N (%) | 286/989 (28·6%) | 524/1998 (26·2%) | 472/1902 (24·8%) |  | Age adjusted HR (95% CI) | 0·89 (0·75, 1·07) | 0·82 (0·69, 0·98) | 0·92 (0·79, 1·07) |
| Rate per person year | 0·11 | 0·097 | 0·091 |  | p-value | 0·209 | 0·029 | 0·271 |
|  |  |  |  |  |  |  |  |  |
| *Birth before the 18th birthday* |  |  |  |  |  |  |  |  |
| n/N (%) | 286/986 (29·0%) | 552/1976 (27·9%) | 532/1891 (28·1%) |  | HR (95% CI) | 0·92 (0·76, 1·11) | 0·88 (0·73, 1·08) | 0·96 (0·82, 1·14) |
| Rate per person year | 0·098 | 0·092 | 0·092 |  | p-value | 0·376 | 0·219 | 0·671 |

**Table S12b: Sensitivity analyses of the proportions who had given birth by the end of the trial with and without proxy information on births**

|  | **Control** | **Economic** | **Combined** |
| --- | --- | --- | --- |
| With proxy^#^ information on births |  |  |  |
| n/N (%) | 418/985 (42·2%) | 812/1990 (40·8%) | 755/1892 (39·9%) |
|  |  |  |  |
| Without proxy information on births |  |  |  |
| n/N (%) | 413/985 (41·9%) | 796/1989 (40·0%) | 747/1889 (39·5%) |

^#^ We received information from family members, teachers, friends, and neighbours indicating that 29 girls who were not reached in the final interview round had a child.

**Table S12c: Effects of the intervention packages on outcomes using inverse probability weighting to adjust for loss-to-follow-up**

|  | **Economic vs control** | **Combined vs control** | **Combined vs economic** |
| --- | --- | --- | --- |
| *Birth within eight months of the end of the intervention period (incl. data collected up to 52-57 months after recruitment)* |  |  |  |
| Age- adjusted HR (95% CI) | 0·88 (0·74, 1·05) | 0·81 (0·67, 0·97) | 0·90 (0·78, 1·05) |
| p-value | 0·151 | 0·020 | 0·184 |
|  |  |  |  |
| *Birth before the 18th birthday* |  |  |  |
| HR (95% CI) | 0·92 (0·76, 1·10) | 0·87 (0·72, 1·06) | 0·95 (0·81, 1·12) |
| p-value | 0·354 | 0·174 | 0·551 |
|  |  |  |  |
| *Sat for grade nine exams* |  |  |  |
| RR (95% CI) using log binomial regression^#^ | 1·15 (1·06, 1·24) | 1·23 (1·14, 1·33) | 1·08 (1·03, 1·14) |
| p-value | <0·0001 | <0·0001 | 0·002 |

^#^Log-binomial regression was used because the GEE model required constant weights within the clusters.

**Table S13: Effects of the intervention packages on the probability of sitting for grade nine exams according to exam registers from the DEBS offices**

|  | **Control** | **Economic** | **Combined** |  | **Economic vs control** | **Combined vs control** | **Combined vs economic** |
| --- | --- | --- | --- | --- | --- | --- | --- |
|  |  |  |  | RR (95% CI) | 1·22 (1·07, 1·39) | 1·29 (1·14, 1·47) | 1·06 (0·95, 1·19) |
| n/N (%) | 444/999 (44%) | 1114/2004 (56%) | 1157/1919 (60%) | p-value | 0·002 | <0·0001 | 0·311 |

**Table S14: Posthoc analysis of effects of the intervention packages on giving birth during and after the intervention period**

|  | **During intervention period** | | | | | | |  | **After intervention period** | | | | | | | |
| --- | --- | --- | --- | --- | --- | --- | --- | --- | --- | --- | --- | --- | --- | --- | --- | --- |
|  | **Control** | **Economic** | **Combined** |  | **Economic vs control** | **Combined vs control** | **Combined vs economic** |  |  | **Control** | **Economic** | **Combined** |  | **Economic vs control** | **Combined vs control** | **Combined vs economic** |
| *Birth* |  |  |  |  |  |  |  |  | |  |  |  |  |  |  |  |
| n/N (%) | 190/989 (19%) | 328/1998 (16%) | 283/1901 (15%) | Age-adj. HR (95% CI) | 0·85 (0·69, 1·04) | 0·75 (0·61, 0·92) | 0·88 (0·74, 1·05) |  | n/N (%) | 250/955 (26%) | 514/1960 (26%) | 496/1869 (27%) | Age-adj. HR (95% CI) | 1·03 (0·87, 1·22) | 1·04 (0·89, 1·21) | 1·01 (0·88, 1·16) |
| Rate per person year | 0·091 | 0·077 | 0·069 | p-value | 0·110 | 0·006 | 0·163 |  | Rate per person year | 0·17 | 0·18 | 0·18 | p-value | 0·757 | 0·642 | 0·887 |
|  |  |  |  |  |  |  |  |  | |  |  |  |  |  |  |  |
| *Birth before the 18th birthday* |  |  |  |  |  |  |  |  | |  |  |  |  |  |  |  |
| n/N (%) | 159/996 (16%) | 275/1981 (14%) | 247/1908 (13%) | HR (95% CI) | 0·85 (0·68, 1·06) | 0·75 (0·60, 0·94) | 0·89 (0·73, 1·08) |  | n/N (%) | 130/810 (16%) | 287/1662 (17%) | 297/1607 (18%) | HR (95% CI) | 1·04 (0·82, 1·30) | 1·09 (0·87, 1·37) | 1·05 (0·86, 1·29) |
| Rate per person year | 0·077 | 0·066 | 0·061 | p-value | 0·156 | 0·013 | 0·228 |  | Rate per person year | 0·13 | 0·14 | 0·15 | p-value | 0·761 | 0·440 | 0·606 |

**Table S15: Age-specific effects of the intervention packages on norms, sitting for grade nine exams and birth during the intervention period at randomisation**

|  | Age at randomisation |  | Control | Economic | Combined |  |  | **Economic vs control** | **Combined vs control** | **Combined vs economic** |
| --- | --- | --- | --- | --- | --- | --- | --- | --- | --- | --- |
| Birth during intervention period | 10-12 years | n/N (%) | 7/160 (4·4%) | 16/351 (4·6%) | 12/337 (3·6%) |  | HR (95% CI) | 1·03 (0·44, 2·40) | 0·73 (0·28, 1·94) | 0·71 (0·31, 1·64) |
|  | 13-15 years | n/N (%) | 132/708 (18·6%) | 241/1429 (16·9%) | 211/1353 (15·6%) |  | HR (95% CI) | 0·92 (0·72, 1·16) | 0·84 (0·65, 1·08) | 0·91 (0·75, 1·11) |
|  | 16-17 years | n/N (%) | 50/118 (42·4%) | 63/196 (32·1%) | 58/200 (29·0%) |  | HR (95% CI) | 0·64 (0·47, 0·89) | 0·58 (0·42, 0·80) | 0·90 (0·64, 1·28) |
|  | 18+ | n/N (%) | 1/3 (33·3%) | 8/22 (36·4%) | 2/11 (18·2%) |  | HR (95% CI) | - | - | - |
|  |  |  |  |  |  |  |  |  |  |  |
| Birth (whole follow-up period) | 10-12 years | n/N (%) | 16/160 (10·0%) | 28/351 (8·0%) | 31/337 (9·2%) |  | HR (95% CI) | 0·75 (0·43, 1·33) | 0·84 (0·48, 1·45) | 1·11 (0·71, 1·75) |
|  | 13-15 years | n/N (%) | 204/708 (28·8%) | 388/1429 (27·2%) | 352/1353 (26·0%) |  | HR (95% CI) | 0·95 (0·77, 1·17) | 0·90 (0·73, 1·11) | 0·95 (0·80, 1·12) |
|  | 16-17 years | n/N (%) | 62/118 (52·5%) | 92/196 (46·9%) | 79/200 (39·5%) |  | HR (95% CI) | 0·77 (0·58, 1·03) | 0·64 (0·48, 0·87) | 0·83 (0·63, 1·10) |
|  | 18+ | n/N (%) | 1/3 (33·3%) | 12/22 (54·6%) | 4/11 (36·4%) |  | HR (95% CI) | - | - | - |
|  |  |  |  |  |  |  |  |  |  |  |
| Sat for grade nine exams | 10-12 years | n/N (%) | 137/154 (88·9%) | 319/344 (92·7%) | 316/335 (94·3%) |  | RR (95% CI) | 1·05 (0·97, 1·13) | 1·07 (0·99, 1·15) | 1·02 (0·98, 1·07) |
|  | 13-15 years | n/N (%) | 444/682 (65·1%) | 1067/1398 (76·3%) | 1089/1329 (81·9%) |  | RR (95% CI) | 1·17 (1·06, 1·29) | 1·26 (1·15, 1·39) | 1·08 (1·02, 1·14) |
|  | 16-17 years | n/N (%) | 36/112 (32·1%) | 85/190 (44·7%) | 101/188 (53·7%) |  | RR (95% CI) | 1·38 (0·97, 1·95) | 1·65 (1·17, 2·32) | 1·19 (0·96, 1·48) |
|  | 18+ | n/N (%) | 0/3 (0%) | 10/21 (47·6%) | 7/10 (70%) |  | RR (95% CI) | - | - | 1·56 (0·71, 3·40) |
|  |  |  |  |  |  |  |  |  |  |  |
| Perceived community norms to accept early pregnancy outside marriage | 10-12 years | n/N (%) | 44/140 (31·4%) | 106/328 (32·3%) | 96/321 (29·9%) |  | RR (95% CI) | 1·00 (0·77, 1·29) | 1·06 (0·80, 1·40) | 1·06 (0·79, 1·43) |
|  | 13-15 years | n/N (%) | 253/611 (41·4%) | 463/1279 (36·2%) | 429/1236 (34·7%) |  | RR (95% CI) | 0·86 (0·73, 1·00) | 0·86 (0·73, 1·02) | 1·01 (0·85, 1·19) |
|  | 16-17 years | n/N (%) | 52/98 (53·1%) | 81/170 (47·7%) | 67/170 (39·4%) |  | RR (95% CI) | 0·86 (0·67, 1·11) | 0·75 (0·58, 0·96) | 0·87 (0·68, 1·11) |
|  | 18+ | n/N (%) | 2/3 (66·7%) | 6/18 (33·3%) | 3/10 (30%) |  | RR (95% CI) | 0·21 (0·04, 1·14) | 0·07 (0·01, 0·31) | 0·31 (0·12, 0·79) |
|  |  |  |  |  |  |  |  |  |  |  |
| Perceived community norms to support girls completing grade nine | 10-12 years | n/N (%) | 122/136 (89·7%) | 282/310 (91·0%) | 268/289 (92·7%) |  | RR (95% CI) | 1·03 (0·97, 1·10) | 1·04 (0·98, 1·11) | 1·01 (0·95, 1·08) |
|  | 13-15 years | n/N (%) | 450/546 (82·4%) | 1025/1165 (88·0%) | 982/1112 (88·3%) |  | RR (95% CI) | 1·07 (1·02, 1·13) | 1·08 (1·02, 1·14) | 1·01 (0·97, 1·05) |
|  | 16-17 years | n/N (%) | 71/92 (77·2%) | 119/145 (82·1%) | 128/148 (86·5%) |  | RR (95% CI) | 1·03 (0·92, 1·16) | 1·08 (0·96, 1·21) | 1·04 (0·95, 1·15) |
|  | 18+ | n/N (%) | 2/3 (66·7%) | 15/17 (88·2%) | 9/10 (90·0%) |  | RR (95% CI) | 2·13 (0·62, 7·26) | 1·93 (0·54, 6·86) | 0·91 (0·68, 1·22) |

**Table S16a: Association between reporting sexual activity previous 4 weeks and being in school in the fifth follow-up**

|  |  | **Control (n=855)** | **Economic (n=1801)** | **Combined (n=1741)** |
| --- | --- | --- | --- | --- |
| *Sexually active (fifth follow-up round)* |  |  |  |  |
| n/N (%) | Out of school | 86/230 (37·4%) | 129/264 (48·9%) | 89/207 (43·0%) |
|  | In school | 31/ 625 (5·0%) | 46/1537 (3·0%) | 57/1534 (3·7%) |
|  |  |  |  |  |
| RR (95% CI) | Out of school | Ref. |  |  |
|  | In school | 0·14 (0·10, 0·20) | 0·06 (0·05, 0·09) | 0·08 (0·06, 0·12) |

**Table S16b: Effects of the intervention packages on reporting being in school in the various follow-up rounds**

| **Follow-up round** | **Control** | **Economic** | **Combined** |  |  | **Economic vs control** | **Combined vs control** | **Combined vs economic** |
| --- | --- | --- | --- | --- | --- | --- | --- | --- |
| *Second follow-up round* |  |  |  |  |  |  |  |  |
| n/N (%) | 789/926 (85·2%) | 1780/1901 (93·6%) | 1750/1853 (94·4%) |  | RR (95% CI) | 1·10 (1·05, 1·14) | 1·11 (1·07, 1·16) | 1·02 (0·99, 1·04) |
|  |  |  |  |  | p-value | <0·0001 | <0·0001 | 0·171 |
| *Third follow-up round* |  |  |  |  |  |  |  |  |
| n/N (%) | 753/913 (82·5%) | 1704/1863 (91·5%) | 1696/1830 (92·7%) |  | RR (95% CI) | 1·11 (1·06, 1·16) | 1·13 (1·08, 1·18) | 1·02 (0·99, 1·04) |
|  |  |  |  |  | p-value | <0·0001 | <0·0001 | 0·180 |
| *Fourth follow-up round* |  |  |  |  |  |  |  |  |
| n/N (%) | 640/832 (76·9%) | 1594/1794 (88·8%) | 1578/1717 (91·9%) |  | RR (95% CI) | 1·15 (1·09, 1·22) | 1·21 (1·15, 1·28) | 1·05 (1·02, 1·08) |
|  |  |  |  |  | p-value | <0·0001 | <0·0001 | 0·002 |
| *Fifth follow-up round* |  |  |  |  |  |  |  |  |
| n/N (%) | 625/855 (73·1%) | 1537/1801 (85·3%) | 1534/1741 (88·1%) |  | RR (95% CI) | 1·17 (1·11, 1·24) | 1·21 (1·14, 1·28) | 1·04 (1·00, 1·07) |
|  |  |  |  |  | p-value | <0·0001 | <0·0001 | 0·057 |
| *Sixth follow-up round* |  |  |  |  |  |  |  |  |
| n/N (%) | 373/903 (41·3%) | 901/1870 (48·2%) | 850/1796 (47·3%) |  | RR (95% CI) | 1·19 (1·03, 1·36) | 1·17 (1·02, 1·35) | 0·99 (0·88, 1·10) |
|  |  |  |  |  | p-value | 0·015 | 0·027 | 0·831 |
| *Seventh follow-up round* |  |  |  |  |  |  |  |  |
| n/N (%) | 388/902 (43·0%) | 942/1865 (50·5%) | 911/1781 (51·2%) |  | RR (95% CI) | 1·17 (1·04, 1·33) | 1·20 (1·05, 1·36) | 1·02 (0·92, 1·13) |
|  |  |  |  |  | p-value | 0·011 | 0·005 | 0·717 |
| *Eighth follow-up round* |  |  |  |  |  |  |  |  |
| n/N (%) | 353/897 (39·4%) | 850/1855 (45·8%) | 844/1754 (48·1%) |  | RR (95% CI) | 1·15 (1·00, 1·32) | 1·20 (1·04, 1·39) | 1·05 (0·93, 1·18) |
|  |  |  |  |  | p-value | 0·052 | 0·010 | 0·448 |
| *Ninth follow-up round* |  |  |  |  |  |  |  |  |
| n/N (%) | 352/936 (37·6%) | 854/1927 (44·3%) | 882/1828 (48·2%) |  | RR (95% CI) | 1·17 (1·00, 1·35) | 1·27 (1·09, 1·47) | 1·09 (0·96, 1·23) |
|  |  |  |  |  | p-value | 0·046 | 0·002 | 0·190 |
| *Educational attainment in last interview conducted* |  |  |  |  |  |  |  |  |
| Mean (95% CI) | 8·92 (8·78, 9·06) | 9·35 (9·23, 9·46) | 9·35 (9·25, 9·46) |  |  |  |  |  |

**Figure S3: Percentage in school in each arm in follow-up rounds two to nine**

**Table S17: Self-reported total exam score in best six subjects from grade nine exams**

|  | **Control** | **Economic** | **Combined** |  |  | **Economic vs control** | **Combined vs control** | **Combined vs economic** |
| --- | --- | --- | --- | --- | --- | --- | --- | --- |
| Reported a total grade nine exam score n/N (%) | 420/999 (42%) | 1021/2004 (51%) | 1009/1919 (53%) |  |  |  |  |  |
| Mean (SD) score among those who reported a total exam score | 268·1 (5·5) | 286·0 (3·3) | 278·1 (3·4) |  | RD (95% CI) | 18·8 (2·6, 35·0) | 14·1 (-2·3, 30·6) | -4·7 (-17·4, 8·1) |
| Mean (SD) score (all participants) | 112·7 (4·8) | 145·7 (3·6) | 146·2 (3·6) |  | RD (95% CI) | 32·9 (14·9, 51·0) | 37·4 (18·4, 56·3) | 4·4 (-14·2, 23·1) |

**Table S18: Effects of the intervention packages on reported ability to express own opinions and make own decisions**

|  | **Control** | **Economic** | **Combined** |  |  | **Economic vs control** | **Combined vs control** | **Combined vs economic** |
| --- | --- | --- | --- | --- | --- | --- | --- | --- |
| Ever talked about romantic relationships or sexual issues with guardians (fourth follow-up; missing 688) |  |  |  |  |  |  |  |  |
| n/N (%) | 273/809 (33·8%) | 629/1759 (35·8%) | 619/1666 (37·2%) |  | RR (95% CI) | 1·09 (0·95, 1·26) | 1·15 (1·00, 1·32) | 1·05 (0·94, 1·18) |
|  |  |  |  |  | p-value | 0·215 | 0·042 | 0·375 |
|  |  |  |  |  |  |  |  |  |
| Married or has a boyfriend (final follow-up; missing 231) |  |  |  |  |  |  |  |  |
| n/N (%) | 531/936 (56·7%) | 1049/1927 (54·4%) | 951/1828 (52·0%) |  | RR (95% CI) | 0·98 (0·87, 1·01) | 0·91 (0·81, 1·02) | 0·93 (0·84, 1·02) |
|  |  |  |  |  | p-value | 0·735 | 0·101 | 0·127 |
| *Among those married or with boyfriend:* |  |  |  |  |  |  |  |  |
| Often or sometimes expresses own opinion when disagrees with husband (final follow-up; missing 0) |  |  |  |  |  |  |  |  |
| n/N (%) | 423/531 (79·7%) | 887/1049 (84·6%) | 816/951 (85·8%) |  | RR (95% CI) | 1·06 (1·01, 1·11) | 1·06 (1·01, 1·11) | 1·00 (0·96, 1·05) |
|  |  |  |  |  | p-value | 0·028 | 0·030 | 0·945 |
| Can initiate conversations about using contraception and condoms with husband/boyfriend (final follow-up; missing 16) |  |  |  |  |  |  |  |  |
| n/N (%) | 349/527 (66·2%) | 721/1043 (69·1%) | 701/945 (74·2%) |  | RR (95% CI) | 1·02 (0·92, 1·14) | 1·09 (0·99, 1·21) | 1·07 (0·99, 1·15) |
|  |  |  |  |  | p-value | 0·671 | 0·082 | 0·094 |

**Table S19: Pregnancy, marital status and timing of marriage and conception**

|  |  | **Control** | **Economic** | **Combined** | **Total** |
| --- | --- | --- | --- | --- | --- |
| *Pregnancy reported during follow-up period* |  |  |  |  |  |
| Not reported pregnancy | n/N (%) | 520/989 (52·6%) | 1113/1998 (55·7%) | 1070/1901 (56·3%) | 2703/4888 (55·3%) |
| Reported a pregnancy | n/N (%) | 469/989 (47·4%) | 880/1998 (44·0%) | 826/1901 (43·5%) | 2175/4888 (44·5%) |
| Missing information on pregnancy | n/N (%) | 0/989 | 5/1998 (0·3%) | 5/1901 (0·3%) | 10/4888 (0·2%) |
|  |  |  |  |  |  |
| *Time of conception in relation to the timing of marriage among those who reported a pregnancy* |  |  |  |  |  |
| Married before conception | n/N (%) | 74/469 (15·8%) | 129/880 (14·7%) | 90/826 (10·9%) | 293/2175 (13·5%) |
| Conception before marriage | n/N (%) | 394/469 (84·0%) | 749/880 (85·1%) | 734/826 (88·9%) | 1877/2175 (86·3%) |
| Timing of pregnancy not reported | n/N (%) | 1/469 (0·2%) | 2/880 (0·2%) | 2/826 (0·2%) | 5/2175 (0·2%) |
|  |  |  |  |  |  |
| *Ever reported being married by final follow-up* |  |  |  |  |  |
| Not married | n/N (%) | 749/999 (75·0%) | 1560/2004 (77·8%) | 1511/1919 (78·7%) | 3820/4922 (77·6%) |
| Married | n/N (%) | 250/999 (25·0%) | 444/2004 (22·2%) | 408/1919 (21·3%) | 1102/4922 (22·4%) |
|  |  |  |  |  |  |
| *Timing of marriage in relation to the time of conception among those who were married* |  |  |  |  |  |
| Reported not being pregnant | n/N (%) | 18/250 (7·2%) | 35/444 (7·9%) | 44/408 (10·8%) | 97/1102 (8·8%) |
| Married before conception | n/N (%) | 74/250 (29·6%) | 129/444 (29·1%) | 90/408 (22·1%) | 293/1102 (26·6%) |
| Married within nine months of conceiving | n/N (%) | 66/250 (26·4%) | 93/444 (20·9%) | 111/408 (27·2%) | 270/1102 (24·5%) |
| Married more than nine months after conception | n/N (%) | 92/250 (36·8%) | 185/444 (41·7%) | 161/408 (39·5%) | 438/1102 (39·7%) |
| Missing information on pregnancy or timing of pregnancy | n/N (%) | 0/250 (0·0%) | 2/444 (0·5%) | 2/408 (0·5%) | 4/1102 (0·4%) |

## Harms

Over the duration of the trial there were no deaths or hospitalisations that could be attributed to the intervention packages. The only unintended consequences we are aware of were that participants from some schools reported gossip and jealousy in the community arising because only some girls and no boys were benefitting from the support provided.

## References

1. Feyissa GT, Tolu LB, Soboka M, Ezeh A. Effectiveness of interventions to reduce child marriage and teen pregnancy in sub-Saharan Africa: A systematic review of quantitative evidence. *Front reprod health* 2023; **5**.

2. Desrosiers A, Betancourt T, Kergoat Y, Servilli C, Say L, Kobeissi L. A systematic review of sexual and reproductive health interventions for young people in humanitarian and lower-and-middle-income country settings. *BMC Public Health* 2020; **20**(1).

3. Peterson AJ, Donze M, Allen E, Bonell C. Effects of Interventions Addressing School Environments or Educational Assets on Adolescent Sexual Health: Systematic Review and Meta-analysis. *Perspect Sex Reprod Health* 2019; **51**(2): 91-107.

4. Oringanje C, Meremikwu MM, Eko H, Esu E, Meremikwu A, Ehiri JE. Interventions for preventing unintended pregnancies among adolescents. *Cochrane Database Syst Rev* 2016; **2**: CD005215.

5. Hindin MJ, Kalamar AM, Thompson TA, Upadhyay UD. Interventions to Prevent Unintended and Repeat Pregnancy Among Young People in Low- and Middle-Income Countries: A Systematic Review of the Published and Gray Literature. *J Adolesc Health* 2016; **59**(3): S8-S15.

6. Mason‐Jones AJ, Sinclair D, Mathews C, Kagee A, Hillman A, Lombard C. School‐based interventions for preventing HIV, sexually transmitted infections, and pregnancy in adolescents. *Cochrane Database Syst Rev* 2016; (11).

7. Lopez LM, Bernholc A, Chen M, Tolley EE. School-based interventions for improving contraceptive use in adolescents. *The Cochrane database of systematic reviews* 2016; (6): CD012249.

8. Estrada F, Atienzo EE, Cruz-Jimenez L, Campero L. A Rapid Review of Interventions to Prevent First Pregnancy among Adolescents and Its Applicability to Latin America. *J Pediatr Adolesc Gynecol* 2021; **34**(4): 491-503.

9. Ivanova O, Rai M, Michielsen K, Dias S. How Sexuality Education Programs Have Been Evaluated in Low-and Lower-Middle-Income Countries? A Systematic Review. *Int J Environ Res Public Health* 2020; **17**(21).

10. Shangase N, Kharsany ABM, Ntombela NP, Pettifor A, McKinnon LR. A Systematic Review of Randomized Controlled Trials of School Based Interventions on Sexual Risk Behaviors and Sexually Transmitted Infections Among Young Adolescents in Sub-Saharan Africa. *AIDS Behav* 2021; **25**(11): 3669-86.

11. Austrian K, Soler-Hampejsek E, Behrman JR, et al. The impact of the Adolescent Girls Empowerment Program (AGEP) on short and long term social, economic, education and fertility outcomes: a cluster randomized controlled trial in Zambia. *BMC Public Health* 2020; **20**(1): 349.

12. Austrian K, Soler-Hampejsek E, Kangwana B, et al. Impacts of Multisectoral Cash Plus Programs on Marriage and Fertility After 4 Years in Pastoralist Kenya: A Randomized Trial. *J Adolesc Health* 2022; **70**(6): 885-94.

13. Baird S, McIntosh C, Özler B. Cash or condition? Evidence from a cash transfer experiment. *Q J Econ* 2011; **126**(4): 1709-53.

14. Baird S, McIntosh C, Özler B. When the money runs out: Do cash transfers have sustained effects on human capital accumulation? *J Dev Econ* 2019; **140**: 169-85.

15. Bandiera O, Buehren N, Burgess R, et al. Women's Empowerment in Action: Evidence from a Randomized Control Trial in Africa. *Am Econ J Appl Econ* 2020; **12**(1): 210-59.

16. Berge LIO, Bjorvatn K, Makene F, Helgesson Sekei L, Somville V, Tungodden B. On the Doorstep of Adulthood: Empowering Economic and Fertility Choices of Young Women. Discussion paper: NHH, 2022. <https://hdl.handle.net/11250/3028686>

17. Buchmann N, Field E, Glennerster R, Nazneen S, Pimkina S, Sen I. Power vs money: Alternative approaches to reducing child marriage in Bangladesh, a randomized control trial, 2017.

18. Buehren N, Goldstein MP, Gulesci S, Sulaiman M, Yam V. Evaluation of an Adolescent Development Program for Girls in Tanzania. *World Bank Policy Research Working Paper* 2017; (7961).

19. Burke HM, Chen M, Murray K, et al. The effects of the integration of an economic strengthening and HIV prevention education programme on the prevalence of sexually transmitted infections and savings behaviours among adolescents: a full-factorial randomised controlled trial in South Africa. *BMJ global health* 2020; **5**(4): e002029.

20. Cabezon C, Vigil P, Rojas I, et al. Adolescent pregnancy prevention: An abstinence-centered randomized controlled intervention in a Chilean public high school. *J Adolesc Health* 2005; **36**(1): 64-9.

21. Cho H, Mbai I, Luseno WK, Hobbs M, Halpern C, Hallfors DD. School Support as Structural HIV Prevention for Adolescent Orphans in Western Kenya. *J Adolesc Health* 2018; **62**(1): 44-51.

22. Cowan FM, Pascoe SJS, Langhaug LF, et al. The Regai Dzive Shiri Project: a cluster randomised controlled trial to determine the effectiveness of a multi-component community-based HIV prevention intervention for rural youth in Zimbabwe--study design and baseline results. *Trop Med Int Health* 2008; **13**(10): 1235-44.

23. Cowan FM, Pascoe SJS, Langhaug LF, et al. The Regai Dzive Shiri project: results of a randomized trial of an HIV prevention intervention for youth. *AIDS* 2010; **24**(16): 2541-52.

24. Dake F, Natali L, Angeles G, et al. Cash Transfers, Early Marriage, and Fertility in Malawi and Zambia. *Stud Fam Plann* 2018; **49**(4): 295-317.

25. Duflo E, Dupas P, Kremer M. Education, HIV, and Early Fertility: Experimental Evidence from Kenya. *Am Econ Rev* 2015; **105**(9): 2757-97.

26. Dunbar MS, Kang Dufour M-S, Lambdin B, Mudekunye-Mahaka I, Nhamo D, Padian NS. The SHAZ! project: results from a pilot randomized trial of a structural intervention to prevent HIV among adolescent women in Zimbabwe. *PLoS One* 2014; **9**(11): e113621.

27. Dupas P, Duflo E, Kremer M. Estimating the impact and cost-effectiveness of expanding access to secondary education in Ghana. New Delhi: 3ie, 2016.

28. Duflo E, Dupas P, Kremer M. The Impact of Free Secondary Education: Experimental Evidence from Ghana. . Working Paper: Series NBoERWP: Research NBoE, 2021. <https://www.nber.org/papers/w28937>

29. Hallfors DD, Cho H, Rusakaniko S, et al. The Impact of School Subsidies on HIV-Related Outcomes Among Adolescent Female Orphans. *J Adolesc Health* 2015; **56**(1): 79-84.

30. Hallfors D, Cho H, Rusakaniko S, Iritani B, Mapfumo J, Halpern C. Supporting adolescent orphan girls to stay in school as HIV risk prevention: evidence from a randomized controlled trial in Zimbabwe. *Am J Public Health* 2011; **101**(6): 1082-8.

31. Handa S, Peterman A, Huang C, Halpern C, Pettifor A, Thirumurthy H. Impact of the Kenya Cash Transfer for Orphans and Vulnerable Children on early pregnancy and marriage of adolescent girls. *Soc Sci Med* 2015; **141**: 36-45.

32. Jewkes R, Nduna M, Levin J, et al. Impact of stepping stones on incidence of HIV and HSV-2 and sexual behaviour in rural South Africa: cluster randomised controlled trial. *BMJ* 2008; **337**: a506.

33. Kangwana B, Austrian K, Soler-Hampejsek E, et al. Impacts of multisectoral cash plus programs after four years in an urban informal settlement: Adolescent Girls Initiative-Kenya (AGI-K) randomized trial. *PLoS One* 2022; **17**(2): e0262858.

34. Mbizvo MT, Kasonda K, Muntalima N-C, et al. Comprehensive sexuality education linked to sexual and reproductive health services reduces early and unintended pregnancies among in-school adolescent girls in Zambia. *BMC Public Health* 2023; **23**(1): 348.

35. Özler B, Hallman K, Guimond M-F, Kelvin EA, Rogers M, Karnley E. Girl Empower – A gender transformative mentoring and cash transfer intervention to promote adolescent wellbeing: Impact findings from a cluster-randomized controlled trial in Liberia. *SSM - Population Health* 2020; **10**: 100527.

36. Pettifor A, MacPhail C, Hughes JP, et al. The effect of a conditional cash transfer on HIV incidence in young women in rural South Africa (HPTN 068): a phase 3, randomised controlled trial. *The Lancet Global health* 2016; **4**(12): e978-e88.

37. Pike C, Coakley C, Ahmed N, et al. Goals for girls: a cluster-randomized trial to investigate a school-based sexual health programme amongst female learners in South Africa. *Health Educ Res* 2023; **38**(5): 375-91.

38. Ross DA, Changalucha J, Obasi AI, et al. Biological and behavioural impact of an adolescent sexual health intervention in Tanzania: a community-randomized trial. *AIDS* 2007; **21**(14): 1943-55.

39. Doyle AM, Ross Da Fau - Maganja K, Maganja K Fau - Baisley K, et al. Long-term biological and behavioural impact of an adolescent sexual health intervention in Tanzania: follow-up survey of the community-based MEMA kwa Vijana Trial. *PLoS Med* 2010; **7**(6): e1000287.

40. Taylor M, Jinabhai C, Dlamini S, Sathiparsad R, Eggers MS, De Vries H. Effects of a teenage pregnancy prevention program in KwaZulu-Natal, South Africa. *Health Care Women Int* 2014; **35**(7-9): 845-58.

41. Waidler J, Gilbert U, Mulokozi A, Palermo T. A "Plus" Model for Safe Transitions to Adulthood: Impacts of an Integrated Intervention Layered onto A National Social Protection Program on Sexual Behavior and Health Seeking among Tanzania's Youth. *Stud Fam Plann* 2022; **53**(2): 233-58.

42. Zulaika G, Kwaro D, Nyothach E, et al. Menstrual cups and cash transfer to reduce sexual and reproductive harm and school dropout in adolescent schoolgirls: study protocol of a cluster-randomised controlled trial in western Kenya. *BMC Public Health* 2019; **19**(1): 1317.

43. Zulaika G, Nyothach E, van Eijk AM, et al. Menstrual cups and cash transfer to reduce sexual and reproductive harm and school dropout in adolescent schoolgirls in western Kenya: a cluster randomised controlled trial. *EClinicalMedicine* 2023; **65**: 102261.

44. Sandøy IF, Mudenda M, Zulu J, et al. Effectiveness of a girls’ empowerment programme on early childbearing, marriage and school dropout among adolescent girls in rural Zambia: study protocol for a cluster randomized trial. *Trials* 2016; **17**(1): 588.

45. Blystad A, Moland KM, Munsaka E, Sandøy I, Zulu J. Vanilla bisquits and lobola bridewealth: parallel discourses on early pregnancy and schooling in rural Zambia. *BMC Public Health* 2020; **20**(1): 1485.

46. Zulu JM, Sandøy IF, Moland KM, Musonda P, Munsaka E, Blystad A. The challenge of community engagement and informed consent in rural Zambia: an example from a pilot study. *BMC Med Ethics* 2019; **20**(1): 45.

47. Handa S, Natali L, Seidenfeld D, Tembo G. The impact of Zambia’s unconditional child grant on schooling and work: results from a large-scale social experiment. *J Dev Eff* 2016; **8**(3): 346-67.

48. Kenya Adolescent Reproductive Health Program, Program for Appropriate Technology in Health (PATH), Population Council. Tuko Pamoja. Adolescent Reproductive Health and Life Skills Curriculum. Nairobi, Kenya, 2006.
