# Supplementary material for: Effectiveness of economic support, comprehensive sexuality education and community dialogue on early childbearing and sitting for grade nine exams among adolescent girls in rural Zambia: a cluster randomised trial
Source: eClinicalMedicine. 2024 Nov 15;78:102934. doi: 10.1016/j.eclinm.2024.102934 (PMC11609475; doi:10.1016/j.eclinm.2024.102934)
Supplement: Trial protocol [file mmc3.docx]

**Cluster randomized trial on the effectiveness of a girls’ empowerment programme on early childbearing, marriage and school dropout among adolescent girls in rural Zambia**

Short name: Research Initiative to Support the Empowerment of Girls (RISE)

Contents

[Protocol summary 3](#_Toc437605811)

[Trial registration: 5](#_Toc437605812)

[Protocol version 5](#_Toc437605813)

[Funding: 5](#_Toc437605814)

[Roles and responsibilities: 5](#_Toc437605815)

[1. INTRODUCTION 6](#_Toc437605816)

[Background 6](#_Toc437605817)

[Rationale 11](#_Toc437605818)

[Trial objectives 11](#_Toc437605819)

[*Primary objectives:* 11](#_Toc437605820)

[*Secondary objectives:* 11](#_Toc437605821)

[Hypotheses 12](#_Toc437605822)

[Primary 12](#_Toc437605823)

[Secondary 12](#_Toc437605824)

[2. TRIAL METHODOLOGY 12](#_Toc437605825)

[Trial design 12](#_Toc437605826)

[Study setting 12](#_Toc437605827)

[Randomization units 12](#_Toc437605828)

[Participant population 12](#_Toc437605829)

[Eligibility criteria 12](#_Toc437605830)

[Participant retention 13](#_Toc437605831)

[Community sensitisation 13](#_Toc437605832)

[Interventions 14](#_Toc437605833)

[*‘Economic support’ arm* 14](#_Toc437605834)

[*‘Combined intervention’ arm* 15](#_Toc437605835)

[Outcomes 16](#_Toc437605836)

[*Primary outcomes:* 16](#_Toc437605837)

[*Secondary outcomes:* 16](#_Toc437605838)

[Participant timeline 18](#_Toc437605839)

[Sample size 19](#_Toc437605840)

[Recruitment 22](#_Toc437605841)

[Randomization and blinding/masking 22](#_Toc437605842)

[Data collection methods 23](#_Toc437605843)

[Data management and quality assurance 24](#_Toc437605844)

[Statistical methods 25](#_Toc437605845)

[Cost-Benefit Analysis (CBA) 26](#_Toc437605846)

[Qualitative Data Analysis 27](#_Toc437605847)

[Data monitoring 27](#_Toc437605848)

[Harms 28](#_Toc437605849)

[Benefits 28](#_Toc437605850)

[Auditing 28](#_Toc437605851)

[3. ETHICS AND DISSEMINATION 29](#_Toc437605852)

[Research ethics approval 29](#_Toc437605853)

[Protocol amendments 29](#_Toc437605854)

[Consent or assent 29](#_Toc437605855)

[Confidentiality 30](#_Toc437605856)

[Declaration of interests 30](#_Toc437605857)

[Access to data 30](#_Toc437605858)

[Dissemination plan 30](#_Toc437605859)

[4. RESEARCH TRAINING OPPORTUNITIES OFFERED BY THE STUDY 30](#_Toc437605860)

[References 31](#_Toc437605861)

[Appendix 1 – Project timelines 34](#_Toc437605862)

[Appendix 2 – SRH Curriculum of youth club 36](#_Toc437605863)

# Protocol summary

**Background**

Adolescent pregnancies carry risks to the young mothers and the baby. Keeping girls in school can potentially protect girls from getting pregnant. In Zambia, 35% of young rural girls have given birth by the age of 18 years, and the pregnancy rates are particularly high among girls who are out-of-school. Approximately 50% of girls never enroll in secondary school. A number of studies have found that economic support to girls and/or their families can increase school enrolment and attendance, and three trials have found effects on postponement of childbearing and marriage. Other studies indicate that widespread myths and negative social norms are barriers to adolescent girls using modern contraceptives, thus contributing to high rates of early pregnancy. However, there is little robust research from Africa on how sexual and reproductive health programmes can be delivered in a way that actually affects early marriage and pregnancy rates.

**Purpose**

To measure (1) the effect on marriage and pregnancy rates of providing a unique package of economic support to girls and their families that targets both the poverty dimension and the school drop-out dimension of adolescent pregnancy and marriage in a rural Zambian context, and (2) whether economic support combined with a community approach targeting beliefs related to education and sexual and reproductive health (SRH), can have an even stronger impact on the same outcomes.

**Design**

Cluster randomized controlled trial with three arms with clusters being basic schools with surrounding communities.

**Study population**

The participant population will be girls enrolled in grade 7 in January in 2016 in rural schools in twelve study districts: Monze, Mazabuka, Chikankata, Kapiri Mposhi, Kabwe, Chibombo, Chisamba, Mkushi, Luano, Pemba, Kalomo and Choma.

**Study size**

Approximately 4900 girls and 157 clusters, that is 1000 girls and 31 clusters in the control arm and 1950 girls and 63 clusters in each of the intervention arms. The rationale for having different numbers of clusters in the three arms is that we expect larger differences between each of the intervention arms and the control arm than between the two intervention arms themselves.

**Intervention**

One intervention arm will be offered economic support in the form of monthly unconditional cash transfers to the participating girl and her parents and payment of school fees in 2017 and 2018 if the girl attends junior secondary school. The second intervention arm will be offered the same economic support combined with a community component comprising community meetings about the value of education for adolescent girls and the risks related to early childbearing, and a youth club for girls and boys (both in- and out-of-school). The aims of the community component are to change social norms and normative, behavioural and control beliefs relating to education, SRH, early pregnancy and marriage for adolescent girls.

**Duration and Follow-up**

The duration of the trial, from recruitment to the last follow-up survey will be 4 years and 10 months. A baseline survey will be conducted just after recruitment. Afterwards there will be short face-to-face or phone-based follow-up contacts with all the participants every six months.

**Primary objectives**

1. To measure the effectiveness and cost-benefit of providing economic support to girls and their families on adolescent childbearing and grade 9 completion in a Zambian context
2. To measure whether combining economic support to girls and their families with a community intervention targeting social norms and normative, behavioural and control beliefs regarding education and sexual and reproductive health, is more effective and cost-beneficial in reducing adolescent childbearing and increasing grade 9 completion than economic support alone.

**Primary outcomes**

- Incidence of births within 8 months of the end of the intervention
- Incidence of births before girls’ 18^th^ birthday;
- Proportion of girls who sit for grade 9 exam

Trial registration: <http://www.clinicaltrials.gov>

Protocol version: 14.11.2019

Funding: The Research Council of Norway will fund the trial through its Centres of Excellence scheme to the Centre for Intervention Science in Maternal and Child Health (CISMAC; project number 223269) and through the GLOBVAC programme. The University of Bergen (UiB) and the University of Zambia (UNZA) will fund the positions of the involved researchers from their institution, and the University of Bergen will fund fellowships for one PhD candidate and 2.5 postdoctoral fellows who will be involved in the project. Statens Lånekasse will fund one additional PhD candidate (Quota scholarship).

Roles and responsibilities:

*Trial sponsors:* Centre for Intervention Science in Maternal and Child Health (CISMAC) and the University of Zambia

*Project management team:*

Principal investigator: Ingvild Fossgard Sandøy, Centre for International Health (CIH)/Department of Global Public Health and Primary Care (IGS), University of Bergen (UiB)

Co-principal investigator: Patrick Musonda, School of Medicine, Department of Public Health, University of Zambia (UNZA)

Other team members:

Joseph Zulu, School of Medicine, Department of Public Health, UNZA

Ecloss Munsaka, School of Education, Department of Educational Psychology, UNZA

Mpundu Makasa, School of Medicine, Department of Public Health, UNZA

Mweetwa Mudenda, School of Medicine, Department of Public Health, UNZA

Choolwe Jacobs, School of Medicine, Department of Public Health, UNZA

Linda Kampata, School of Medicine, Department of Public Health, UNZA

Charles Michelo, School of Medicine, Department of Public Health, UNZA

Astrid Blystad, CIH/IGS, UiB

Karen Marie Moland, CIH, UiB

Knut Fylkesnes, CIH, UiB

Richard Banda, Central Statistical Office, Zambia, and CIH, UiB

Ottar Mæstad, Chr. Michelsen’s Institute (CMI), Norway

Bertil Tungodden, CMI and Norwegian School of Economics (NHH)

Amani Mori, CIH/IGS, UiB

Andrea Melberg, CIH/IGS, UiB.

# 1. INTRODUCTION

## Background

Adolescent pregnancies carry substantial risks to young mothers and their babies. A considerable proportion of pregnancies among adolescent girls are unplanned, and such pregnancies have a higher risk of termination by unsafe abortion. Complications of childbearing and abortion are together estimated to be the most common causes of death in girls aged 15-19 in low and middle income countries (LMICs) [1]. The risk of premature birth and a having low birth weight baby is also higher for adolescent girls [2, 3], with consequent higher morbidity and mortality risks for the child [4], and the risk of neonatal mortality is estimated to be >50% higher for babies born to mothers aged <20 years compared to mothers aged 20-29 [2, 5]. The younger the mother is, the higher is the risk of complications both for her and the child, and pregnancies below age 16 are of particular concern [3]. Nevertheless, approximately 7.3 million girls below age 18 give birth in low and middle income countries every year [6].

Early pregnancy is often closely interlinked with early marriage and school dropout, and poverty is an important factor contributing to all three. Numerous observational studies from low-income countries indicate that young women who quit school early are more likely to marry and become pregnant at an early age and engage in riskier sexual activities than those who stay in school longer and achieve a higher educational attainment [7-10]. Increased levels of schooling have also been found to be associated with better health of women themselves and their children [11]. In the last decades there has been a strong focus on increasing primary school enrolment in many countries, and these efforts have been quite successful, but there has been less focus on secondary education. Enrolment at secondary level is much lower than at primary level in most LMICs, particularly for girls, due to longer distances, higher economic barriers (e.g. higher fees), or due to girls becoming pregnant. Many girls quit school early or are never enrolled because their families are unable to pay school fees, buy uniforms and/or rely on their labour at home. Moreover, when family resources are constrained, there may be a preference to support boys’ education rather than girls’ [12]. Marrying off a girl may be regarded as a better way to secure her future than schooling, and the bride-price paid to the girl’s family may be an important source of income. Once a girl is married, she is expected to start childbearing. Where access to cash is limited, unmarried girls report the possibility of receiving gifts and cash as a motivation to get involved in sexual relationships with boys or men [13, 14]. Thus many unmarried adolescent girls engage in sexual relationships even in settings where it is not socially acceptable.

In preparation for this project, we did a systematic search^[[1]](#footnote-2)^ in 16 databases^[[2]](#footnote-3)^ for randomized controlled trials (RCTs) in LMICs that measured the effectiveness of various interventions on adolescent childbearing and marriage. The reference list of recent systematic reviews on unintended pregnancy [15], adolescent childbearing [16] and early marriage [17] were also examined to detect relevant original papers. The identified trials examined two main groups of interventions: economic support to reduce the cost of schooling [18-21], and sexual and reproductive health education [22-24]. Three trials examined the effects of some kind of economic support to girls to reduce the cost of schooling. In a trial in Kenya, girls who were provided with free school uniforms were less likely to drop out before completing primary school. Proxy-reports by classmates (which have a questionable validity), indicated reduced risks of early marriage and childbearing [21]. A cluster-randomized trial in Zimbabwe found that a programme targeted at orphan girls in grade 6 offering payment of school fees, free uniform and a school-based assistant who dealt with problems of absenteeism, led to an 80% reduction in school dropout, increased school attendance, and 60% reduction in marriage rates in the next 2 years [25]. A cluster-randomized trial in Malawi found that payment of school fees combined with small cash transfers to adolescent girls and their families resulted in lower prevalence of HIV and Herpes simplex virus type 2 (HSV-2) in the next 18 months among baseline school-girls [20]. The trial randomly allocated school-girls to one of three arms: a conditional cash transfer (CCT), an unconditional cash transfer (UCT) and a control arm, and a comparison of the three arms indicated that school retention and attendance, cognitive test scores and English reading comprehension were higher in the CCT than in the UCT arm. In the UCT arm, marriage and pregnancy rates were 48% and 34% lower, respectively, compared to the control arm in the next two years despite only a slight increase in school attendance. No significant change was seen in marriage and pregnancy rates among schoolgirls in the conditional (CCT) arm [19]. Thus, this trial indicates that unconditional economic support may have a greater impact on adolescent childbearing and marriage than conditional support. The timing of support also seems to be important, and our hypothesis is that payment of school fees, which reduces the opportunity cost of school, may be decisive at stages when children approach transition points in the education system, e.g. between primary and secondary school, when costs would otherwise increase. In the present project we plan to examine the effects of an economic package that combines UCT and payment of school fees because this approach might combine the beneficial effects of both the unconditional cash transfer on marriage and pregnancy rates and the conditional cash transfer on school attendance.

The systematic literature review identified two randomized trials that studied the impact of sexual and reproductive health (SRH) education programmes on early childbearing and marriage in LMICs. An RCT in Kenya found that pupils exposed to a curriculum informing them of the higher risk of HIV infection associated with having older sexual partners, had 28% lower pregnancy rates 12 months later compared to the control arm [23], whereas a comparison of control and intervention schools where teachers were trained in the national HIV/AIDS curriculum promoting sexual abstinence until marrriage, indicated no difference in the risk of adolescent childbearing after 2,3, 5 and 7 years [23, 26, 27]. The only difference was that those who became pregnant in the latter intervention arm, were more likely to be married [27, 28]. The latter findings are in line with the conclusions of a systematic review of randomized studies assessing abstinence-only programmes in the US: there were no effects on self-reported sexual behaviour or STI diagnosis, and no protective effects on pregnancies [29]. A cluster randomized trial in Tanzania examined the effects of an intervention with the following four components: (1) a teacher-led, peer-assisted, SRH school programme in grades 5–7, which included information on condom use (approx. twelve 40-minute school sessions per year); (2) training of health workers in youth-friendly health services, followed by supervision; (3) community-based condom promotion and distribution by and for adolescents; and (4) one week of community-wide mobilization in each community and annual youth health weeks. An evaluation three years after the start of the intervention found effects on SRH knowledge and self-reported condom use among the youths, but no impact on the incidence of HIV, HSV-2 or pregnancy despite high coverage and high quality of the implementation [24]. A survey conducted among young people in the intervention and control communities more than 8 years after the start of the programme did not find any differences in the biological outcomes either. The authors mention that qualitative process evaluation during the first years of the intervention indicated that adolescents found it difficult to use the knowledge and skills acquired through the programme because of attitudes and practices in the wider community. The authors suggest that this may imply that SRH education may not be effective on its own, but may need to be combined with efforts to “address broader sexual norms and expectations” and future aspirations [22, 30, 31]. The community component in our project draws on these findings.

#### The Zambian context

In Zambia, 35% of young rural girls have given birth by the age of 18, and the median age at marriage for girls in rural Zambia was 17.8 years in 2007 [7]. According to the 2010 Zambia Census of Population and Housing (ZCPH) the pregnancy-related mortality ratio among girls aged 15-19 years is 80% higher than among those aged 20-24 [32]. The adolescent childbearing rates are much higher in rural than urban areas, and higher among girls who are out-of-school compared to those who are still attending (36% vs. 5% at age 17 in rural areas) (unpublished data from 2010 ZCPH). Unmarried adolescent girls who become pregnant are often forced to marry the father of the baby to avoid shame or economic insecurity. Adolescent pregnancy within marriage is viewed as highly desirable compared to out of wedlock pregnancies [13].

#### Preparatory work

**Figure 1: Project implementation**

The project is part of the portfolio of the Centre for Intervention Science in Maternal and Child Health (CISMAC), a Centre for Excellence (CoE) funded by the Research Council of Norway (RCN) and the UiB. CISMAC’s vision is to conduct excellent trials in LMICs to generate evidence on how to effectively and equitably deliver promising health interventions in a way that can be scaled up and made relevant in other contexts, and thus to inform national and global policies in adolescent, maternal and child health.

The project consists of several stages (Figure 1) and is guided by the PRECEDE-PROCEED model (Greene and Kreuter 2005, cited in [33]) which is a tool for planning and evaluating health behaviour change programmes using both qualitative and quantitative methods. It utilises an ecological approach, focusing not only on the individual and his/her cognitions, knowledge, attitudes, skills and behaviour, but also on how these are located within a socio-cultural and economic environment. PRECEDE, which refers to the planning stage, includes 1) social assessment, 2) epidemiological assessment, 3) behavioural/environmental assessment, 4) educational/ecological assessment (which includes identification of predisposing, reinforcing and enabling factors), and 5) administrative/policy analysis. PROCEED involves implementation, process evaluation and end-point evaluation. The PRECEDE-PROCEED model does not dictate the use of theory, but the planning model is often combined with the use of behaviour change theories, e.g. the Theory of Planned Behaviour, as these theories can be used to identify important predisposing, reinforcing and enabling factors [33].

We have conducted two phases of formative research following the PRECEDE part of the model. Process evaluation was used in the pilot study and both process and end point evaluation will be utilized in the trial. The first phase of the formative research explored factors and processes that contribute to early childbearing and marriage and school dropout in the cultural and socioeconomic contexts of Monze, Mazabuka and Chikankata districts in Zambia. In-depth interviews (IDIs) and focus group discussions (FGDs) were conducted with girls, parents, teachers, health workers and community leaders in December 2014 and January 2015. The analysis of the qualitative interviews followed a classical approach employing Malterud’s ‘Systematic text condensation’ [34]. The social assessment revealed that the main explanations provided for school drop-out, early pregnancy and early marriage were overlapping and interlinked. Poverty was brought up as a reason for all three; many families cannot afford to buy uniforms or pay school fees at secondary level (primary school in Zambia is free, but secondary is not); parents may put pressure on their daughters to get sexual partners or to become pregnant to obtain the bride price and economic support for their daughters; and girls mentioned a desire for gifts/money as an important reason for having sex with a boyfriend. Pregnancy was described as leading to school drop-out and marriage, although some girls were reported to reenter school after having given birth. Several informants mentioned that many parents did not encourage their daughters to continue with school because they did not see the benefit of it, and instead encouraged them to get married. Girls being regarded as ready for marriage after menarche and school-dropout, and substantial social barriers to seeking contraceptives, were reported as other factors leading to girls getting pregnant. The latter is in line with reports from many Zambian districts indicating that youth friendly health services often exist only on paper, and that access to effective contraception for young girls may be limited because of the distance to health clinics, and because health care providers may express reluctance and moralistic dismay when young people seek contraceptives [13].

The formative research also detected some common behavioral beliefs which put girls at risk of getting pregnant: many of the girls believed that there was minimal risk of getting pregnant if they had sex a few days after the end of the menstrual period (i.e. they failed to take into account that the length of the “safe period” depends on the length of the menstrual cycle), and there was a common misconception that young girls could end up as infertile if they used hormonal contraception before their first pregnancy. The SRH curriculum in Zambian schools is rather comprehensive on paper, but the interviews with teachers and pupils indicates that in practice teachers tend to focus on sexual abstinence as the way to avoid getting pregnant or infected with a sexually transmitted infection (STI), including HIV. Usually teachers will deliver the content in an instructional manner and pupils frequently do not dare to ask questions. Hence sexually active young people commonly depend on advice from their peers on how to protect themselves, and misconceptions about modern contraceptives can prevail.

As part of the epidemiological assessment we analysed data from the 2010 ZCPH on childbearing among adolescent girls and found that 11% of girls in Monze, Mazabuka and Chikankata had given birth at age 16 and 38% at age 18. To examine trends in early childbearing since 2010 we visited 6 health facilities in Monze, Mazabuka and Chikankata and extracted age data from ANC registers, and this showed that the proportions of pregnant women attending ANC who were below 16 and 18 years were stable during the period 2010-2014.

Since early marriage and pregnancy in Zambia have multiple causes, our behavioural and environmental assessment implies that it is likely that a programme that targets several contributing factors will have a greater effect than a single intervention. Furthermore, as long as approximately 50% of girls never enroll in secondary school and the number of places available at secondary level is restricted [35], any realistic trajectory of future school enrolment implies that many girls will remain out of school in the foreseeable future, and efforts to prevent early marriage and childbearing therefore also need to reach out-of-school girls. Informed by our literature review, repeated discussions (before the formative research) with national and key stakeholders such as the Ministry of Education (MoE), the Ministry of Community Development and Maternal and Child Health (MoCDMCH), Forum for African Women Educationalists in Zambia (FAWEZA), Campaign for Female Education (Camfed) and UNFPA, concrete suggestions from local stakeholders during the formative research, and the Theory of planned behaviour^[[3]](#footnote-4)^, we developed an intervention package that targets what we have identified as the main causes of early pregnancy: (1) An economic component targeting the poverty dimension and the school drop-out dimension, with the aim to increase school attendance and secondary school enrolment, and to reduce parental pressure for early marriage, and reduce girls’ dependence on having a boyfriend to meet her desire for possessions or cash; and (2) A community component that targets social norms, normative, behavioural and control beliefs regarding education, SRH, contraceptive use and marriage among adolescent girls. The latter component aims to address the lack of robust research from LMICs on sexual and reproductive health programmes that target both youth in- and out-of-school and the wider community.

A policy assessment indicates that both components have potential for scale-up and are in line with political priorities in Zambia. A welfare program in the form of cash transfers is a particularly relevant approach to reduce the poverty dimension in Zambia. Three kinds of unconditional social cash transfer models have been implemented in a number of districts in recent years (Old Age Based Pension Scheme, the Inclusive Model for the 10% most incapacitated households, and the Child Grant for families with a child under 5 years) [37]. It is also noteworthy that the government has announced that it aims to make secondary education free in order to remove economic barriers to education (However, the actual implementation of this promise in the next decade will depend on the relative priority the government will give to secondary education compared to other areas that have also been promised increased investments.) Payment of school fees and a cash transfer that can cover uniforms and school materials are therefore also in line with planned policies, which is an important aspect of sustainability considerations. It is also worth mentioning that a robust evaluation of these interventions is in line with the WHO’s recommendations for research on programmes to prevent early childbearing and marriage: The effects of school retention, economic incentives, interventions to empower girls, and interventions to change community norms on age at first childbearing and age at marriage are mentioned as promising approaches that should be further examined [5].

In the second phase of formative research in March 2015 we explored the acceptability of the developed intervention package. Many of the informants believed that cash transfers could make a difference in terms of reducing the temptation among girls to get into early relationships with men and in terms of reducing the cost of schooling. Some informants found the amounts we proposed (ZMW 30) to be too limited, whereas others believed these sums could potentially make a difference (not the least the girls themselves). Many respondents stressed that it would be important to have close monitoring to ensure that the funds reach the right recipients. The community component with SRH education as part of youth club meetings and community meetings to discuss the situation of adolescent girls was strongly endorsed by all the informants.

After the completion of both phases of the formative research we had follow-up meetings with the MoE, MoCDMCH, FAWEZA, Camfed and other stakeholders to present our findings and discuss the intervention components. All the stakeholders supported that the planned interventions were relevant and feasible for the government to implement later on if the trial finds that they are effective.

We conducted a small pilot study in Chibombo district in September 2015. This gave us an opportunity to test the feasibility of some of the trial components. The pilot showed that it is important to engage all levels of the formal and informal leadership structures in the communities before starting recruitment efforts in order to avoid rumours about the purpose of the project. It also became clear that it is essential to explain thoroughly to parents and girls that they are not making any commitments by signing the consent and assent forms, and that there will be no bad consequences to them if the girl drops out of school, gets married or pregnant.

## Rationale

This will be the first cluster RCT to measure the effect of a package combining economic support and a community component to prevent adolescent childbearing in a LMIC. A number of studies have found that economic support to girls and/or their families can increase school enrolment and attendance [38-41], and a couple of trials have found effects on postponement of childbearing and marriage [18, 19, 25]. Increased schooling among adolescent girls is likely to empower them economically [42] and cognitively, and to enable them to better protect the health of their children [11] and themselves. A meta-analysis has found that an increase of 1 year in the education of women is associated with 6.5-9.9% lower under five mortality in LMICs [43]. Other studies indicate that widespread myths and negative social norms are barriers to adolescent girls using modern contraceptives, thus contributing to high rates of early pregnancy. However, there is little robust research from Africa on how sexual and reproductive health programmes can be delivered in a way that actually affects early marriage and pregnancy rates. Delaying first marriage is likely to contribute to delayed age at first pregnancy, and a lower adolescent pregnancy rate is likely to reduce morbidity and mortality among women [1] and children [2, 5]. The need to find ways to prevent early marriage and pregnancy are high on the political agenda in Zambia.

## Trial objectives

### *Primary objectives:*

1. To measure the effectiveness and cost-benefit of providing economic support to girls and their families on adolescent childbearing and grade 9 completion in a Zambian context
2. To measure whether combining economic support to girls and their families with a community intervention targeting social norms and normative, behavioural and control beliefs regarding education and sexual and reproductive health, is more effective and cost-beneficial in reducing adolescent childbearing and increasing grade 9 completion than economic support alone.

### *Secondary objectives:*

1. To measure the effectiveness of economic support alone and a combined intervention on early marriage among adolescent girls
2. To measure the effectiveness of economic support alone and a combined intervention on secondary school enrolment, school attendance, dropout, exam scores and employment among adolescent girls
3. To measure the impact of economic support alone and a combined intervention on the socioeconomic distribution of adolescent childbearing and marriage
4. To measure the effectiveness of economic support alone and a combined intervention on changing normative beliefs related to girls’ education, early marriage, modern contraceptive use, and adolescent pregnancy.
5. To measure the effectiveness of economic support alone and a combined intervention on knowledge about sexual and reproductive health, use of modern contraceptives and behavioural, normative and control beliefs related to modern contraceptives, and self-reported sexual risk behaviour among adolescent girls

## Hypotheses

### Primary

1. The economic support will reduce the incidence of births before girls’ 18th birthday by at least 25% and increase the proportion who completes grade 9 by 15%;
2. The combined intervention (with economic support and the community component) will reduce the incidence of births within 8 months of the end of the intervention period and the incidence of births before girls’ 18th birthday by at least 40%, and increase the proportion who completes grade 9 by 26.5%.

### Secondary

1. Both interventions will significantly reduce school dropout, and increase secondary school enrolment, school attendance, exam scores and employment among adolescent girls.
2. Both interventions will significantly reduce early marriage, sexual risk behaviour and increase modern contraceptive use since the economic component will increase girls’ motivation to avoid pregnancy, and the community component will change behavioural and normative beliefs concerning contraceptive use.

# 2. TRIAL METHODOLOGY

## Trial design

The intervention packages will be examined in a cluster randomized controlled trial with two intervention arms and one control arm.

## Study setting

The trial will be conducted in twelve districts in Zambia: Monze, Mazabuka, Chikankata, Kapiri Mposhi, Kabwe, Chisamba, Chibombo, Mkushi, Luano, Pemba, Kalomo and Choma. These districts have been selected as they have medium school drop-out rates, and adolescent marriage and childbearing is common (unpublished data from 2010 ZCPH).

## Randomization units

#### A randomization unit or cluster will be a school offering grades 1-9. All the schools should be accessible from the District Educational Board Secretary’s (DEBS) office all year round and should have reasonably good mobile network coverage. Clusters will be purposively selected to ensure sufficient corridors (i.e. areas that are not included) between them in order to minimize the risk of contamination.

## Participant population

#### The participant population will be girls enrolled in grade 7 (average age approx. 14 years) in 2016 in rural basic schools in the twelve study districts.

## Eligibility criteria

All girls enrolled in grade 7 in the included schools will be eligible to participate, including those who are already married or have children. Girls without a parent or legal guardian living in the household will also be eligible to participate if the headmaster of their school or the headman of the village gives consent. There will be no age restriction.

Girls who drop out of school after they have been recruited will continue to be part of the trial.

## Participant retention

We will invest substantial resources to minimize bias and potential confounding by endeavouring to ensure more than 90% follow-up of participants. We will also invest resources to ensure that the follow-up percentage does not differ more than 5% between the three trial arms. The participants will receive a letter with the telephone numbers to the study’s main office and local trial supervisor so that they can inform project staff about any changes in their place of residence or primary contact telephone number. In addition, contact details will be updated by phone twice per year. As a token of appreciation of their time, the participants will be given a small compensation (of approx. 3-4 USD) when they respond to a few questions about current residence, school enrolment, marital status and childbirths, and when they participate in baseline and the final-up surveys. (Girls in the intervention arms will not receive compensation for answering questions during the follow-up contacts at the same time as they are receiving economic support, only after this support has come to an end. If they do not respond to questions during the intervention period, the economic support will be stopped.) If participants cannot be interviewed face-to-face, attempts to interview them by phone will be made. In case their phone number changes, relatives or neighbours we have recorded the contact details of will be asked to provide current contact details for girls. Girls who consent to participate in the trial but refuse to receive the intervention, will still be followed-up.

If we experience substantial post-randomization drop out, we will introduce additional incentives to keep participants in the trial.

## Community sensitisation

An important key to the success of the recruitment process and the intervention packages is the support from traditional leaders, other community leaders and the wider community. To achieve community acceptance chiefs, headmen, religious and informal leaders, headmasters and parent-teacher association members will be oriented and asked to support the trial activities before the recruitment is initiated. Local radio will also be used for community sensitization. If overall >15% of the families/girls in consenting schools decline participation, measures will be made to reduce refusals to participate to below 10%. Any individual school in which >20**%** of the girls do not consent to participate will not be included in the study.

After randomization, further consultative meetings will be held in all the trial arms for the same key groups, and the headmen and headmasters in the intervention areas will be engaged in the promotion of both intervention packages and in the implementation of the community component.

## Interventions

### The interventions will be launched in September 2016 and last for 28 months, until November 2018 (end of the academic year when the girls who attend school are expected to complete grade 9). In the control arm, girls will be offered some writing materials (exercise books, pencils and pens) as an incentive to participate. This is not likely to be sufficient to have any effect on the primary or secondary outcomes. Apart from this, only standard school and health services will be offered. The interventions will be delivered through personnel in the school and the health system to facilitate potential scale-up in Zambia and similar contexts.

### *‘Economic support’ arm*

In this intervention arm girls and their parents/guardians will be offered economic support, which will consist of UCTs for the girl (ZMW 30/month) and her parents/guardians (ZMW 350/year) and payment of school fees for girls who enrol in grade 8 and 9. The UCT amount corresponds to the approximately K70 provided monthly (in 2014) in the government’s Child Grant scheme for families with children under 5 [44], and it is only slightly higher than the 8 USD provided per month to poor households through the Inclusive Model [37]. Thus this amount is likely to be within the range that the government would consider if it were to implement a cash transfer programme for families with adolescent girls. This is estimated to be sufficient to pay for a school uniform and shoes, a school bag, books and writing materials, and the combination of the UCT and payment of school fees would thus make schooling free of cost for the family of an adolescent girl.

The money for the girls and the guardians will be disbursed by a cash transfer committee consisting of a teacher and two parents from the Parent-Teacher Association (PTA) committee. At least two of the cash transfer committee members will be present during disbursement to witness that the right persons receive the cash. If the girl moves, the money will be transferred via a mobile bulk transfer system. This implies that the recipient of the cash transfer must be a person above age 16 with a registered sim card. For girls who are younger than 16 years, one of the parents/guardians will be selected as the recipient of the parent/guardian cash transfer. Every month this parent/guardian will receive a text message with a unique reference number from the mobile transfer operator and this code can be used to obtain cash at the nearest agent. The parents/guardians will be informed that they should be accompanied by the girl when collecting the cash. Girls who are aged 16 or older and who have a registered sim card can receive the monthly cash transfer themselves. To ensure that all the recipients receive what they will be entitled to, questions will be asked in every follow-up contact about how much they have received, and all participants will be encouraged to contact the study team if they do not receive the amount they believe they are entitled to. The economic support will be stopped for girls who do not respond to questions in the follow-up contacts.

The payment of school fees will be made directly to the school account for girls who get a place in grade 8 and 9. There will be no age limit for receiving the economic support for those girls who are in school, but the economic support will stop after the 18^th^ birthday for girls who drop out of school.

The support package targets the key actors in the decisions leading to early pregnancy and marriage. UCTs target the poverty dimension, by making it less urgent for the parents/guardians that the girl gets married and less urgent for the girl to receive gifts from a boy-friend. The payment of school fees (combined with the UCT) targets the school drop-out dimension, with the combination removing economic barriers to the girl continuing in school. Staying in school in turn reduces the likelihood that she becomes married or pregnant. In sum, both these effects may reduce early pregnancies.

### *‘Combined intervention’ arm*

The second intervention arm will combine the economic support component with a community-oriented strategy, aiming to reduce early marriage and childbearing through (1) community and parent meetings promoting supportive social norms around education for girls and postponement of early marriage and early childbearing; and 2) establishment or strengthening of existing youth clubs in order to increase knowledge of sexual and reproductive health (SRH), including modern contraceptives, and change behavioural and control beliefs relating to contraceptive use among in- and out-of school adolescent girls and boys. We expect that the combination of these strategies will reduce early sexual activity and increase use of modern contraceptives and thus reduce pregnancy rates among adolescent girls, as well as further reducing school-dropout and delaying age of marriage compared to providing economic support alone, thus affecting childbearing rates also indirectly.

The SRH education will be delivered as part of a youth club that will have meetings twice per month in the school or community. Girls participating in the trial and boys who attend the same grades as these girls will be invited to participate in a youth club, and they will all be allowed to continue in the youth club even if they drop out/quit school. The meetings will include interactive discussions on education and employment opportunities, early marriage, the risks of early pregnancy, misconceptions around modern contraceptives, and skills training, e.g. negotiation of modern contraceptives use with a sexual partner. We will base the content of the meetings on the “Tuko Pamoja. Adolescent Reproductive Health and Life Skills Curriculum” developed by the Kenya Adolescent Reproductive Health Program, Program for Appropriate Technology in Health (PATH), and the Population Council [45], with some adaptations to fit the purpose of this trial. (The curriculum will be called “Tuli Antoomwe” in Tonga and “Tuli Pamo” in Bemba.) This interactive curriculum gives detailed descriptions of the content and activities of each meeting. We will add some sessions (e.g. on the value of education, initiation rites, early marriage, school re-entry and SRH rights). The sessions we have prepared was reviewed by experts from the Ministry of Health in Zambia to ensure that they were based on sound pedagogical principles and that the content is in line with the Zambian framework for Comprehensive SRH education. Appendix 2 provides a list of all the sessions that are planned. Some sessions may be repeated or extended over several meetings. Girls and boys will be split in separate groups when sensitive issues are discussed. In some meetings there will also be time for games and sports. Refreshments will be provided in the meetings to attract girls and boys to turn up.

To run the youth clubs, we will test a model where a community health assistant (CHAs)^[[4]](#footnote-5)^ or community workers (CHW) is linked with a teacher from the school in the cluster to form a community team. This team will collaborate in providing comprehensive SRH education to girls and boys both in- and out-of school, and the CHA/CHW and the teacher will take the lead role for some sessions each. The trial supervisor will supervise the youth club activities. A meeting will be held once a year to inform parents about the content of the future youth club sessions.

For each combined intervention cluster, two young (<20 years) unmarried women from the local community will be selected as youth peer educators. These youth peer educators should be girls who are well liked and respected among the girls in grade 7. The role of these peer educators will be to mobilize girls and boys to come to youth club meetings.

Meetings with the community at large will be organized twice per term by the trial supervisor in collaboration with headmen and the teacher and CHA/CHW in the community team. These meetings and the parent meetings will be conducted using a dialogue approach [49]. The discussion in such meetings should be free and democratic, although the persons leading the meetings may gently guide the discussion in a certain direction. Group discussions may contribute to behaviour change because the conversation can change people’s understanding of what is acceptable or desirable (i.e. their normative beliefs) and thus the social norms in the community. The hope is that the community members will agree on some action points related to the topic discussed since people often feel committed by group decisions if they perceive that they arrived at the decision to change a behaviour voluntarily [50]. If group decisions are made, these will be discussed again in ensuing meetings.

In community- and youth club meetings, films or role plays presenting simple messages will be used to start discussions. Two films (of 10-15 minutes duration each) have been produced. The two films that have been produced focus on early marriage, early pregnancy and the value of education. The films are drama-based and convey stories of girls with readily recognizable lives (girls in and out of school, girls with early and later pregnancies, unmarried girls, girls within early marital unions etc). A key notion is that the films should not reveal strong negative sentiments or disgust for certain life trajectories, but present messages in a locally adapted, respectful and nuanced way that calls for reflection and opens up for discussion. The production of the films has been subcontracted to Kula Music Studio Ltd, a Zambia-based non-profit, non-religious organization, which has previously made films and documentaries on reproductive health topics, and also has expertise in scripting, filming, and editing.

Before the launch of the combined intervention, a comprehensive 5 day training of the selected teachers, CHAs/CHWs, and the trial supervisor will be conducted and will include the SRH curriculum, facilitation techniques and community mobilization. The youth peer educators will be given 3 days training in peer counselling and reproductive health. The trainings will be carried out by the research team with some support from subcontracted persons from NGOs carrying out similar programmes.

## Outcomes

### *Primary outcomes:*

- incidence of births within 8 months of the end of the intervention period;
- incidence of births before girls’ 18^th^ birthday;
- proportion of girls who sit for grade 9 exam.

### *Secondary outcomes:*

#### Pregnancy and childbearing

- proportion of girls who have ever given birth after 2 years of programme implementation;
- proportion of girls who have ever been pregnant after 2 years of programme implementation;
- proportion of girls who have ever been pregnant before their 16^th^ birthday;
- proportion of girls who have ever given birth before their 16^th^ birthday;
- proportion of girls who have ever been pregnant before their 18^th^ birthday;
- socioeconomic equity in proportion of girls who have ever given birth before 18^th^ birthday;

#### Marriage

- proportion of girls ever married/cohabiting before their 18^th^ birthday;
- socioeconomic equity in proportion of girls ever married/cohabiting before 18^th^ birthday;
- proportion of unmarried girls with boyfriend

#### School-related

- school attendance in grade 8 and grade 9;
- enrolment grade 8;
- school drop-out rate before grade 9 completed (will include both regular drop out as well as “push-out”, the latter referring to a child not able to obtain a place at the next level of education because of lack of places);
- examination scores grade 9;
- enrolment grade 10;
- socioeconomic equity in participation in grade 9 exam among girls,

#### Other reproductive health outcomes

- sexually active last 4 weeks
- current modern contraceptive use (including use of condom, intrauterine device and hormonal contraception) at 4 years follow-up;
- miscarriage and pregnancy termination;
- knowledge of modern contraceptives;

#### Attitudes and beliefs

- perceived community norms regarding education among girls
- perceived community norms regarding modern contraceptive use among unmarried adolescents,
- perceived community norms regarding early marriage,
- perceived community norms regarding adolescent pregnancy

#### Other outcomes

- employment 4.5 years after recruitment;

*Background information to be collected*: household wealth, number of siblings, household members, educational attainment of parents/guardians, age and sex of head of household, number of lifetime sexual partners,.

## Participant timeline

|  | | Time  point | 2016 | | | | | | | | | | | | 2017 | | | | | | | | | | | | | 2018 | | | | | | | | | | | | | 2019 | | | | | | | | | | | | | 2020 | | | | | | | | | | | |  |
| --- | --- | --- | --- | --- | --- | --- | --- | --- | --- | --- | --- | --- | --- | --- | --- | --- | --- | --- | --- | --- | --- | --- | --- | --- | --- | --- | --- | --- | --- | --- | --- | --- | --- | --- | --- | --- | --- | --- | --- | --- | --- | --- | --- | --- | --- | --- | --- | --- | --- | --- | --- | --- | --- | --- | --- | --- | --- | --- | --- | --- | --- | --- | --- | --- | --- | --- |
|  |  |  | | J | F | M | A | M | J | J | A | S | O | N | D | | J | F | M | A | M | J | J | A | S | O | N | D | | J | F | M | A | M | J | J | A | S | O | N | D | | J | F | M | A | M | J | J | A | S | O | N | D | | J | F | M | A | M | J | J | A | S | O | N |
| Enrolment | Recruitment | |  |  | X | X | X | X |  |  |  |  |  |  | |  |  |  |  |  |  |  |  |  |  |  |  | |  |  |  |  |  |  |  |  |  |  |  |  | |  |  |  |  |  |  |  |  |  |  |  |  | |  |  |  |  |  |  |  |  |  |  |  |  |
|  | Eligibility screening | |  |  | X | X | X | X |  |  |  |  |  |  | |  |  |  |  |  |  |  |  |  |  |  |  | |  |  |  |  |  |  |  |  |  |  |  |  | |  |  |  |  |  |  |  |  |  |  |  |  | |  |  |  |  |  |  |  |  |  |  |  |  |
|  | Informed consent/assent | |  |  | X | X | X | X |  |  |  |  |  |  | |  |  |  |  |  |  |  |  |  |  |  |  | |  |  |  |  |  |  |  |  |  |  |  |  | |  |  |  |  |  |  |  |  |  |  |  |  | |  |  |  |  |  |  |  |  |  |  |  |  |
|  | Parent meeting | |  |  | X | X | X | X |  |  |  |  |  |  | |  |  |  |  |  |  |  |  |  |  |  |  | |  |  |  |  |  |  |  |  |  |  |  |  | |  |  |  |  |  |  |  |  |  |  |  |  | |  |  |  |  |  |  |  |  |  |  |  |  |
|  | Parental consent | |  |  | X | X | X | X |  |  |  |  |  |  | |  |  |  |  |  |  |  |  |  |  |  |  | |  |  |  |  |  |  |  |  |  |  |  |  | |  |  |  |  |  |  |  |  |  |  |  |  | |  |  |  |  |  |  |  |  |  |  |  |  |
|  | Allocation | |  |  |  |  |  |  | X |  |  |  |  |  | |  |  |  |  |  |  |  |  |  |  |  |  | |  |  |  |  |  |  |  |  |  |  |  |  | |  |  |  |  |  |  |  |  |  |  |  |  | |  |  |  |  |  |  |  |  |  |  |  |  |
| Interventions | Information meeting for girls, parents and communities | |  |  |  |  |  |  |  | X | X | X |  |  | |  |  |  |  |  |  |  |  |  |  |  |  | |  |  |  |  |  |  |  |  |  |  |  |  | |  |  |  |  |  |  |  |  |  |  |  |  | |  |  |  |  |  |  |  |  |  |  |  |  |
|  | UCT | |  |  |  |  |  |  |  |  | X | X | X | X | | X | X | X | X | X | X | X | X | X | X | X | X | | X | X | X | X | X | X | X | X | X | X | X |  | |  |  |  |  |  |  |  |  |  |  |  |  | |  |  |  |  |  |  |  |  |  |  |  |  |
|  | Payment school fees | |  |  |  |  |  |  |  |  |  |  |  |  | | X |  |  |  |  |  |  |  |  |  |  |  | | X |  |  |  |  |  |  |  |  |  |  |  | |  |  |  |  |  |  |  |  |  |  |  |  | |  |  |  |  |  |  |  |  |  |  |  |  |
|  | Youth club meetings | |  |  |  |  |  |  |  |  | X | X | X |  | | X | X | X |  | X | X | X |  | X | X | X |  | | X | X | X |  | X | X | X |  |  |  |  |  | |  |  |  |  |  |  |  |  |  |  |  |  | |  |  |  |  |  |  |  |  |  |  |  |  |
|  | Community meetings | |  |  |  |  |  |  |  |  | X |  | X |  | | X |  | X |  | X |  | X |  | X |  | X |  | | X |  | X |  | X |  | X |  | X |  | X |  | |  |  |  |  |  |  |  |  |  |  |  |  | |  |  |  |  |  |  |  |  |  |  |  |  |
| Assessments | Baseline interview | |  |  | X | X | X | X |  |  |  |  |  |  | |  |  |  |  |  |  |  |  |  |  |  |  | |  |  |  |  |  |  |  |  |  |  |  |  | |  |  |  |  |  |  |  |  |  |  |  |  | |  |  |  |  |  |  |  |  |  |  |  |  |
|  | Follow-up contacts | |  |  |  |  |  |  |  |  |  | X | X | X | |  |  |  | X | X | X | X |  | X | X | X | X | |  |  | X | X | X | X |  |  | X | X | X | X | |  |  | X | X | X | X |  |  | X | X | X | X | |  | X | X | X | X |  |  |  |  |  |  |  |
|  | Final survey | |  |  |  |  |  |  |  |  |  |  |  |  | |  |  |  |  |  |  |  |  |  |  |  |  | |  |  |  |  |  |  |  |  |  |  |  |  | |  |  |  |  |  |  |  |  |  |  |  |  | |  |  |  |  |  |  | X | X | X | X | X |  |

## Sample size

The sample size is estimated based on the primary outcomes “incidence of births within 8 months of the end of the intervention period” (average age expected to be 17.5 years), “incidence of births before girls’ 18^th^ birthday” and “proportion of girls who sit for grade 9 exam”. We used the 2010 census age-specific estimates of the proportions of 17 and 18 year old girls who ever given birth in the study districts to estimate the proportions in the control arm. The census indicates that 22% of 17 year old and 35% of 18 year old girls in the study districts had ever given birth. Based on this we assume that 27% of girls in the control arm will have given birth before their 18^th^ birthday.

All estimates below have been increased with 10% to compensate for a possible loss to follow up. The sample-size for this cluster-randomized controlled trial was calculated using PASS 14 (NCSS Statistical Software, Utah, US).

*Assumptions for sample size required to measure the primary outcome “birth within 8 months of the end of the intervention period” (*when the average age will be 17.1 years)

| **Parameter** | **Assumed level** | **Comment** |
| --- | --- | --- |
| Incidence of births in control group | 0.06 | Eight months after the end of the intervention period the girls will be on average 17.1 years and we assume that 15% of them will have given birth. This corresponds to an average incidence rate of (15%-3%)/2= 6% over this 2 year period. |
| Effectiveness of combined intervention vs control | -40% | i.e. incidence in combined intervention arm assumed to be 0.036 |
| Cluster size | 28 | See assumptions for the outcome “incidence of births before girls’ 18th birthday” |
| Person years per cluster | 56 | If 28 participants are followed up for 2 years, there will be 56 person years |
| Coefficient of variation (k) | 0.2 | The ICC was 0.00737 for “ever pregnant” after cash transfer trial in Malawi. This corresponds to k=0.20 when the total proportion who have given birth by this time is 0.15 |
| Z_1_ (acceptable alpha error level) | 1.96 |  |
| Power for comparison of combined intervention vs control | 90% | We need 36 combined clusters vs 36 control clusters to have 90% power detect the assumed difference. The PASS function for proportions indicates that with 63 combined clusters, we will have >90% power with 30 control clusters. |
|  |  |  |

*Assumptions for sample size required to measure the primary outcome “birth before 18^th^ birthday”*

| **Parameter** | **Assumed level** | **Comment** |
| --- | --- | --- |
| Incidence of births before girls’ 18th birthday | 0.08 | We assume that 27% of girls in the control arm will have given birth before their 18th birthday. This corresponds to an average incidence rate of (27%-3%)=8% per year over the average 3 year period (from the time the average age is 15). |
| Effectiveness (i.e. (1-RR)×100 of economic intervention vs control | -25% | I.e. the incidence in economic intervention arm assumed to be 0.06 |
| Effectiveness of combined intervention vs control | -40% | i.e. the incidence in combined intervention arm assumed to be 0.048 |
| Effectiveness of combined intervention vs economic intervention | -20% | The combined intervention will offer [1-(0.0.048/0.06)], i.e. 20% more relative protection than the economic intervention alone. |
| Cluster size | 28 | Average number of girls in grade 7 in the selected schools is 31. If we assume that up to 10% may be lost to follow up by the time of measuring the outcome, the average cluster size will be 28 |
| Person years per cluster | 84 | If 28 participants are followed up for 3 years on average, person years per cluster are 84. |
| Coefficient of variation (k) | 0.15 | The ICC was 0.00737 for “ever pregnant” after intervention period in cash transfer trial in Malawi (estimate obtained from Sarah Baird). This corresponds to k=0.15 when the total proportion who have given birth by this time is 0.27 [51]. |
| Z_1_ (acceptable alpha error level) | 1.96 |  |
| Power for comparison of economic intervention vs combined intervention | 70% | We need 63 clusters in each of the intervention arms to have 70% power to detect the assumed difference |
| Power for comparison of economic intervention vs control | 80% | We need 39 clusters in each arm to have 80% power to detect the assumed difference. The PASS power calculator for incidence rates does not allow for unequal trial arms, but the PASS function for proportions indicates that 63 economic and 31 control will give slightly higher power^[[5]](#endnote-2)^ |
| Power for comparison of combined intervention vs control | >95% | We need 23 clusters in each arm to have 95% power to detect the assumed difference. |
|  |  |  |

*Assumptions for sample size required to measure the primary outcome ‘proportion of girls who sit for grade 9 exam’*

| **Parameter** | **Assumed level** | **Comment** |
| --- | --- | --- |
| Proportion of girls who sit for grade 9 exam in control arm | 0.70 |  |
| Effectiveness of combined intervention vs control | +26.5% | i.e. proportion completing in combined intervention arm assumed to be 0.886 |
| Effectiveness (i.e. (1-RR)×100 of economic intervention vs control | +15% | i.e. proportion completing in economic intervention arm assumed to be 0.805 |
| Effectiveness of combined intervention vs economic intervention | +10% | The combined intervention will offer a [1+(0.886/0.805)] , i.e. 10% relative increase compared to the economic intervention alone. |
| Cluster size | 28 | See assumptions for the outcome “incidence of births before girls’ 18th birthday” |
| ICC | 0.02 | We have no information on the ICC for this outcome but have assumed it to be higher than for pregnancy |
| Z_1_ | 1.96 |  |
| Power for comparison of economic intervention vs combined intervention | 95% | We need 29 clusters in each of the intervention arms to have 95% power to detect the assumed difference |
| Power for comparison of economic intervention vs control | 95% | We need 24 clusters in each of the arms to have 95% power to detect the assumed difference |
| Power for comparison of combined intervention vs control | >95% | We need 7 clusters in each arm to have 95% power to detect the assumed difference |

Taking the largest of these sample sizes, we need 63 clusters in each of the intervention arms. Since we expect larger differences between each of the intervention arms and the control arm than between the two intervention arms themselves, we can reduce the total number of clusters by allowing for a lower sample size in the control arm. Thus we will include at least 63 economic intervention clusters, 63 combined intervention clusters and 31 control clusters, i.e. 157 clusters with a total of 31 x 157=4867 or approximately 4900 girls..

**Figure 2: Flow chart of trial**


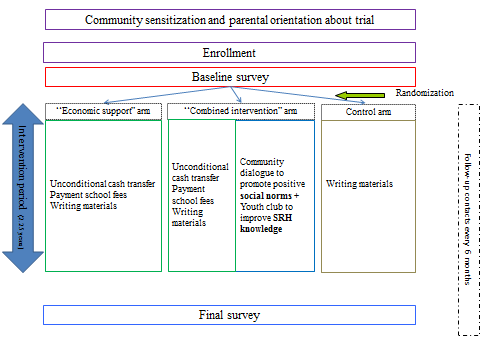


## Recruitment

Research assistants will between March and June 2016, i.e. in terms 1 and 2, visit selected schools (with grade 7) and inform girls who are enrolled in grade 7 about the study. The parents/guardians of girls in grade 7 will be invited to an information meeting. If their daughter is < 18 years, they will be asked to give consent to her participation in the trial. A few days later the research assistants will return to the school and ask girls to consent (if they are ≥18 years) or assent (if they are younger than 18 years and their parents or legal guardians have consented) to participation. Those who consent/assent and participate in the baseline survey will be enrolled.

Repeat visits will be made to the school to give girls who are absent on the day of the visit a chance to be enrolled.

Addresses and mobile phone numbers of the girl (if any), parents/guardians and close relatives or neighbours will be recorded when consent/assent is obtained. The girl will be asked to specify the preferred telephone number that can be used to make phone-based follow-up contacts with her.

## Randomization and blinding/masking

Randomization will take place in July 2016, after recruitment and baseline interviews. Randomization will be stratified by district. For each of 6 areas (each area corresponding to 2 districts) approximately 1000 allocations of the clusters will be computer-generated by an independent scientist tasked by CISMAC. Each allocation will be numbered. We will organize six randomization ceremonies where schools will be randomly allocated to one of the three trial arms. Officials from the study districts, head teachers and PTA chairpersons of the trial schools and chiefs will be invited to be present, and others, including community members, will be welcome to come too. Tickets will be drawn from a box with numbers corresponding to a specific allocation of clusters to the three arms.

There will be no blinding of participants, but the team doing the final survey will be independent from the intervention delivery and efforts will be put in place to keep them unaware of the intervention/control status of respondents. Biannual follow-up contacts with the participants and the final surveys will be conducted by an independent team of research assistants, most of them located in Lusaka.

## Data collection methods

A baseline interview will be conducted as part of the recruitment. As part of the trial there will be short phone-based or physical follow-up contacts with all the participants twice per year to update contact details and sociodemographic information. A final follow-up survey will also be conducted. Data will be captured electronically using tablets during face-to-face interviews and using tablets or computers during phone-based surveys and follow-up contacts.

Qualitative and quantitative process evaluation will take place throughout the intervention period in order to understand how the intervention works. Such monitoring activities will also allow identification of needs to make adjustments to the intervention delivery (the intervention will be adaptive [54]). In addition, incentivized lab experiments will be included.

We will collect information about the location and size of pre-randomization non-consenting schools, and information about age and village of girls who do not assent or whose guardians to do not consent, in order to assess the likely degree and direction of selection bias in the study

All the tools will be translated to Tonga, Nyanja, Lenje and Bemba, the dominating local languages in the study districts, and then back-translated to ensure that the content is maintained. Interviews will be conducted in English, Tonga, Nyanja, Lenje or Bemba depending on what the participant prefers.

##

**Table 1: Data collection elements**

| **Activity** | **Description** |
| --- | --- |
| **Baseline survey** | The baseline survey with girls enrolled in grade 7 will consist of interviewer-administered face-to-face interviews and will be carried out at school. We will employ and train a team of research assistants and a supervisor in each district to conduct this survey. The questionnaire is attached. |
| **Monitoring of school attendance** | Attendance reports will be collected at the end of the intervention period for all girls in the trial. |
| **Follow-up contacts** | All the girls in the intervention and control arms will be contacted every 6 months to update contact information and ask questions about school attendance, employment, marital status and childbearing, health care seeking, and whether they have received the cash transfers and/or participated in the youth club meetings. To monitor adverse events that can be attributed to the trial, girls will also be asked whether they have experienced problems due to their participation in the trial. In the fourth follow-up interview (taking place during the first two quarters of 2018) additional questions will be asked about communication about sexual and reproductive health issues.  In order to make it easier for the participants to respond to the more sensitive questions, Audio Computer Assisted Self-Interviewing (ACASI) will be used for questions 69-82. This will be done by giving the participants headsets so they can listen to the questions being read to them and they will be given privacy to fill in the correct responses on the tablets themselves. ACASI-technology has been shown to increase participants’ perceptions of privacy and anonymity and it appears to reduce social desirability bias [52, 53]. In addition, the item count technique [54, 55] will be used. This is an indirect questioning technique where the participants indicate how many of a set of statements are true for them, rather than whether each of the individual statements are true or false. If the girl cannot be found at school or at home at the time of the follow-up interview, call centre research assistants will attempt to reach her by phone.  In the third follow-up round we experienced challenges in several of the control schools with finding the participants because many of them were not in school any more. In order to motivate the participants to come to the school to be interviewed, we will increase the transport allowance provided for control participants from K20 to K50 per interview in 2018 and further to K75 in 2019 and K100 for the final interview in 2020. For the participants in the intervention arms, we do not think it is necessary to provide such an allowance during the period when they are receiving economic support, and thus a transport allowance of K75 / K100 will only be provided for interviews in 2019 and 2020. The participants will then have finished grade 9 and we expect a large proportion of them to move away from the study areas, either to go to grade 10 in a secondary school or to get married or work. This implies that they will either have to travel to come back for interviews and need to be compensated for the costs and time that this requires. And for those that move far away, they will have to keep their phone number active for us to be able to interview them over the phone and many of them will also have to call us to inform us when they will be available, and thus the compensation should be sufficient to cover the costs of this.  Around the time of the fourth follow-up interviews with the RISE participants in the first half of 2018, their parents/guardians will also be invited to come to parent meetings where they will be asked take part in an interview. Parents/guardians who do not come to the meeting will be sought at home. The questions in the parent interview pertain to expenses they have had in the previous 6-12 months related to food, education, health care and other expenditures, and about their attitudes to early marriage, early childbearing, contraceptive use and education. A travel allowance of K50 will be offered to the guardians who are interviewed.  The research assistants will be asked to take selfies of themselves with the participants to document that they have interviewed the correct participants. The trial participants in most cases live far away from each other and the research assistants who do the interviews travel alone (or with a driver) on a motorbike to find the participants. Thus, even if there is a supervisor who makes unannounced monitoring visits to observe the research assistants twice per month, most of the interviews are done without other project staff being able to observe them. We are worried that some of the research assistants may be tempted to manufacture some of the interviews, particularly when it is difficult to find participants because they have moved (which is the case for an increasing proportion of our participants). We expect that such pictures will make fabrication of interviews less likely. |
| **Qualitative process evaluation** | The trial supervisors at district level will be present at 1/6-1/3 of the youth club meetings to observe the quality of the delivery of the community component. They will record their observations and give feedback to the community teams. IDIs and FGDs will be conducted every year during the intervention period to explore experiences of purposively selected participants’ (from within the categories girls, boys, parents/guardians, other community members) with the various intervention components, reasons for non-participation in the intervention, and their perspectives on the accessibility of SRH services. Draft interview guides are attached. |
| **Quantitative process evaluation** | We will collect administrative data on school performance and school attendance of all study participants from school registers in the study districts. In addition to the information obtained about participation in the intervention components during followup contacts, we will also collect attendance list from the each youth club meeting, logs with counts of how many persons show up will also be saved for all parent – and community meetings. We will moreover monitor whether similar activities take place in the control clusters.  In addition, we will design incentivized experiments that will provide unique data on the causal effects of the interventions on the participants’ risk-, time-, and social preferences. These experiments will follow standard protocols in behavioral economics, see for example Almås, Cappelen, Salvanes, Sørensen, and Tungodden (2012) [55]. |
| **Final follow-up survey** | A phone-based final follow-up survey will be conducted in the last quarter of 2020. If the girl cannot be reached via phone, field-based research assistants will attempt to find her. At this point almost all the girls will be more than 18 years. The questionnaire will be developed later and will include measurements of pregnancies, births, stillbirth, premature birth, neonatal mortality. miscarriage and pregnancy termination, employment and salary level, health care utilization, and knowledge of and use of modern contraceptives. If the girl cannot be reached via phone, field-based research assistants will attempt to find her |
| **Case report form** | We will keep an electronic Case report form (CRF) for each participant where we will record information from the eligibility screening, contact information, consent issues, social problems experienced due to the trial, cash transfer issues, reasons for loss to follow-up etc. Information about the attendance in youth club meetings in the community component will be recorded on paper forms and will be entered into the electronic case report form for each participant in the combined intervention arm. Information about cash transfers and payment of school fees will also be recorded electronically and this information will automatically be updated in the electronic case report form. |

It seems that participants who are lost to follow-up are particularly likely to be married, and since we know that marriage is closely associated with early childbearing, we are concerned that loss to follow-up may introduce bias in the measurements of the outcomes related to childbearing and marriage. Thus we are requesting permission to record information about marital status and childbearing of participants who have been lost to follow-up. This information will be used in a secondary analysis of the proportion who has given birth and who are married.

## Data management and quality assurance

All the quantitative tools will be piloted before the data collections start to ensure that the questions and the translations are comprehendible and that the type of responses obtained are as anticipated.

Names, telephone numbers and addresses will be recorded in order to keep track of the girls, but will be kept separate from other data and will only be available to particular staff. Each participant will be given a unique numeric identifier, and this number will be used when storing forms and recordings of interviews. The file linking the unique identifier with the name will be password protected and stored on a password protected computer. Only the data manager, the Principal Investigator and the local PI will have access to the linking file.

Pictures of the participants are personal information and they will just used for the purpose of ensuring that the correct participants are interviewed by limiting access to the pictures to a few project staff. The pictures of the participants will be taken with the password-protected tablets and named, and they will transferred to the project's password protected hard drive (which is locked in a safe in the project's Lusaka office) at the end of the interview round. When the pictures have been transferred to the hard drive, they will be deleted from the tablet. We will check that the dates of the pictures match with the dates when the interviews were conducted. We would like to store the pictures until the end of 2020 so we can confirm that it is the same person that is interviewed in each of the last rounds. At the end of the trial in December 2020, all the pictures will be deleted.

All collected consent and data forms will be stored in a locked cabinet or on password protected computers or tablets in a locked office. The offices (one in each district and one in Lusaka) where forms and computers are stored will only be available to senior study staff and will be locked whenever the staff leave. There will be 24 hour security measures to prevent that anyone breaks in to the office. When the trial is completed, all personally identifiable information will be deleted.

To ensure that data collectors are familiarized with the tablets and the software, data collectors will undergo thorough training in the use of the data collection software, and the interviews will be piloted. The forms will have check- and skip-rules inbuilt to minimize data entry errors. Checks for outliers and inconsistencies will be automatically conducted during data entry. Manual checks for outliers and missing entries will also be done by the data manager after the daily uploading of electronic data from the tablets to the database via internet. The plan was initially to store the data on a server owned by the University of Zambia, but since we think the data security will be better if we save the date on a secure server owned by the University of Bergen, we have obtained approval from the University of Zambia Bioethical Research Ethics Committee (UNZABREC) to save the data on a University of Bergen server instead.

In the qualitative process evaluation, we will seek permission to audio record the individual interviews and focus group discussions using digital recorders. Systematic note taking will be employed (field notes) in connection with observations and during the interviews where audio recording will not take place. The data from the individual interviews and focus group discussions will be transcribed and translated by professional transcribers. A senior researcher will on a daily basis review the transcripts to ensure that they contain word by word transcriptions and translations to English that retain original meanings with a particular caution to retain culturally embedded content.

Electronic data without personal identifiable information (entered quantitative data or digital recordings) will be stored in at least two locations at all times to ensure that there is always a back-up available. Further details of the data management procedures can be found in the Manual of operations.

## Statistical methods

The outcomes will be captured in binary, continuous or a time-to-event variables. Continuous variables that are right skewed will be log-transformed to obtained more symmetrical distributions. We will correspondingly employ Poisson regression or General linear models (GLM) of the binomial family with a log link (estimating risk ratios or RRs) and identity link (estimating absolute risk reduction), linear regression (estimating average effect or mean increase or decrease) or survival analysis using Cox regression. Each method will take account of the design effect of the clustering.

The data will be analysed with Stata (Stata Corporation, TX, USA) statistical software. We will compare outcomes between the three arms: the economic arm vs. the control arm, the combined arm vs the control arm, and the economic arm vs the combined intervention arm. Analyses will be by intention-to-treat (ITT), that is all participants, irrespective of whether they actually agree to the intervention allocated to them, will be included in the analysis according to the intervention arm the cluster they live in was allocated to. The Intracluster correlation (ICC) will be reported for all primary and secondary outcomes.

The primary outcomes will be measured in the final survey in February 2020 based on the participants’ responses to the questions “Have you ever given birth?” and “If yes, on which date and which year did you give birth?”. Reported childbearing before July 2019 will be recorded as “birth 8 months after end of the intervention period”. Girls who report a date of childbearing preceding their 18^th^ birthday, will be recorded as having “birth before 18^th^ birthday”. Girls who are married at the start of the intervention, will be excluded from the analysis of proportion of girls ever married/cohabiting before their 16^th^ or 18th birthday.

Descriptive statistics, such as means or medians and standard deviation or interquartile range will be used to describe continuous variables (the choice depending on the distributions). For categorical variables, frequencies and percentages will be calculated.

Proportions will be compared on both absolute and relative scales. Thus, relative risk, RR (and its analogue effectiveness, 1-RR) and absolute risk reduction (and its reciprocal “Numbers needed to prevent”) and their 95% CIs will be estimated using generalized linear models (GLM) of the binomial family with a log link and identity link, respectively. We will also employ logistic regression to arrive at ORs for the binary outcomes. Cox regression analyses will be used for estimating the effect of our interventions on time to first pregnancy. We will in all the analyses account for the cluster effect using robust standard errors. Random effects models will be used to assess population effects whereas fixed effects models will be used to assess individual effects. We will check whether the model fit is good by comparing the estimates from the GLM models with estimates obtained using Generalised Estimating Equations (GEE).

Where necessary we will deal with multiple absorbing events (for competing risks) and left censoring. For all these models, either Population-averaged or marginal versus subject-specific or conditional survival probabilites will be estimated.

Equity effects will be examined by comparing differential effects on the primary outcomes between wealth tertiles and educational tertiles.

Although this is a large randomized trial and randomization will be constrained to achieve balance with respect to predictors of outcome, any remaining imbalances of predictors of the outcomes in question will be adjusted for in the regression models. In order to evaluate potential confounding, we will stratify the analyses on baseline characteristics that are or can be dichotomized, and if the stratified analyses give indications of confounding rather than effect measure modification, we will adjust the analyses for these baseline characteristic if their inclusion in the statistical model changes the effect estimate with more than 5% or substantially increases its statistical precision. For continuous variables such as age, we will also explore whether there is effect measure modification using appropriate interaction terms and, if not, adjust for variables using the criteria described above. For analysis where adjustment for several baseline characteristics turn out to be necessary, e.g. in (predefined) sub-group analyses, we will use statistical modelling approaches, such as those using the Akaike information criterion to arrive at a final model.

Interaction analysis will primarily be conducted on an additive scale, according to Rothman (Kenneth R. Rothman-Epidemiology, an introduction) and Anderson *et al*. (European Journal of Epidemiology (2005) 20: 575-579)

Instrumental variable analyses will be used in an attempt to estimate the causal effects of exposure to each intervention component (X) on the outcome (Y). (The random allocation (Z) will be the instrument.)

## Cost-Benefit Analysis (CBA)

The cost benefit analysis will utilise trial outcomes and secondary data to calculate the Net Societal Benefits (NSB) of the intervention alternatives, using the following formula

$${{NSB}_{i}=\sum_{t=1}^{n} \frac{b_{i}\left( t \right)-c_{i}(t)}{{(1+r)}^{t-1}}}$$

Where,

NSB_i_ = Net societal benefit of the intervention for all girls *i* (discounted)

b_i_(t) = benefits (in monetary terms) associated with offering the intervention to girl *i* derived in year t

c_i_(t) = costs (in monetary terms) associated with offering the intervention to girl *i* in year t

1/(1+r) = discount factor on annual interest rate r

N = lifetime of the individuals

Benefits and costs will be estimated from the perspectives of the family, community, school, health care and the government, and from society as a whole. At the family level, benefits will be the cash transfers, reduced school-related costs for daughters, the value of improved health of daughter and her future children, reduced or delayed user fees and transport costs for health care services, and increased future income. The cost at family level will be delayed receipt of a bride price for the daughter. At the level of the local community there will be costs related to community members attending community meetings, and there will be benefits from increased productivity. At the health care level, there will be reduced costs if the need for health care decreases with a reduction in the number of premature or low birth weight babies, or if the morbidity is lower among children of women who have received the intervention. Prospective costing techniques will be used alongside the main trial to estimate costs of the intervention alternatives, while other trial outcomes will be valued and combined to estimate long term benefits. Valuation of health benefits will be based on incidence data from the trial combined with disease cost information from the literature. Differences in school attainment between the trial arms will be used to extrapolate long term educational outcomes, while its valuation in terms of increased future income at household level will be based on secondary data.

To assess whether cost-benefits are equitably distributed, we will do regression analysis, with NSB as dependent variable, and socio-economic status and intervention arm as independent variables.

## Qualitative Data Analysis

The content of the qualitative data from interviews and focus group discussions will be explored at the end of each working day, the researchers will reflect on the emerging material, and adjust the interview guides to enhance their relevance in subsequent days. The transcribed and translated texts will be entered into QSR NVIVO 10, an electronic program for qualitative data management. The analysis of the interviews, FGDs, open-ended questions from log forms and observations will follow a classical approach employing Malterud’s ‘Systematic text condensation’ [34], a descriptive and explorative method for thematic cross-case analysis drawing upon Giorgi’s psychological phenomenological analysis. It is a strategy for analysis developed from traditions shared by other methods for analysis of qualitative data. Systematic text condensation consists of the following four steps: 1) total impression – from chaos to themes; 2) identifying and sorting meaning units – from themes to codes; 3) condensation – from code to meaning; 4) synthesizing – from condensation to descriptions and concepts.

## Data monitoring

A Data Monitoring Committee (DMC) will be established. The DMC will be composed of three members – a statistician (Simon Cousens), an epidemiologist (Kamija Phiri) and a social scientist (Anitha Menon). The committee members will be asked to declare any no conflict of interest they may have, and the committee will be independent of the project management team. Meetings will take place usually once a year. The committee will advise the project management on study modification or termination based on its reviews of data and pre-established statistical stopping rules. A charter for the DMC has been developed.

The DMC will be informed about the percentage of communities and girls/families that have consented/assented after the end of the recruitment period. If a school/cluster withdraws from the study, the DMC will be informed immediately and asked to give advice. If <90% of schools in each of the three arms still agree to participate after randomization, or if the difference between the highest and lowest cluster-level non-consent is >5 percentage points, the team will submit a plan for further study enrollment to the Data Monitoring Committee (DMC). The DMC will every six months be informed about the follow-up percentage for the last follow-up contact and survey. If the follow-up percentage is lower than 90 or if the attrition differs with 5 percentage points between any of the three trial arms, the principal investigators must submit a plan for corrective measures to the DMC.

## Harms

This study does not include a biomedical intervention and thus standard adverse event reporting will not be necessary. Unanticipated problems or serious adverse events that may be related to study participation will be recorded and reported to the ethical committees (UNZA BREC and REK-West) and the Data Monitoring Committee annually. The participants may experience social problems due to study participation, e.g. negative reactions or bullying from boys or other girls who do not receive the interventions. Previous studies of cash transfer programmes have not found such negative effects, but it is important to monitor such events [56]. Thus questions about negative experiences will be asked in the follow-up contacts. If the participant reports social problems caused by study participation (spontaneously or when asked about it) to any of the study staff, this will be recorded in the case report form and the girls will be counselled about how to deal with the problem. If the girl seems to be in danger, she will be referred to persons or institutions that can ensure her safety. If many girls in the same cluster report social problems caused by study participation, community meetings will be held to discuss how this can be handled.

## Benefits

The study team will not offer medical services, but will ensure appropriate referral to counselling services and /or medical care when required. Participants in the intervention arms will benefit from the study with respect to what the intervention packages will be offering. If the intervention packages are found to have a positive impact and the study contributes to policy change or introduction of similar government programmes, adolescents and their families in many communities may benefit.

## Auditing

Girls and boys who attend the youth club will be asked to sign an attendance list at every meeting. This attendance list together with a short log filled in by the teachers about the topics discussed and other activities that have taken place in the meeting, will be used as documentation that the meeting has taken place. The trial supervisor will bring a camera to all community and parent meetings to photographically document that these take place, and will also record how many turned up at these meetings and the topics discussed. She/he will also fill in an electronic form where GPS coordinates are automatically captured to verify the place and time of the meetings. The trial supervisor will monitor that the youth club meetings take place according to the plan and will evaluate whether the content and quality of the meetings are as intended

The CISMAC Executive committee or hired external experts will conduct a site readiness monitoring visit and annual monitoring visits during Study implementation. During the site readiness monitoring visit, the monitor will complete a standard site readiness checklist. If readiness is not evaluated to be satisfactory, the PI or a PMT member designated by the PI will together with the monitor develop a plan of action to be implemented before the start of the intervention. Checklists for quality control of Study procedures will be developed for the annual monitoring visits. If weaknesses or challenges are detected, a plan of action to address these will be prepared.

# 3. ETHICS AND DISSEMINATION

## Research ethics approval

The protocol has been approved by the Biomedical research ethics committee (BREC) at UNZA and the Regional Ethical Committee of Western Norway (REK-West). Permissions have been obtained from the MoE and MoH in Zambia and relevant district authorities to conduct the trial in schools in the intervention clusters and to engage teachers and CHAs/CHWs in the community component.

## Protocol amendments

Important protocol modifications will be reported to the UNZA-BREC, REK-West and clinicaltrials.gov.

The intervention will be adaptive, i.e. elements in the intervention package delivered to a cluster will be modified if obstacles to the implementation of the intervention are encountered, the participation is suboptimal or if important changes occur in the context. In a cluster randomized trial with an adaptive intervention, predetermined decisions rules that specify whether, how or when to modify the intervention that a community receives, are applied at critical decision points [57]. We provide three such examples: In intervention clusters where >10% of the participants report problems relating to receiving the cash transfers or claim they are not benefitting from the cash transfer, an alternative system for handing out the cash to girls and parents/guardians will be set up, such as a local committee consisting of three PTA members. If >20% of girls in a cluster report repeated bullying or harassment from peers or adults because of trial participation, meetings will be held with the community to discuss how to deal with this.

It may also be necessary to make some adjustments to the follow-up plans if we experience more attrition of participants than expected. One example is that the compensation for answering questions during the follow-up contacts or the surveys may be increased if it turns out to be difficult to get participants to take time to respond.

When the PI and the Project Management Team believe it is necessary to make substantial adaptions to the intervention, the DMC will be asked to critically consider whether the proposed adaptions are called for and are sufficient.

## Consent or assent

The eligible participants will receive thorough information about the study from a research assistant. When eligible participants are younger than 18 years, parental/guardians’ consent will be sought before the girl is asked to assent. Girls who do not assent/consent and those girls whose parents/guardians decline, will not be included in the study. Since some girls and parents/guardians may be sceptical to the intervention programme, but not to being interviewed, we will inform those who are reluctant to consent/assent that they can participate in the trial even if they do not want to receive the intervention. There will be no disadvantages to those who do not participate, and participants can withdraw from the study at any point in time. Head teachers, other teachers, headmen and other influential leaders will be asked not to put undue pressure on girls and parents to participate.

We will ask for written permission from each of the participants and their guardians for the research assistants to take selfies with them after the interview. The participants will be told that they are free to refuse to be photographed and if they do refuse, this will have no consequences for their participation.

## Confidentiality

All interviews will be conducted in privacy and strict confidentiality will be kept. Only authorized persons with permission from the PI will have access to sensitive information.

## Declaration of interests

No interests declared.

## Access to data

Access to and ownership of data will be based on the principles laid down by the CISMAC consortium agreement, and its annex 6.

## Dissemination plan

We will publish the protocol for the trial and findings from the trial in high-ranking journals. Findings from the formative phases and the pilot study will also be published in peer-reviewed journals. In addition, substantial dissemination of the research findings will be done through reports and presentations at national and international academic- and policy related conferences. Authorship of scientific papers emerging from the Study will be decided upon as per the CISMAC consortium agreement and its annex 5.

We have established an advisory group with representatives from the MoE, MoH, Ministry of Gender, and the Ministry of Traditional Affairs. This group will be called for regular meetings to provide guidance and assist with the community sensitization as part of the preparation for and during the trial. We will share and discuss preliminary and final research findings with the advisory group and other key stakeholders in Zambia and in meetings with the intervention communities.

# 4. RESEARCH TRAINING OPPORTUNITIES OFFERED BY THE STUDY

One PhD position (in epidemiology or medical anthropology) and 2.5 postdoctoral positions (in epidemiology, behavioural economics or medical anthropology, and health economics, respectively) have been allocated to the project from CISMAC resources. One PhD candidate from the project has been enrolled in the PhD programme at UiB with a Quota scholarship (from Statens Lånekasse). At least two medical research track students from UiB will be linked to the trial, and we also plan to involve several master students from UNZA and UiB.

# References

1. Patton, G.C., C. Coffey, S.M. Sawyer, R.M. Viner, D.M. Haller, K. Bose, T. Vos, J. Ferguson, and C.D. Mathers, *Global patterns of mortality in young people: a systematic analysis of population health data.* Lancet, 2009. **374**(9693): p. 881-92.

2. Paranjothy, S., H. Broughton, R. Adappa, and D. Fone, *Teenage pregnancy: who suffers?* Arch Dis Child, 2009. **94**(3): p. 239-45.

3. Scholl, T.O., M.L. Hediger, and D.H. Belsky, *Prenatal care and maternal health during adolescent pregnancy: a review and meta-analysis.* J Adolesc Health, 1994. **15**(6): p. 444-56.

4. March of Dimes, PMNCH, Save the Children, and WHO, *Born Too Soon: The Global Action Report on Preterm Birth*, M.K. CP Howson, JE Lawn. , Editor. 2012, World Health Organization: Geneva.

5. WHO, *WHO guidelines on preventing early pregnancy and poor reproductive health outcomes among adolescents in developing countries*. 2011, World Health Organization.

6. UNFPA, *Motherhood in Childhood. Facing the challenge of adolescent pregnancy. The State of World Population 2013*. 2013, UNFPA.

7. Central Statistical Office (CSO), Ministry of Health (MOH), Tropical Diseases Research Centre (TDRC), University of Zambia, and Macro International Inc, *Zambia Demographic and Health Survey 2007*. 2009, CSO, Macro International Inc: Calverton, Maryland, USA.

8. Kenya National Bureau of Statistics (KNBS) and ICF Macro, *Kenya Demographic and Health Survey 2008-09*. 2010, KNBS and ICF Macro: Calverton, Maryland.

9. National Statistical Office (NSO) and ICF Macro, *Malawi Demographic and Health Survey 2010*. 2011, NSO and ICF Macro: Zomba, Malawi, and Calverton, Maryland, USA.

10. Guttmacher Institute, *Into a new world: young women’s sexual and reproductive lives*. 1998, AGI: New York.

11. Gakidou, E., K. Cowling, R. Lozano, and C.J.L. Murray, *Increased educational attainment and its effect on child mortality in 175 countries between 1970 and 2009: a systematic analysis.* The Lancet. **376**(9745): p. 959-974.

12. Warrington, M. and S. Kiragu, *“It makes more sense to educate a boy”: Girls ‘against the odds’ in Kajiado, Kenya.* International Journal of Educational Development, 2012. **32**(2): p. 301-309.

13. Pengele, M. and C. Mazimba, *Promoting Family Planning in Zambia. Baseline study report.* 2013, Scaling Up Family Planning Services in Zambia (SUFP) Program, DFID.

14. Moore, A.M., A.E. Biddlecom, and E.M. Zulu, *Prevalence and Meanings of Exchange of Money or Gifts for Sex in Unmarried Adolescent Sexual Relationships in Sub-Saharan Africa.* African Journal of Reproductive Health / La Revue Africaine de la Santé Reproductive, 2007. **11**(3): p. 44-61.

15. Oringanje, C., M. Meremikwu Martin, H. Eko, E. Esu, A. Meremikwu, and E. Ehiri John *Interventions for preventing unintended pregnancies among adolescents*. Cochrane Database of Systematic Reviews, 2009. DOI: 10.1002/14651858.CD005215.pub2.

16. McQueston, K., R. Silverman, and A. Glassman, *The efficacy of interventions to reduce adolescent childbearing in low- and middle-income countries: a systematic review.* Stud Fam Plann, 2013. **44**(4): p. 369-88.

17. Malhotra, A., A. Warner, A. McGonagle, and S. Lee-Rife, *Solutions to End Child Marriage. What the evidence shows*. 2011, International Center for Research on Women (ICRW).

18. Baird, S., E. Chirwa, C. McIntosh, and B. Ozler, *The Short-Term Impacts of a Schooling Conditional Cash Transfer Program on the Sexual Behavior of Young Women.* Health Economics, 2010. **19**: p. 55-68.

19. Baird, S., C. McIntosh, and B. Ozler, *Cash or condition ? evidence from a randomized cash transfer program*. 2010.

20. Baird, S.J., R.S. Garfein, C.T. McIntosh, and B. Ozler, *Effect of a cash transfer programme for schooling on prevalence of HIV and herpes simplex type 2 in Malawi: a cluster randomised trial.* Lancet, 2012. **379**(9823): p. 1320-9.

21. Duflo, E. *Education and HIV/AIDS prevention: evidence from a randomized evaluation in Western Kenya. (Background Paper to the 2007 World Development Report WPS4024)*. Policy Research Working Paper Series 4024, The World Bank, 2006.

22. Doyle, A.M., et al. *Long-term biological and behavioural impact of an adolescent sexual health intervention in Tanzania: follow-up survey of the community-based MEMA kwa Vijana Trial*. PLoS medicine, 2010. **7**, e1000287.

23. Dupas, P., *Do Teenagers Respond to HIV Risk Information? Evidence from a Field Experiment in Kenya.* American Economic Journal-Applied Economics, 2011. **3**(1): p. 1-34.

24. Ross, D.A., et al. *Biological and behavioural impact of an adolescent sexual health intervention in Tanzania: a community-randomized trial*. AIDS (London, England), 2007. **21**, 1943-55 DOI: 10.1097/QAD.0b013e3282ed3cf5.

25. Hallfors, D., H. Cho, S. Rusakaniko, B. Iritani, J. Mapfumo, and C. Halpern *Supporting adolescent orphan girls to stay in school as HIV risk prevention: evidence from a randomized controlled trial in Zimbabwe*. American journal of public health, 2011. **101**, 1082-8.

26. Duflo, E., P. Dupas, M. Kremer, and S. Sinei, *Education and HIV/AIDS Prevention: Evidence from a randomized evaluation in Western Kenya*, in *World Bank Policy Research Working Paper* 2006, World Bank.

27. Duflo, E., P. Dupas, and M. Kremer, *Education, HIV, and Early Fertility: Experimental Evidence from Kenya.* American Economic Review, 2015. **105**(9): p. 2757-97.

28. Duflo, E., P. Dupas, M. Kremer, and S. Sinei, *Education and HIV/AIDS prevention : evidence from a randomized evaluation in Western Kenya*. 2006.

29. Underhill, K., D. Operario, and P. Montgomery *Abstinence-only programs for HIV infection prevention in high-income countries*. Cochrane Database of Systematic Reviews, 2007. DOI: 10.1002/14651858.CD005421.pub2.

30. Plummer, M.L., et al., *A process evaluation of a school-based adolescent sexual health intervention in rural Tanzania: the MEMA kwa Vijana programme.* Health Educ Res, 2007. **22**: p. 500–512.

31. Wamoyi, J., G. Mshana, A.M. Doyle, and D.A. Ross, *Recall, relevance and application of an in-school sexual and reproductive health intervention 7–9 years later: perspectives of rural Tanzanian young people.* Health Promotion International, 2013. **28**(3): p. 311-321.

32. Banda, R., K. Fylkesnes, and I.F. Sandøy, *Rural-urban differentials in pregnancy-related mortality in Zambia: estimates using data collected in a census.* Population health metrics, 2015. **13**: p. 32.

33. Crosby, R. and S.M. Noar, *What is a planning model? An introduction to PRECEDE-PROCEED.* Journal of Public Health Dentistry, 2011. **71**: p. s7-s15.

34. Malterud, K., *Systematic text condensation: A strategy for qualitative analysis sation.* Scand J Public Health, 2012. **40**(8): p. 795-805.

35. Albrecht, S., B. Cassidy, D. Salamie, and M.D. Reynolds, *A smoking cessation intervention for pregnant adolescents: implications for nurse practitioners.* Journal of the American Academy of Nurse Practitioners, 1999. **11**(4): p. 155-9.

36. Hardeman, W., M. Johnston, D.W. Johnston, D. Bonetti, N.J. Wareham, and A.L. Kinmoth, *Application of the Theory of Planned Behaviour in behaviour change interventions: A systematic review.* Psychol Health, 2002. **17**(2): p. 123-158.

37. Zulu, J. and V. Peleka, *Strengthening Cash Transfers for Access to Finance, Livelihood and Entrepreneurship: Baseline Report.* 2013, Care International Zambia: Lusaka.

38. Reimers, F., C. DeShano da Silva, and E. Trevino, *Where is the “Education” in Conditional Cash Transfers in Education?*, in *UIS Working Paper*, U.I.f. Statistics, Editor. 2006, UNESCO Institute for Statistics: Montreal.

39. Schultz, P.T., *School subsidies for the poor: evaluating the Mexican Progresa poverty program.* Journal of Development Economics, 2004. **74**(1): p. 199-250.

40. Dubois, P., A. de Janvry, and E. Sadoulet, *Effects on School Enrollment and Performance of a Conditional Cash Transfer Program in Mexico.* Journal of Labor Economics, 2012. **30**(3): p. 555-589.

41. Banerjee, A., P. Glewwe, S. Powers, and M. Wasserman, *Expanding Access and Increasing Student Learning in Post-Primary Education in Developing Countries: A Review of the Evidence*, in *Post-Primary Education Initiative* A.L.J.P.A.L. (J-PAL), Editor. 2013.

42. Chaaban, J. and W. Cunningham *Measuring the Economic Gain of Investing in Girls. The Girl Effect Dividend*. Policy Research Working Paper Series, 2011.

43. Schäferhoff, M., et al., *Estimating the costs and benefits of education from a health perspective» Background paper for the Oslo Summit on Education for Development. Executive Summary.* 2015.

44. Handa, S., D. Seidenfeld, B. Davis, G. Tembo, and the Zambia Cash Transfer Evaluation Team, *Are Cash Transfers a Silver Bullet? Evidence from the Zambian Child Grant*, in *Innocenti Working Paper*. 2014: Florence, Italy.

45. Kenya Adolescent Reproductive Health Program, Program for Appropriate Technology in Health (PATH), and Population Council, *Tuko Pamoja. Adolescent Reproductive Health and Life Skills Curriculum*. 2006: Nairobi, Kenya.

46. (MOH), M.o.H., *National Community Health Worker Strategy in Zambia.*, MOH, Editor. 2010: Lusaka.

47. Zulu, J.M., J. Kinsman, C. Michelo, and A.-K. Hurtig, *Developing the national community health assistant strategy in Zambia: a policy analysis.* Health Res Policy and Sys 2013. **11**: p. 24.

48. Zulu, J., J. Kinsman, C. Michelo, and A.-K. Hurtig, *Hope and despair: community health assistants’ experiences of working in a rural district in Zambia.* Hum Resour Health, 2014. **12**(1): p. 30.

49. Smith, M.K. *Paulo Freire and informal education*. The encyclopaedia of informal education 2002; Available from: <http://infed.org/mobi/paulo-freire-dialogue-praxis-and-education/>.

50. Lewin, K., *Forces behind food habits and methods of change.* Bulletin of the National Research Council, 1943. **108**: p. 35-65.

51. Hayes, R.J. and L.H. Moulton, *Cluster randomised trials*. Interdisciplinary Statistics 2009, Boca Raton: Chapman & Hall/CRC.

52. Langhaug, L.F., L. Sherr, and F.M. Cowan, *How to improve the validity of sexual behaviour reporting: systematic review of questionnaire delivery modes in developing countries.* Trop Med Int Health, 2010. **15**(3): p. 362-81.

53. Langhaug, L.F., Y.B. Cheung, S.J. Pascoe, P. Chirawu, G. Woelk, R.J. Hayes, and F.M. Cowan, *How you ask really matters: randomised comparison of four sexual behaviour questionnaire delivery modes in Zimbabwean youth.* Sex Transm Infect, 2011. **87**(2): p. 165-73.

54. Glynn, A.N., *What Can We Learn with Statistical Truth Serum?Design and Analysis of the List Experiment.* Public Opinion Quarterly, 2013. **77**(S1): p. 159-172.

55. Moseson, H., E. Treleaven, C. Gerdts, and N. Diamond-Smith, *The List Experiment for Measuring Abortion: What We Know and What We Need.* Studies in Family Planning, 2017. **48**(4): p. 397-405.

56. Pettifor, A., C. MacPhail, N. Nguyen, and M. Rosenberg, *Can money prevent the spread of HIV? A review of cash payments for HIV prevention.* AIDS and behavior, 2012. **16**(7): p. 1729-1738.

57. Brown, C.H., T.R.T. Have, B. Jo, G. Dagne, P.A. Wyman, B. Muthén, and R.D. Gibbons, *Adaptive Designs for Randomized Trials in Public Health.* Annual review of public health, 2009. **30**: p. 1-25.

# Appendix 1 – Project timelines

|  | 2015 | | 2016 | | | | | | | | | | | | 2017 | | | | | | | | | | | | 2018 | | | | | | | | | | | | 2019 | | | | | | | | | | | | 2020 | | | | | | | | | | |
| --- | --- | --- | --- | --- | --- | --- | --- | --- | --- | --- | --- | --- | --- | --- | --- | --- | --- | --- | --- | --- | --- | --- | --- | --- | --- | --- | --- | --- | --- | --- | --- | --- | --- | --- | --- | --- | --- | --- | --- | --- | --- | --- | --- | --- | --- | --- | --- | --- | --- | --- | --- | --- | --- | --- | --- | --- | --- | --- | --- | --- | --- |
|  | N | D | J | F | M | A | M | J | J | A | S | O | N | D | J | F | M | A | M | J | J | A | S | O | N | D | J | F | M | A | M | J | J | A | S | O | N | D | J | F | M | A | M | J | J | A | S | O | N | D | J | F | M | A | M | J | J | A | S | O | N |
| **Preparation phase** |  |  |  |  |  |  |  |  |  |  |  |  |  |  |  |  |  |  |  |  |  |  |  |  |  |  |  |  |  |  |  |  |  |  |  |  |  |  |  |  |  |  |  |  |  |  |  |  |  |  |  |  |  |  |  |  |  |  |  |  |  |
| Recruitment of trial supervisors | X |  |  |  |  |  |  |  |  |  |  |  |  |  |  |  |  |  |  |  |  |  |  |  |  |  |  |  |  |  |  |  |  |  |  |  |  |  |  |  |  |  |  |  |  |  |  |  |  |  |  |  |  |  |  |  |  |  |  |  |  |
| Recruitment of district accountants |  | X |  |  |  |  |  |  |  |  |  |  |  |  |  |  |  |  |  |  |  |  |  |  |  |  |  |  |  |  |  |  |  |  |  |  |  |  |  |  |  |  |  |  |  |  |  |  |  |  |  |  |  |  |  |  |  |  |  |  |  |
| Training of district accountants |  | X |  |  |  |  |  |  |  |  |  |  |  |  |  |  |  |  |  |  |  |  |  |  |  |  |  |  |  |  |  |  |  |  |  |  |  |  |  |  |  |  |  |  |  |  |  |  |  |  |  |  |  |  |  |  |  |  |  |  |  |
| Training of trial supervisors | X |  |  |  |  |  |  |  |  |  |  |  |  |  |  |  |  |  |  |  |  |  |  |  |  |  |  |  |  |  |  |  |  |  |  |  |  |  |  |  |  |  |  |  |  |  |  |  |  |  |  |  |  |  |  |  |  |  |  |  |  |
| Finalize SOPs | X | X |  |  |  |  |  |  |  |  |  |  |  |  |  |  |  |  |  |  |  |  |  |  |  |  |  |  |  |  |  |  |  |  |  |  |  |  |  |  |  |  |  |  |  |  |  |  |  |  |  |  |  |  |  |  |  |  |  |  |  |
| Finalize consent and assent forms | X |  |  |  |  |  |  |  |  |  |  |  |  |  |  |  |  |  |  |  |  |  |  |  |  |  |  |  |  |  |  |  |  |  |  |  |  |  |  |  |  |  |  |  |  |  |  |  |  |  |  |  |  |  |  |  |  |  |  |  |  |
| Selection and set-up of data management system | X | X |  |  |  |  |  |  |  |  |  |  |  |  |  |  |  |  |  |  |  |  |  |  |  |  |  |  |  |  |  |  |  |  |  |  |  |  |  |  |  |  |  |  |  |  |  |  |  |  |  |  |  |  |  |  |  |  |  |  |  |
| Trial registration |  |  |  |  | X |  |  |  |  |  |  |  |  |  |  |  |  |  |  |  |  |  |  |  |  |  |  |  |  |  |  |  |  |  |  |  |  |  |  |  |  |  |  |  |  |  |  |  |  |  |  |  |  |  |  |  |  |  |  |  |  |
| **Orientation phase** |  |  |  |  |  |  |  |  |  |  |  |  |  |  |  |  |  |  |  |  |  |  |  |  |  |  |  |  |  |  |  |  |  |  |  |  |  |  |  |  |  |  |  |  |  |  |  |  |  |  |  |  |  |  |  |  |  |  |  |  |  |
| Headmaster orientation meeting | X | X | X | X | X | X |  |  |  |  |  |  |  |  |  |  |  |  |  |  |  |  |  |  |  |  |  |  |  |  |  |  |  |  |  |  |  |  |  |  |  |  |  |  |  |  |  |  |  |  |  |  |  |  |  |  |  |  |  |  |  |
| Chief orientation visit | X | X | X | X | X | X |  |  |  |  |  |  |  |  |  |  |  |  |  |  |  |  |  |  |  |  |  |  |  |  |  |  |  |  |  |  |  |  |  |  |  |  |  |  |  |  |  |  |  |  |  |  |  |  |  |  |  |  |  |  |  |
| Headmen orientation meeting | X | X | X | X | X | X |  |  |  |  |  |  |  |  |  |  |  |  |  |  |  |  |  |  |  |  |  |  |  |  |  |  |  |  |  |  |  |  |  |  |  |  |  |  |  |  |  |  |  |  |  |  |  |  |  |  |  |  |  |  |  |
| Religious leader, opinion leader and PTA orientation | X | X | X | X | X | X |  |  |  |  |  |  |  |  |  |  |  |  |  |  |  |  |  |  |  |  |  |  |  |  |  |  |  |  |  |  |  |  |  |  |  |  |  |  |  |  |  |  |  |  |  |  |  |  |  |  |  |  |  |  |  |
| Community sensitization meeting |  |  |  |  | X | X | X | X | X |  |  |  |  |  |  |  |  |  |  |  |  |  |  |  |  |  |  |  |  |  |  |  |  |  |  |  |  |  |  |  |  |  |  |  |  |  |  |  |  |  |  |  |  |  |  |  |  |  |  |  |  |
| **Recruitment and baseline phase** |  |  |  |  |  |  |  |  |  |  |  |  |  |  |  |  |  |  |  |  |  |  |  |  |  |  |  |  |  |  |  |  |  |  |  |  |  |  |  |  |  |  |  |  |  |  |  |  |  |  |  |  |  |  |  |  |  |  |  |  |  |
| Recruitment of research assistants to baseline survey |  |  |  | X |  |  |  |  |  |  |  |  |  |  |  |  |  |  |  |  |  |  |  |  |  |  |  |  |  |  |  |  |  |  |  |  |  |  |  |  |  |  |  |  |  |  |  |  |  |  |  |  |  |  |  |  |  |  |  |  |  |
| Training research assistants and TS (5 days in Lusaka) |  |  |  | X |  |  |  |  |  |  |  |  |  |  |  |  |  |  |  |  |  |  |  |  |  |  |  |  |  |  |  |  |  |  |  |  |  |  |  |  |  |  |  |  |  |  |  |  |  |  |  |  |  |  |  |  |  |  |  |  |  |
| Recruitment and baseline survey |  |  |  |  | X | X | X | X | X |  |  |  |  |  |  |  |  |  |  |  |  |  |  |  |  |  |  |  |  |  |  |  |  |  |  |  |  |  |  |  |  |  |  |  |  |  |  |  |  |  |  |  |  |  |  |  |  |  |  |  |  |
|  |  |  |  |  |  |  |  |  |  |  |  |  |  |  |  |  |  |  |  |  |  |  |  |  |  |  |  |  |  |  |  |  |  |  |  |  |  |  |  |  |  |  |  |  |  |  |  |  |  |  |  |  |  |  |  |  |  |  |  |  |  |
| **Randomization** |  |  |  |  |  |  |  |  | X |  |  |  |  |  |  |  |  |  |  |  |  |  |  |  |  |  |  |  |  |  |  |  |  |  |  |  |  |  |  |  |  |  |  |  |  |  |  |  |  |  |  |  |  |  |  |  |  |  |  |  |  |
| **Community/parent meetings to provide information about allocation after randomization** |  |  |  |  |  |  |  |  | X | X | X |  |  |  |  |  |  |  |  |  |  |  |  |  |  |  |  |  |  |  |  |  |  |  |  |  |  |  |  |  |  |  |  |  |  |  |  |  |  |  |  |  |  |  |  |  |  |  |  |  |  |
|  |  |  |  |  |  |  |  |  |  |  |  |  |  |  |  |  |  |  |  |  |  |  |  |  |  |  |  |  |  |  |  |  |  |  |  |  |  |  |  |  |  |  |  |  |  |  |  |  |  |  |  |  |  |  |  |  |  |  |  |  |  |
| **Economic component** |  |  |  |  |  |  |  |  |  |  |  |  |  |  |  |  |  |  |  |  |  |  |  |  |  |  |  |  |  |  |  |  |  |  |  |  |  |  |  |  |  |  |  |  |  |  |  |  |  |  |  |  |  |  |  |  |  |  |  |  |  |
| UCT |  |  |  |  |  |  |  |  |  |  | X | X | X | X | X | X | X | X | X | X | X | X | X | X | X | X | X | X | X | X | X | X | X | X | X | X | X |  |  |  |  |  |  |  |  |  |  |  |  |  |  |  |  |  |  |  |  |  |  |  |  |
| Payment of school fees |  |  |  |  |  |  |  |  |  |  |  |  |  |  | X |  |  |  |  |  |  |  |  |  |  |  | X |  |  |  |  |  |  |  |  |  |  |  |  |  |  |  |  |  |  |  |  |  |  |  |  |  |  |  |  |  |  |  |  |  |  |
| **Community component** |  |  |  |  |  |  |  |  |  |  |  |  |  |  |  |  |  |  |  |  |  |  |  |  |  |  |  |  |  |  |  |  |  |  |  |  |  |  |  |  |  |  |  |  |  |  |  |  |  |  |  |  |  |  |  |  |  |  |  |  |  |
| Selection of teachers, CHWs, youth peer educators |  |  |  |  |  |  |  |  | X | X |  |  |  |  |  |  |  |  |  |  |  |  |  |  |  |  |  |  |  |  |  |  |  |  |  |  |  |  |  |  |  |  |  |  |  |  |  |  |  |  |  |  |  |  |  |  |  |  |  |  |  |
| Training of teachers and CHWs and trial supervisors |  |  |  |  |  |  |  |  |  | X |  |  |  |  |  |  |  |  |  |  | X |  |  |  |  |  |  |  |  |  |  |  |  |  |  |  |  |  |  |  |  |  |  |  |  |  |  |  |  |  |  |  |  |  |  |  |  |  |  |  |  |
| Training of youth peer educators |  |  |  |  |  |  |  |  |  | X |  |  |  |  |  |  |  |  |  |  |  |  |  |  |  |  |  |  |  |  |  |  |  |  |  |  |  |  |  |  |  |  |  |  |  |  |  |  |  |  |  |  |  |  |  |  |  |  |  |  |  |
| Meetings in youth clubs |  |  |  |  |  |  |  |  |  |  | X | X | X |  | X | X | X |  | X | X | X |  | X | X | X |  | X | X | X |  | X | X | X |  |  |  |  |  |  |  |  |  |  |  |  |  |  |  |  |  |  |  |  |  |  |  |  |  |  |  |  |
| Community meetings |  |  |  |  |  |  |  |  |  |  | X |  | X |  | X |  | X |  | X |  | X |  | X |  | X |  | X |  | X |  | X |  | X |  | X |  | X |  |  |  |  |  |  |  |  |  |  |  |  |  |  |  |  |  |  |  |  |  |  |  |  |
| Parent meetings |  |  |  |  |  |  |  |  |  |  |  |  |  |  |  |  |  |  |  |  |  |  | X |  |  |  |  |  |  |  |  |  |  |  | X |  |  |  |  |  |  |  |  |  |  |  |  |  |  |  |  |  |  |  |  |  |  |  |  |  |  |
|  |  |  |  |  |  |  |  |  |  |  |  |  |  |  |  |  |  |  |  |  |  |  |  |  |  |  |  |  |  |  |  |  |  |  |  |  |  |  |  |  |  |  |  |  |  |  |  |  |  |  |  |  |  |  |  |  |  |  |  |  |  |
| **Follow-up contacts** |  |  |  |  |  |  |  |  |  |  |  | X | X | X |  |  |  | X | X | X | X |  | X | X | X | X |  | X | X | X | X | X |  | X | X | X | X | X |  |  | X | X | X | X |  |  | X | X | X | X |  | X | X | X | X |  |  |  |  |  |  |
|  |  |  |  |  |  |  |  |  |  |  |  |  |  |  |  |  |  |  |  |  |  |  |  |  |  |  |  |  |  |  |  |  |  |  |  |  |  |  |  |  |  |  |  |  |  |  |  |  |  |  |  |  |  |  |  |  |  |  |  |  |  |
| **Qualitative process evaluation** |  |  |  |  |  |  |  |  |  |  |  |  | X |  |  |  |  |  |  |  | X |  |  |  |  |  |  |  |  |  |  |  | X |  |  |  |  |  |  |  |  |  |  |  |  |  |  |  |  |  |  |  |  |  |  |  |  |  |  |  |  |
|  |  |  |  |  |  |  |  |  |  |  |  |  |  |  |  |  |  |  |  |  |  |  |  |  |  |  |  |  |  |  |  |  |  |  |  |  |  |  |  |  |  |  |  |  |  |  |  |  |  |  |  |  |  |  |  |  |  |  |  |  |  |
| **Final survey** |  |  |  |  |  |  |  |  |  |  |  |  |  |  |  |  |  |  |  |  |  |  |  |  |  |  |  |  |  |  |  |  |  |  |  |  |  |  |  |  |  |  |  |  |  |  |  |  |  |  |  |  |  |  |  |  | X | X | X | X | X |

# Appendix 2 – SRH Curriculum of youth club

We will base most of the sessions on the “Tuko Pamoja. Adolescent Reproductive Health

and Life Skills Curriculum” [45] with some few adaptions to fit the purpose of this trial.

| **FIRST CYCLE YOUTH MEETINGS**  1. Film on the importance of education.  2. The value of education  3. School drop-out (including re-entry)  4. Adolescence and puberty.  5. Reproduction Myths  6. Communication  7. Healthy Relationships  8. Film on early pregnancy and marriage.  9. Early pregnancy  10. Early marriage  11. Gender relations  12. Gender stereotypes  13. Peer pressure  14. Self-Esteem  15. Being Assertive  16. Decision Making  17. Setting Goals  18. Romantic Relationships  19. Love and Infatuation  20. Sexual behaviour and desire  21. Sexual decision making and abstinence.  22. Ways to prevent pregnancies including contraception.  23. Sexually transmitted infections (STIs)  24. HIV prevention | **SECOND CYCLE YOUTH MEETINGS**  1. The menstrual cycle and the risk of pregnancy   1. 2. Myths about sexuality, the risk of pregnancy and HIV 2. 3. How to prevent pregnancy 3. 4. How to deal with peer pressure 4. 5. Communication with friends and parents about feelings and relationships 5. 6. How to communicate with your boy/girlfriend about sensitive and emotional issues 6. 7. Self- Esteem 7. 8. The right to decide for yourself and to access information and services 8. 9. Unwanted sex and sexual abuse 9. 10. School re-entry policy and its effects 10. 11. Decision making and setting goals 11. 12. Summary of lessons and evaluation |
| --- | --- |

1. First conducted November 2013, updated in November 2014, using the search query ((pregnancy in adolescence[mesh]) OR ((Adolescent or teen or youth or youths or girls) AND (Pregnancy[mesh] or pregnan* or birth* or childbirth OR marriage or Marriage[mesh]))) AND (((intervention or program or programme or trial or experiment or experimental) AND (random*)) OR (Controlled Clinical Trial[mesh] or Clinical Trial[mesh] or Clinical Trial, Phase III[mesh] or Randomized Controlled Trial[mesh])). [↑](#footnote-ref-2)
2. Medline, Embase, Web of Science, PsycINFO, ERIC, EconLit, Cochrance, CINAHL, POPLINE, LiILAC, African Index Medicus, Index Medicus for the Eastern Mediterranean Region, Global Development Finance, Economic Outlook Database, and World Bank e-library. [↑](#footnote-ref-3)
3. The Theory of Planned Behaviour (TPB) can be used to identify predisposing, reinforcing and enabling factors 36. Hardeman, W., M. Johnston, D.W. Johnston, D. Bonetti, N.J. Wareham, and A.L. Kinmoth, *Application of the Theory of Planned Behaviour in behaviour change interventions: A systematic review.* Psychol Health, 2002. **17**(2): p. 123-158.. According to the TPB, people’s beliefs, attitudes, subjective norms, and perceived behavioural control are essential factors predicting behavioural intentions and actual behaviour. The theory focuses on three types of beliefs: behavioural (beliefs about positive or negative consequences of a behaviour), normative (perceptions about the social acceptability of a behaviour) and control (beliefs about facilitating factors and barriers in relation to performing the behaviour) 36. Ibid... [↑](#footnote-ref-4)
4. CHAs were established as a new cadre in 2010 in response to the scarcity of health facilities and personnel in rural areas. They go through a one year comprehensive training programme that comprises SRH services, incl. adolescent health services, gender issues and provision of family planning (46. (MOH), M.o.H., *National Community Health Worker Strategy in Zambia.*, MOH, Editor. 2010: Lusaka, 47. Zulu, J.M., J. Kinsman, C. Michelo, and A.-K. Hurtig, *Developing the national community health assistant strategy in Zambia: a policy analysis.* Health Res Policy and Sys 2013. **11**: p. 24.). Previously, CHWs only received a few weeks of training. CHAs are selected by their community (just like other CHWs) and spend most of their time working in the community. Currently about 600 CHAs have been trained and deployed (48. Zulu, J., J. Kinsman, C. Michelo, and A.-K. Hurtig, *Hope and despair: community health assistants’ experiences of working in a rural district in Zambia.* Hum Resour Health, 2014. **12**(1): p. 30.). [↑](#footnote-ref-5)
5. **Changes to the protocol**

   The following changes were made to the protocol after the start of trial recruitment in March 2016:

   **Study setting and size**

   The average cluster size turned out to be smaller than anticipated (31 instead of 37) and in some of the schools we had invited, we did not obtain assent/consent from a sufficient proportion of the eligible participants (see Figure 1). We compensated by including 157 (instead of 153) schools. The number of study districts was increased from 8 to 12 to find enough schools that were at least 8 km apart. (This change was made in May 2016, i.e. before randomization).

   **Outcomes**

   - Changed birth measures from proportions to incidence rates (in July 2016)
   - Added the secondary outcome “Socioeconomic inequality in incidence of marriage/ cohabitation before girls’ 18th birthday” (in November 2016).
   - Changed marriage measures from proportions to incidence rates (in October 2020) as we realized it should be measured in a similar way to the birth outcomes.

   **Interventions**

   - The number of annual community meetings in the combined arm was increased from four to six (change made in July 2016, i.e. before the start of the intervention period)

   **Follow-up interviews**

   - The main interview mode changed from being via telephone to being face-to-face because it turned out to be more difficult than anticipated to reach the participants via phone since network problems were common, most of them did not have their own phones, and they were often not together with their guardians (who had phones) during the day.
   - Audio Computer Assisted Self-Interviewing was employed for sensitive questions from the fourth follow-up round.
   - The transport allowance/compensation provided for participating in the follow-up rounds was increased from ZMW 20 to ZMW 50 in 2018, ZMW 75 in 2019 and ZMW 100 in 2020 because an increasing proportion of the participants moved away.
   - We obtained additional funding to interview guardians about household expenditures in 2018.
   - From 2019 the research assistants were instructed to take selfies of themselves with the participants to document that they had interviewed the correct participants (and had not been tempted to fabricate interviews when they were asked to visit the participants´ homes without a supervisor accompanying them).
   - From 2019 we recorded information from teachers, neighbours and family members on marital status and childbearing of participants who were lost to follow-up to use this information in sensitivity analyses.

   All the changes were approved by the ethics committe [↑](#endnote-ref-2)
